# *Knoetschkesuchus langenbergensis* gen. nov. sp. nov., a new atoposaurid crocodyliform from the Upper Jurassic Langenberg Quarry (Lower Saxony, northwestern Germany), and its relationships to *Theriosuchus*

**DOI:** 10.1371/journal.pone.0160617

**Published:** 2017-02-15

**Authors:** Daniela Schwarz, Maik Raddatz, Oliver Wings

**Affiliations:** 1 Museum fuer Naturkunde Berlin, Leibniz Institute for Evolutionary and Biodiversity Research, Berlin, Germany; 2 Department of Natural Sciences, Landesmuseum Hannover, Hannover, Germany; College of the Holy Cross, UNITED STATES

## Abstract

We report a new, small-sized atoposaurid crocodyliform from the Upper Jurassic of Langenberg, Northeastern Germany. Atoposaurids are small-sized Mesozoic crocodyliforms of mainly European distribution, which are considered to be phylogenetically close to the origin of Eusuchia. *Knoetschkesuchus langenbergensis* gen. nov. sp. nov. is represented by two well-preserved skulls and additional cranial and postcranial remains representing different ontogenetic stages. 3D reconstructions of a juvenile skull based on micro-computed tomography allow the most detailed description of cranial remains of any atoposaurid hitherto presented. Our new analysis contradicts previous preliminary assignment of the Langenberg atoposaurids to *Theriosuchus*. *Knoetschkesuchus* gen. nov. is characterized in particular by the presence of two dental morphotypes in the maxilla and dentary, slit-like secondary choanae within a narrow groove on the surface of the pterygoid, absence of lacrimonasal contact, presence of an antorbital foramen and an external mandibular fenestra, and proportional characters of the interorbital and intertemporal region. A similar combination of characters allows attribution of *Theriosuchus guimarotae* to *Knoetschkesuchus*, forming the new combination *Knoetschkesuchus guimarotae*. Our analysis provides an osteological basis for the separation of *Theriosuchus* and *Knoetschkesuchus* and helps further delineate generic differences in other closely related crocodylomorphs. Our phylogenetic analysis corroborates inclusion of *Knoetschkesuchus* into Atoposauridae and supports a position of Atoposauridae within Eusuchia.

## Introduction

The discovery of the dwarfed sauropod dinosaur *Europasaurus holgeri* made the Langenberg Quarry a fossil locality of world-wide importance [1]. The marly limestones at the Langenberg near Goslar, deposited in a shallow marine inlet or a small marginal basin of the German Late Jurassic Basin [2, 3], yield a high diversity of invertebrate, micro- and macrovertebrate as well as plant fossils [4, 5]. Among these fossils are the remains of marine and terrestrial atoposaurid crocodylians [6–8], which here are described for the first time in detail for the first time. Previous preliminary description placed the specimens in the genus *Theriosuchus*, but provided only limited evidence for that determination [8].

Atoposauridae is a family of small sized crocodyliforms typically smaller than one meter in body length that was first described by the French palaeontologist Gervais in 1871 [9]. They were included into Crocodylia by the German palaeontologist Zittel in 1890 [10]. Atoposaurid crocodyliforms have a short snout with paired external nares, in relation to the overall skull proportions large orbits and relatively small supratemporal fenestrae in relation to the overall skull proportions, enlarged anterior maxillary teeth, an often reduced or closed antorbital fenestra, slender limbs, and occasionally reduced osteodermal armor [11–17]. According to Buscalioni and Sanz [17], Atoposauridae comprises the five “core” genera *Theriosuchus*, *Alligatorium*, *Alligatorellus*, *Montsecosuchus* and *Atoposaurus* [17, 18]. The monophyly of this group has been questioned [16], because both the skull morphology and the small body size are also a strong ontogenetic signal in morphology. Also, the majority of skeletons of atoposaurids have been found in limestone slabs and were preserved more or less flattened, further complicating morphological interpretation. In phylogenetic analyses, usually only the genus *Theriosuchus* is coded, sometimes also *Alligatorium* [19–25]. *Theriosuchus* itself is probably the best-known and best-preserved taxon within Atoposauridae [26, 27], although a good diagnosis of this taxon is still pending. Several characters separate*Theriosuchus* from other atoposaurids, which make an assignment to Atoposauridae uncertain [8, 16, 17, 19, 24, 28]. Detailed examination of new finds and thorough descriptions will help to shed new light on the taxonomic status of *Theriosuchus*.

Our re-examination of the atoposaurid specimens from Langenberg assigned to *Theriosuchus pusillus* (i.e., DFMMh/FV 200 and DFMMh/FV 605) [8] raised doubts regarding the validity of this determination. The exceptionally well preserved material warranted a more thorough description and a detailed comparison with other species of *Theriosuchus*. Although the Langenberg remains have been preparated meticulously, parts of the skulls remained in the sediment. Recently, micro-computed tomography (μCT) made it possible to study one of these skulls (DFMMh/FV 605) in more detail. We were able to add a lot of information to the anatomy of the specimens with the help of three-dimensional (3D) images obtained from the μCT studies. In summary, the “*Theriosuchus*” material from Langenberg provides new information on the characters included in a diagnosis of *Theriosuchus*, adds new information on different ontogenetic states and 3D studies of its anatomy, and in the end makes it possible to separate these specimens from *Theriosuchus*.

### Anatomical abbrevations used in figures

An, angular; aof, antorbital fenestra; boc, basioccipital; bsph, basisphenoid; bsphr, basisphenoid rostrum; can cq, cranioquadrate canal; caV, caudal vertebra; ch, secondary choanae; ceri, cervical rib; con spl, contact area of dentary to splenial; con san, contact area from angular to surangular; cond occ, occipital condyle; cpsoc, contact between parietal and supraoccipital; cr, crest; d, dentary; d sel, dorsum sellae; dentt, dentary teeth; dep palp, depression for palpebral attachment; dori, dorsal rib; doV, dorsal vertebra; ec, ectopterygoid; emf, external mandibular fenestra; exoc, exoccipital; f, frontal; fi, fibula; f mag, foramen magnum; fme, external mandibular fenestra; for, foramen; for car an, foramen caroticum anterior; for car post, foramen caroticum posterior; for eu lat, lateral Eustachian foramen; for eu med, medial Eustachian foramen; for nut, nutricious foramen; for ot, otic foramen; for vag, foramen vagi; for X, foramen for cranial nerve V; for VI, foramen for cranial nerve VI; for IX-XII, foramina for cranial nerves IX, X, XI, XII; fos cer, cerebral fossa; fos hyp, hypophyseal fossa; hu, humerus; il, ilium; ips, intraparietal suture; itf, infratemporal fenestra; j, jugal; lac, lacrimal; lsph, laterosphenoid; mand, mandible; max, maxilla; mc, metacarpal; mt, metatarsal; n, nasal; na, neural arch; ne, external naris; or, orbit; osd, osteoderm; p, parietal; pal, palatine; palp, palpebral; phal, phalanx; pmx, premaxilla; po, postorbital; prf, prefrontal; prfp, prefrontal pillar; proot, prooticum; pt, pterygoid; pt fl, pterygoid flange; qj, quadratojugal; qu, quadrate; qu cond, quadrate condyle; qu cr A, quadrate crest A; qu cr B, quadrate crest B; rad, radius; san, surangular; scap, scapula; sept, septum; soc, supraoccipital; sof, suborbital fenestra; sp, splenial; sq, squamosal; stf, supratemporal foramen; symph, mandibular symphysis; ti, tibia; tm1, tooth morph 1; tm2, tooth morph 2.

### Locality

**Fig 1:**
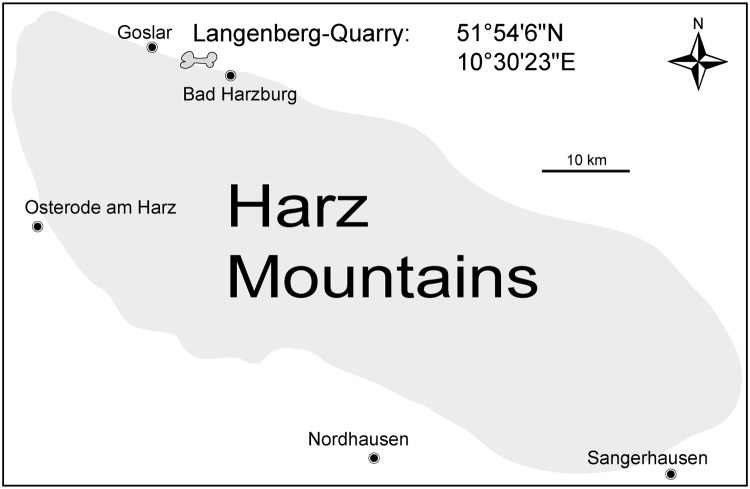
Location of the Langenberg locality in Germany.

**Fig 2:**
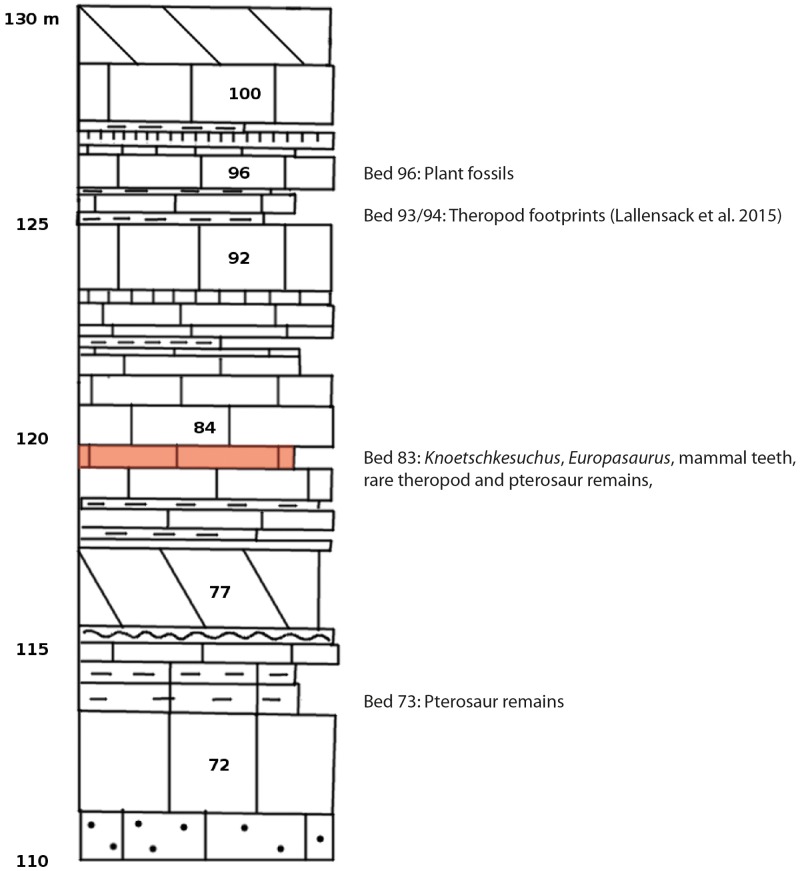
Geological section of the “Mittlerer Kimmeridge”. This figure is based on Fischer [29] and modified from Lallensack et al. [38]. Beds yielding terrestrial fossils are listed.

All described material has been found in the Langenberg Quarry at the northern rim of the Harz Mountains near the town of Goslar, Lower Saxony, northern Germany (Fig 1). The quarry exposes well-preserved sections of Upper Jurassic shallow marine strata [29–31]. The beds consist of impure carbonates which grade into marls and which are tilted to a near vertical, slightly overturned position. Quarrying by blasting proceeds along strike, exposing the beds only in cross-section and not along bedding planes. Throughout the sequence, changes in water depth and clear brackish influences are evident in the sediment composition and invertebrate fossil record, but except for very rare dinosaur tracks, evidence of subaerial exposure is missing [30–32]. Well dated biostratigraphically, the sediments in the quarry range from late Oxfordian to late Kimmeridgian in age [29–31] and belong to the Süntel Formation [33]. Most terrestrial vertebrate remains including the sauropod dinosaur *Europasaurus holgeri* [34–36] and all atoposaurid material described herein was found in bed 83 (Fig 2), and not in bed 93 as erroneously stated in several recent publications [34–36]. Bed 83 is a light grey-greenish marly limestone, which contains micritic intraclasts up to several cm in diameter. It has been assigned to the “Mittleres Kimmeridge”, a northwest-German equivalent to the lower part of the Upper Kimmeridgian of the international chronostratigraphic time scale [37, 38].

Paleogeographically, the Langenberg Quarry is situated in the Lower Saxony Basin, which covered most of northern Germany during the Jurassic and Early Cretaceous. The region was probably an archipelago that was surrounded by large paleo-islands [39], which were the source of the clastic components in the sediment.

#### Fossil vertebrates from the Langenberg Quarry

In addition to the teeth and skeletons of small non-marine atoposaurid crocodylians described herein [6–8], the Langenberg Quarry is well known as the type and only locality of the dwarfed sauropod *Europasaurus holgeri* [34–36]. Similar to the atoposaurid remains, *Europasaurus* is represented by abundant and exquisitely three-dimensionally preserved material. Other dinosaur material from the quarry includes isolated teeth of several different groups of theropod dinosaurs [40] as well as natural track casts of large theropods [32].

The beds 83 and 73 (Fig 2) also yielded other well-preserved terrestrial fossils such as conifer twigs and cones [5], the first Jurassic mammal teeth from Germany [5, 41], the three-dimensionally preserved articulated skeleton of a small pterosaur, which was described as the first dsungaripterid from the Kimmeridgian of Germany [42], and the associated remains of a partial skeleton of a paramacellodid lizard [43]. The diverse turtle material comprises cf. *Thalassemys*, *Plesiochelys*, and possibly a new taxon [44]. Microvertebrate remains from the Langenberg are dominated by a diverse fish assemblage represented mainly by isolated teeth of marine chondrichthyans and osteichthyans [45–47], but also include many reptilian teeth (Wings, pers. obs.).

#### Taphonomy

Almost all of the fossil material of terrestrial vertebrates—including all atoposaurid material described herein—was recovered after regular blasting operations in the quarry. Despite the large number of bones and teeth known from *Europasaurus* and other taxa, the density of terrestrial vertebrate fossils in bed 83 is scarce. Skeletal remains seem to have been accumulated in lenses or channels. The bone-bearing sections of bed 83 are typically 30–50 cm thick and also contain a large number of well-rounded micritic intraclasts in all bone-rich areas. The combination of bone material and the distinctive intraclasts is also important for recognizing blocks of this specific layer in the quarry heap after blasting.

**Fig 3:**
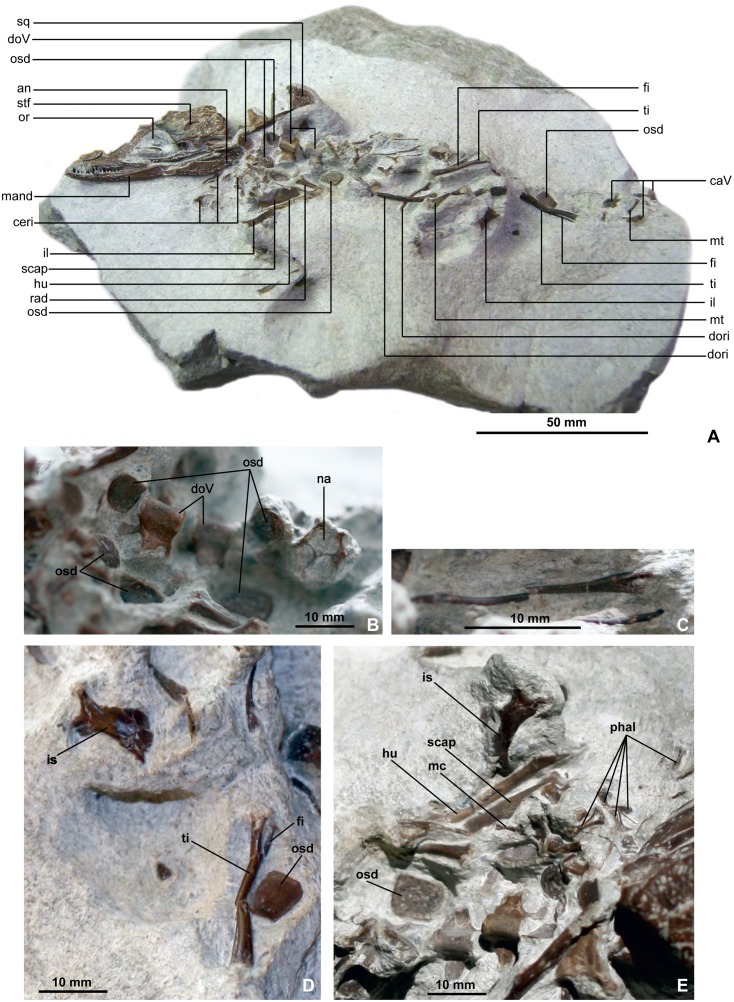
*Knoetschkesuchus langenbergensis* gen. nov. sp. nov, DFMMh/FV 200, skeleton of larger specimen. (A) Overview photograph of *Knoetschkesuchus langenbergensis* gen. nov. sp. nov., larger specimen DFMMh/FV 200 in dorsolateral aspect. (B-E) Details of postcranial bones from slab DFMMh/FV 200 of *Knoetschkesuchus langenbergensis* gen. nov. sp. nov. For anatomical abbreviations, see text.

Because the specimens were not found *in situ*, it remains possible, although very unlikely, that the finds come from another bed nearby. In any case, they can be certainly assigned to the lower part of the Upper Kimmeridgian. The vast majority of the delicate bones are very well preserved, indicating very limited transport. The onset of disarticulation of the carcasses is evident in DFMMh/FV 200 (Fig 3) and also to a smaller degree in DFMMh/FV 605, suggesting weak currents and an exposure time of several days before burial.

## Materials and methods

All described fossils were found in an Upper Jurassic (Upper Kimmeridgian) marine marly limestone, bed 83 after Fischer [29], at the Langenberg Quarry near Goslar, Lower Saxony in northwestern Germany. The material comprises the following collection numbers: DFMMh/FV 200, slab with one individual; DFMMh/FV 605, complete skull of a juvenile individual; DFMMh/FV 261, angular; DFMMh/FV 790.12, partial left dentary; DFMMh/FV 279, femur; DFMMh/FV 790.11, metatarsal; and DFMMh/FV 325, osteoderms, vertebra and partial ribs. The fossils are housed at the Dinosaurier-Freilichtmuseum Münchehagen/Verein zur Förderung der Niedersächsischen Paläontologie (e.V.) (catalogue numbers DFMMh/FV), in Rehburg-Loccum near Hannover in northern Germany (Dinopark Münchehagen, Alte Zollstraße 5, 31547 Rehburg-Loccum, Germany). The statutes of the Förderverein (supporting association) state that all of the DFMMh material will always be accessible to researchers; in case of the liquidation of the association it will imperatively be transferred to a public institution. Preparation of the specimens was carried out at the preparation lab of the Dinosaurier- Freilichtmuseum Münchehagen. Matrix surrounding the bones was removed using pneumatic micro-scribes (Micro Jacks by PaleoTools). Due to the small size of the specimens, most of the morphological studies of the specimens were carried out under a stereomicroscope (Leica WILD MZ8).

Micro-computer tomography scans were carried out at the Steinmann Institut at the University of Bonn with a v|tome|x phoenix|x-ray as well as at the Museum für Naturkunde Berlin by using a Phoenix|x-ray Nanotom tomography machine (GE Sensing and Inspection Technologies GmbH, Wunstorf, Germany). Slices were reconstructed using the datos|x-reconstruction software, version 1.5.0.22 64 bit (GE Sensing and Inspection Technologies GmbH, Phoenix|x-ray). The resulting volume with the highest resolution was segmented and analyzed with the softwares Avizo v.8.1 and MeshLab v.1.1.0.

No permits were required for the described study.

### Nomenclatural acts

The electronic edition of this article conforms to the requirements of the amended International Code of Zoological Nomenclature, and hence the new names contained herein are available under that Code from the electronic edition of this article. This published work and the nomenclatural acts it contains have been registered in ZooBank, the online registration system for the ICZN. The ZooBank LSIDs (Life Science Identifiers) can be resolved and the associated information viewed through any standard web browser by appending the LSID to the prefix “http://zoobank.org/”. The LSID for this publication is: urn:lsid:zoobank.org:pub: 9C5AB666-4217-4472-88D6-7261CEFA3F3E. The electronic edition of this work was published in a journal with an ISSN, and has been archived and is available from the following digital repositories: PubMed Central, LOCKSS.

## Systematic palaeontology

CROCODYLOMORPHA Walker, 1968[48]

CROCODYLIFORMES Hay, 1930 [50][49]

MESOEUCROCODYLIA Whetstone and Whybrow, 1983 [50]

NEOSUCHIA Benton and Clark, 1988 [28]

EUSUCHIA Huxley, 1875 [51]]

ATOPOSAURIDAE Gervais, 1971[9]

*Knoetschkesuchus* gen. nov.

### Derivation of name

The genus name is a combination of the family name of Nils Knötschke, and suchus (from Greek: souchos), meaning crocodile. The name is erected in honor of Nils Knötschke (Dinosaurier-Freilichtmuseum Münchehagen), who collected, prepared, and curated a large number of fossil specimens from the Langenberg Quarry, contributing significantly to the conservation of its natural history heritage.

### Type species

*Knoetschkesuchus langenbergensis* sp. nov.

### Diagnosis

Among atoposaurids, *Knoetschkesuchus* is diagnosed by the following unique combination of features and autapomorphies (marked by asterisk) 1)*Dentition of maxilla and dentary comprises two morphotypes, including a) conical and pseudocaniniform teeth with weak labial and lingual striae, and b) rounded conical, “lanceolate” teeth with lingual fan-shaped striations; 2) Secondary choanae are slit-like foramina on the surface of the pterygoid within a narrow groove; 3)*Minimum space between the supratemporal foramina comprises one third of the total width of the cranial table; 4)*Minimum width of the frontal between the orbits comprises one third of the total skull width; 5) lacrimonasal contact absent or only with a minute rostral tip of the lacrimal; 6) antorbital fenestra present; 7) external mandibular fenestra present.

### Other referable species

*Theriosuchus guimarotae* Schwarz & Salisbury 2005 from the Upper Jurassic Guimarota strata in Portugal.

*Knoetschkesuchus langenbergensis* sp. nov.

urn:lsid:zoobank.org:act:7F1432BA-9C3D-4EAD-872F-40F55438DCCD

### Derivation of name

Referring to the Langenberg Quarry, the type locality for this taxon.

### Holotype

DFMMh/FV 200, slab with one individual comprising partial skull and mandible, thoracic, lumbar and caudal vertebrae, scapula, humerus, ischium, metatarsals, manual and pedal phalanges and osteoderms.

### Referred specimens

DFMMh/FV 605, complete skull of a juvenile individual;DFMMh/FV 261, angular; DFMMh/FV 790.12, partial left dentary; DFMMh/FV 279, femur; DFMMh/FV 790.11, metatarsal; DFMMh/FV 325, osteoderms, vertebra and partial ribs.

### Type locality and horizon

Langenberg Quarry near Goslar, Lower Saxony (northwestern Germany); Lower Saxon Basin, Süntel Formation (Upper Jurassic, Upper Kimmeridigan), bed 83 after Fischer [29].

### Diagnosis

Secondary choanae positioned only in the rostralmost part of the pterygoid wings, distinct lateral crest on the descending postorbital process, squamosal laterally overlapping the postorbital in its rostral extent, rostromedially directed premaxillomaxillary suture, mandibular symphysis extending to the 8^th^ dentary tooth, low angular and low caudal mandibular ramus.

*Knoetschkesuchus guimarotae* (Schwarz & Salisbury 2005), **new combination**

### Material examined

IPFUB Gui Croc 7308, partial skull and mandible, part of an isolated surangular, a second sacral vertebra and two partial osteoderms; Cranial and mandibular remains: IPFUB Gui Croc 7309, 7403–1, 7709 (partial skulls); and all other specimens listed in Schwarz & Salisbury 2005.

### Referred specimens

See referred material in Schwarz & Salisbury 2005.

### Type locality and horizon

Coal mine of Guimarota, a suburb of Leiria in north-western Portugal; lower (“Fundschichten”) and upper (“Ruafolge”) lignite coal layer of the “Guimarota-Strata”, within the Alcobaça Formation, Upper Jurassic (Kimmeridgian).

### Diagnosis

According to Schwarz & Salisbury 2005, *K*. *guimarotae* possesses the following unique combination of features: lateral surface of squamosal beveled ventrally, with a distinct lateral notch rostrally; caudolateral corner of the squamosal forms a rounded, caudally projecting process with sculpture pitting on its dorsal surface that is similar to that on the rest of the cranial table; premaxillomaxillary suture aligned caudomedially in dorsal aspect; minimum space between supratemporal foramina comprises one third of the total width of the cranial table; dentition that comprises only pseudocaniniform and lanceolate-shaped teeth, all with mesial and distal carinae; minimum width of the frontal between the orbits comprises one third of the maximum width of the skull at the orbits; external mandibular fenestra present; all vertebral bodies amphicoelous.

### Comments

According to the genus diagnosis of *Knoetschkesuchus* gen. nov. as given in this paper, the minimum space of the supratemporal foramina and the interorbital space, the dentition with two tooth morphs, and the presence of an external mandibular fenestra are diagnostic on a genus level. The occurence of amphicoelous vertebrae is not an autapomorphic feature and should be omitted. *Knoetschkesuchus guimarotae* (new combination) is distinguished from *K*. *langenbergensis* by a caudomedially directed premaxillo-maxillary suture, a more elongate prefrontal, a rectangular parietal, absence of a distinct lateral crest on the descending postorbital process, a squamosal medially overlapping the postorbital in its rostral extent, secondary choanae positioned in the rostral half of the pterygoid wing, a mandibular symphysis extending to the 6^th^ dentary tooth, and a relatively higher angular and caudal mandibular ramus.

## Description

### Preservation

All specimens are three-dimensionally preserved with little or no distortion. Several bones have been removed from the matrix, but their foramina, other openings and fractures are still filled with carbonate. The holotype skull is prepared from dorsally and both lateral sides, but ventrally embedded in matrix (Fig 3A). A large crack dorsally at the cranial table has been filled with glue. The juvenile skull DFMMh/FV 605 has been prepared completely. Teeth are preserved *in situ* on both skulls and the isolated dentary (DFMMh/FV 790.12). Both skulls were slightly crushed dorsally during diagenesis, and in particular DFMMh/FV 200 shows medially tilted dermal bones because of the distortion of the skull. Investigations by μCT of the juvenile skull DFMMh/FV 605 supplement the description. Most vertebrae lack their neural spines. None of the bones shows signs of abrasion or weathering, but several bones have fractures along their surfaces. Completeness and preservation of small cranial elements and other bones indicate a short distance transport before burial. Details on preservation of the skeletal regions are given together with the description.

### Skull

#### General description

**Fig 4:**
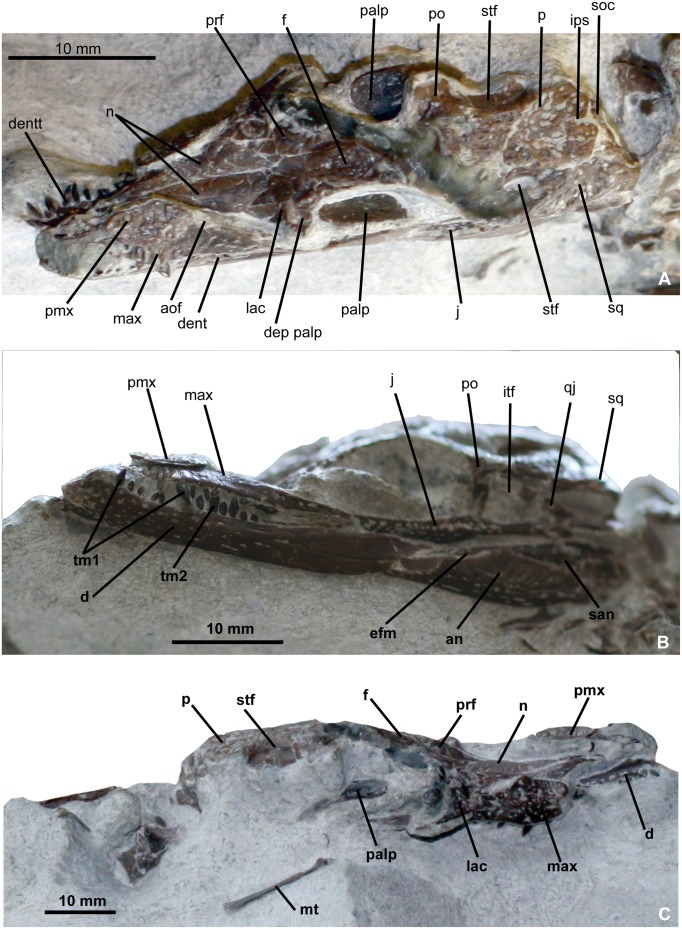
Skull of *Knoetschkesuchus langenbergensis* gen. nov. sp. nov, DFMMh/FV 200, larger specimen. Skull photographs (A) in dorsal aspect, (B) in left lateral aspect, and (C) in right dorsolateral aspect. For anatomical abbreviations, see text.

**Fig 5:**
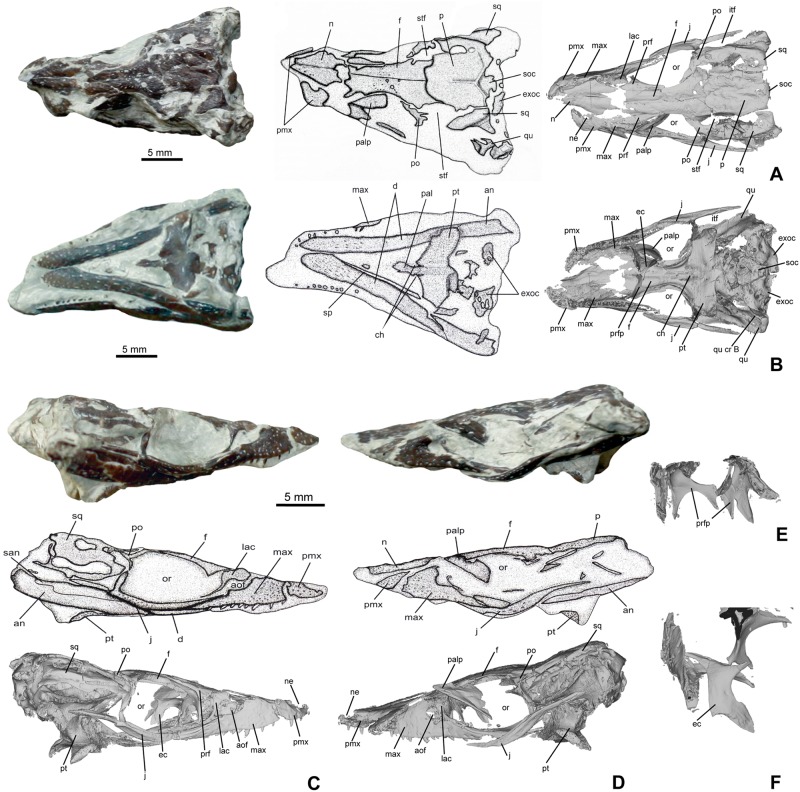
Skull of *Knoetschkesuchus langenbergensis* gen. nov. sp. nov, DFMMh/FV 605, juvenile specimen. The figure shows photographs of the original specimen, interpretative drawing with labels, and reconstructed 3D data as derived from MeshLab (FOV: Ortho; only skull elements originally visible and important in the respective view are shown). (A) Dorsal aspect. (B) Ventral aspect. (C) Right lateral aspect. (D) Left lateral aspect. (E) Caudal view showing isolated prefrontals with prefrontal pillars as 3D reconstruction. (F) Prefrontals and left ectopterygoid in caudal aspect, 3D reconstruction. For anatomical abbreviations, see text. 3D images are not to scale.

The skull is brevirostrine, with a rostral length/total skull length ratio of 0.47 in DFMMh/FV 200, and of 0.37 in DFMMh/FV 605. In dorsal aspect, there is a gradual expansion of the skull around the orbital region, the rostrum tapers continuously in rostral direction (Figs 4A and 5A). In particular DFMMh/FV 605 has a narrow and rounded nearly triangular outline of the rostrum (Fig 5A). The margin of the rostrum undulates laterally, producing two lateroventral convexities positioned at mid-length of the premaxilla and at the fourth and fifth maxillary tooth. An indentation is formed at the premaxillo-maxillary suture. In DFMMh/FV 200, the orbit comprises 28% of the total skull length and in DFMMh/FV 605 33%. In lateral aspect, the skull is broad oreinirostral, which becomes particulary evident at the vertically oriented suture between the nasal and the maxilla (Fig 5C and 5D) that indicates a more or less vertical orientation of the maxilla. The cranial table has a rounded rectangular shape, but widens slightly in caudal direction. The supratemporal foramina occupy not more than one fourth of the area of the cranial table. The lateral margin of the cranial table is convex, and the caudal margin is concave with a small median incision at the parietal in DFMMh/FV 200. A much stronger median incision at the caudal parietal margin is visible in DFMMh/FV 605, exposing the supraoccipital on the skull roof in dorsal aspect (Fig 5A). The caudolateral edges of the cranial table, formed by the squamosal, are caudolaterally drawn out. A pronounced sculpturing of regular circular pits is present on the cranial table, jugal, rostral part of maxilla and premaxilla. The caudal parts of nasal, palpebral and lacrimal are slightly sculptured with rounded pits, whereas the rostral part of nasal and frontal expose weak longitudinal ridges. In comparison with DFMMh/FV 200 (Fig 4A), DFMMh/FV 605 (Fig 5A) possesses only random and more shallow sculpturing of the dermal bones.

#### External naris

On both specimens (DFMMh/FV 200 and DFMMh/FV 605), the indentation of the medial margin of the premaxilla indicates that the external naris was positioned in the rostral half of the bone (reaching the rostral skull margin) and faced dorsally (Figs 4A, 5A and 5C). The nasopremaxillary suture, well exposed in DFMMh/FV 200 suggests together with the medial premaxillary margin that a median thin spine of the nasals reached the caudal margin of the external naris. It remains unclear if there is a complete separation of left and right naris by a median bone bar of the nasal.

#### Antorbital foramen

The right maxilla of DFMMh/FV 200 shows an indentation of the caudomedial margin associated with a rostrally positioned depression on the maxilla that is interpreted as the margin of a small antorbital foramen (Fig 4A). The antorbital foramen of DFMMh/FV 200 is proportionally only slightly reduced in size in comparison to DFMMh/FV 605. The antorbital foramen of DFMMh/FV 605 is located rostral to the orbit and is rostrally, rostromedially and ventrally restricted by the maxilla (Fig 5C and 5D). The caudal and caudomedial margin are bounded by the lacrimal. The antorbital foramen is longitudinally oval in outline and a slight antorbital fossa is developed in the maxilla of DFMMh/FV 605. The length of the antorbital foramen is 9% that of the orbit (Fig 5C).

#### Orbit

The large, longitudinally oval orbit is 54% longer than it is wide in DFMMh/FV 200 (Fig 4B) and 27% longer than it is wide in DFMMh/FV 605 (Fig 5A and 5C). Rostrolaterally, the orbit is bounded by the lacrimal, rostromedially by the prefrontal. Its lateral and caudolateral margins are formed by the jugal, which excludes the maxilla from the orbit. The xpillarlike postorbital bar separates the orbit caudally from the infratemporal fenestra. The caudomedial margin of the orbit is restricted by the frontal, which is not elevated from the orbital margin, and the caudal margin of the orbit by the postorbital.

#### Supratemporal foramen

In DFMMh/FV 200, the supratemporal foramen is irregular in shape, with a rounded rectangular rostromedial and rostrolateral margin, and a narrow and rounded caudal half (Fig 4A). It is 42% longer than wide. In DFMMh/FV 605, the supratemporal foramen is narrow oval-shaped and 54% longer than wide (Fig 5A). In DFMMh/FV 200, the supratemporal foramen is bounded by the postorbital along the rostral and rostrolateral margin (Fig 4A). In contrast, in DFMMh/FV 605, the rostromedial corner of the supratemporal foramen is formed by the frontal (see also “Ontogeny”) (Fig 5A). The caudomedial margin of the supratemporal foramen is bounded by the parietal in both specimens, the caudal and caudolateral margins are formed exclusively by the squamosal. The wall of the supratemporal fossa is formed rostromedially by the frontal and caudomedially by the parietal. The squamosal seems to contribute to most of the lateral part of the supratemporal fossa, but because of the poor preservation of this region in both specimens, it remains unclear if there is also a contribution of the quadrate.

#### Infratemporal fenestra

The infratemporal fenestra of both skulls is roughly rounded trapezoidal in outline, laterally longer than medially and tapers caudolaterally to a tip. It is 1.5 times as long as it is wide. The fenestra is bounded rostromedially by the postorbital, rostrally by the postorbital bar and laterally by the jugal. Caudomedially, the infratemporal fenestra is bounded by the squamosal and caudolaterally by the quadratojugal.

#### Secondary choanae

**Fig 6:**
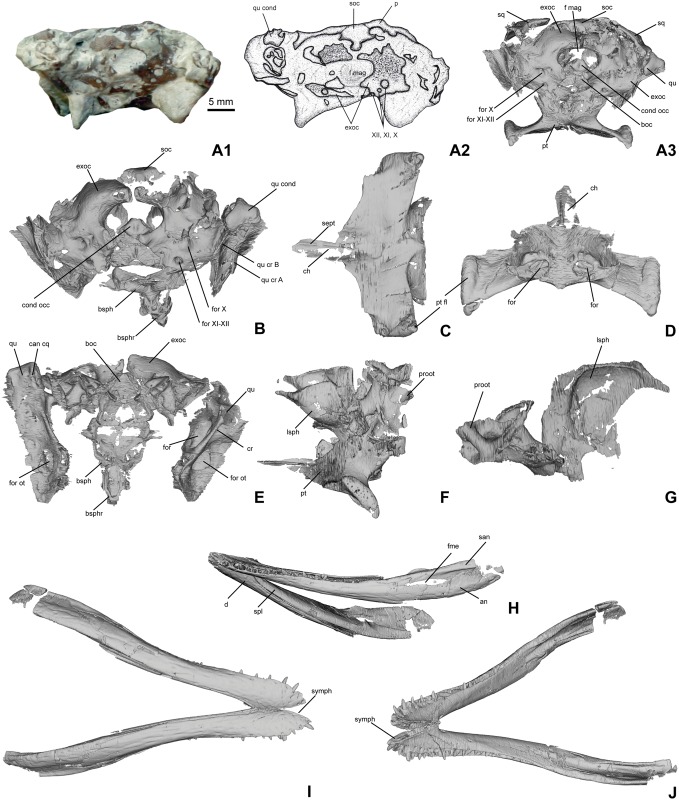
DFMMh/FV 605, skull and mandible of *Knoetschkesuchus langenbergensis* gen. nov. sp. nov., juvenile specimen. (A) Occipital view of skull, (A1) photograph of the original specimen on the left side, (A2) interpretative drawing with labels in the middle, and (A3) reconstructed 3D data as derived from MeshLab (FOV: Ortho; only skull elements originally visible and important in the respective view are shown). (B) Caudoventral aspect of skull with basisphenoid exposed. (C) Pterygoid in ventral aspect. (D) Pterygoid in dorsal aspect. (E) Dorsal aspect of ventral skull elements, featuring quadrates, exoccipitals, basioccipital and basisphenoid. (F) Left lateral aspect of braincase wall including pterygoid, prooticum and laterosphenoid. (G) Medial aspect of left laterosphenoid and prooticum. (H) Mandible in left ventrolateral aspect. (I) Mandible in ventral aspect. (J) Mandible in dorsal aspect. For anatomical abbreviations, see text. 3D images are not to scale.

The paired secondary choanae are partly preserved in DFMMh/FV 605 (Figs 5B and 6C). The openings of the secondary choanae are positioned within the through-like depressions (choanal groove) at the rostral process of the pterygoid and are medially separated by a rounded crest. The secondary choanae are longitudinal slitlike openings placed with its caudal half in the rostral narrow process of the pterygoid. The rostral edge of the choanae is situated between the suborbital fenestrae in the palatines. Only a small, caudalmost part is positioned in the pterygoid wings. Caudal to the secondary choanae, the pterygoid surface is slightly elevated, but there is no distinct vertically high rim present.

#### Premaxilla

DFMMh/FV 200 preserves the left premaxilla with one tooth *in situ* but the rostral-most portion of the bone is missing (Fig 4A). In DFMMh/FV 605, the left premaxilla is complete but without teeth and disarticulated from the rostrum, whereas only a small part of the right premaxilla is exposed (Fig 5C and 5D). The premaxilla is approximately 1.5 times wider than long. Its lateral margin forms a convexity. Its medial margin is concave and thus provides space for the external naris. The premaxilla contacts the maxilla caudolaterally with a slightly serrated rostromedially extending suture and is medially connected to the nasal by a longitudinally straight suture, thus it participates only little in the internarial bar, if at all. The 3D reconstructions revealed that the suture between premaxilla and maxilla in ventral aspect is jagged and rostromedially directed (Fig 5C and 5D).

#### Maxilla

The maxilla is medially bent along its long-axis. The maxilla is widest in its rostral fourth, bifurcating then into a short medial and a long lateral process. The rostral margin of the maxilla is slightly rostromedially oriented and shares a serrated suture with the premaxilla. The lateral margin of the maxilla is convex in its rostral third with a maximum curvature at the fourth maxillary tooth. Caudally to this convexity it becomes slightly concave, then straight. The medial maxillary margin, including the medial process, contacts the nasal along a sagittally aligned suture. Caudal to the nasal, the medial process of the maxilla roofs the antorbital foramen and contacts the lacrimal. A dorsal furrow at the rod-like part of the maxillary process represents the contact area to the lateral part of the lacrimal, which attaches dorsally to the maxilla (Figs 4C and 5C). Only in DFMMh/FV 605, the length of the maxillolacrimal suture is visible over approximately four maxillary alveoli and the caudal contact to the jugal (Fig 5C). The lateral process of the maxilla preserves its maximum rod-like process that thins out far caudally. Directly caudal to the lacrimal, the maxilla underlaps the jugal, which attaches dorsally on the very thin caudalmost part of the lateral maxillary process with a smooth suture. In ventral aspect, the posterior palatal branches of the maxillae meet entirely rostral to the palatines (Fig 5B). The rostralmost part of the medial suture of the ventral maxilla to the palatine lies shortly rostrally to the suborbital fenestra.

#### Nasal

The nasals are a wedge-like pair of bones, forming the medial third of the rostrum (Figs 4A and 5A). The elongate element is narrowest rostrally and continues between the premaxillae to send a medial spine into the external naris. Caudally, the nasal widens to reach at the contact with the prefrontal five times of its rostral width at the contact with the prefrontal. Caudally to this contact, the nasal tapers again and wedges out between the lateral prefrontal and the medial frontal level with the rostral orbital margin. The contact between the left and right nasals is a straight median suture. The lateral suture to the maxilla is restricted to the cranial half of the nasal bone and directed vertically (Fig 4A).

#### Lacrimal

The lacrimal of DFMMh/FV 200 possesses in dorsal and lateral aspect a massive rounded squarish body from which a delicate process descends caudolaterally (Fig 4A). The rostral and caudal margins of the lacrimal are concave and mark the boundaries of the antorbital fenestra and the orbit, respectively. The medial margin of the lacrimal is slightly wavy and contacts the maxilla with its rostral tip and the prefrontal with its medial margin. The lacrimal is nearly completely excluded from a contact with the nasal by both of these elements, and touches the nasal only with a small rostromedial tip dorsal to the antorbital foramen. Laterally, the lacrimal attaches to the maxillary process and contacts the jugal in the rostral third of the orbita (Fig 4A). In contrast, the lacrimal of DFMMh/FV 605 is more delicate than in DFMMh/FV 200, having a more robust dorsal part from which a delicate caudoventrally bent process descends (Fig 5C and 5D). The delicate caudolateral process of the lacrimal is enlarged and also forms the boundary between antorbital fenestra and orbit (Fig 5C).

#### Prefrontal

The prefrontal is nearly triangular in outline in dorsal view (Fig 4A and 4C). Having a nearly straight medial margin, it tapers rostrally to a tip and forms a narrow expansion laterally (Fig 5A and 5C). Caudally, the prefrontal forms another narrow expansion that represents the rostral third of the dorsal orbital margin. The medial margin of the prefrontal is bounded in its rostral half by the nasal and in its caudal half by the frontal. Craniolaterally, the prefrontal is restricted by the lacrimal, caudally it contributes to the orbital margin. The rostral margin of the orbit bears in its rostromedial half a broadened depression that comprises equal parts of lacrimal laterally and prefrontal medially (Fig 4A). This depression represents the attachment area for the palpebral. The prefrontal pillar is not exposed in DFMMh/FV 200, but preserved as a narrow and flat ventral process in DFMMh/FV 605 (Fig 5E). The prefrontal pillar is positioned in the rostromedial edge of the orbit and decends from the prefrontal body ventromedially to contact the palate (Fig 5B and 5E). The prefrontal pillar is dorsally and ventrally expanded, with a medial constriction.

#### Palpebral

A left and a right palpebral are preserved in the orbits of DFMMh/FV 200 (Fig 4A and 4C), and a left palpebral is preserved in DFMMh/FV 605 (Fig 5A and 5D). The palpebrals are roughly drop-shaped in outline with a convex dorsal margin that mirrors the concave medial orbital margin and a narrow rostral end. The lateral palpebral margin is straight and aligned rostrocaudally. The external surface of the palpebral is slightly vaulted and weakly sculptured. On its internal smooth surface, the palpebral is slighty concave in its caudal two thirds. In its rostral third, the palpebral forms an oval articular surface on the internal side that probably was positioned in the depression of the rostral orbital margin.

#### Frontal

**Fig 7:**
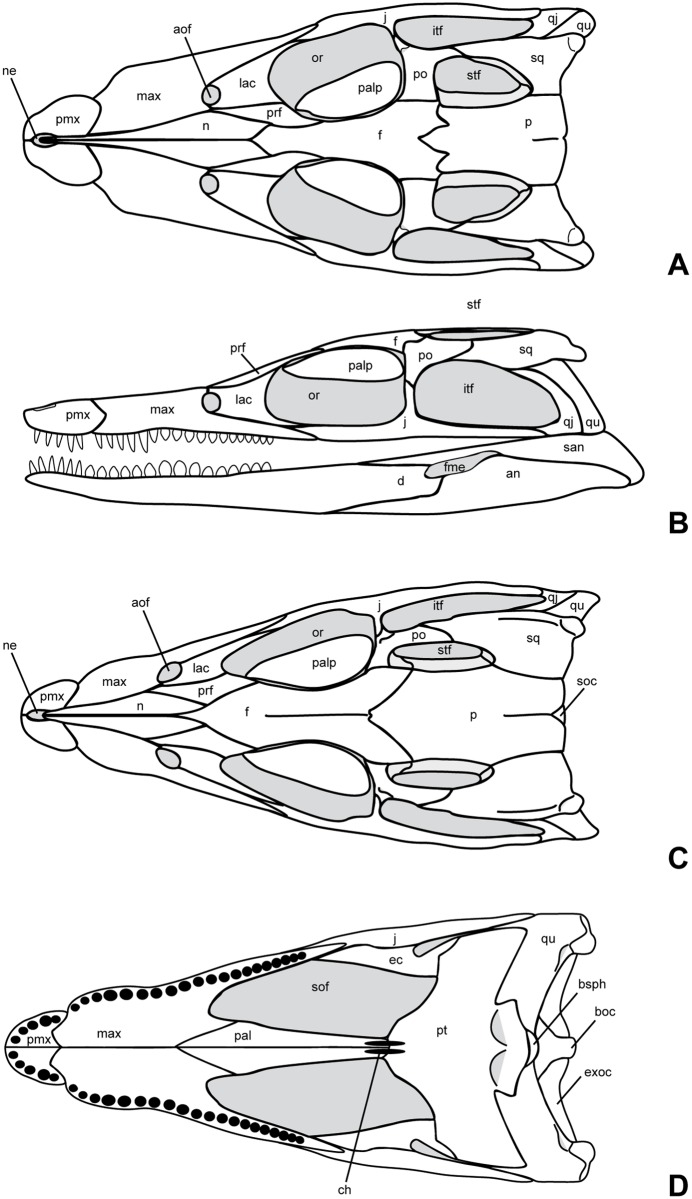
Reconstructions of the skull and mandible of *Knoetschkesuchus langenbergensis* gen. nov. sp. nov. Reconstruction of larger skull based on specimen DFMMh/FV 200, missing information supplemented with information from skull DFMMh/FV 605. (A) Dorsal aspect. (B) Left lateral aspect with mandible. Reconstruction of juvenile specimen DFMMh/FV 605, based on skull morphology and 3D data obtained in MeshLab. (C) Dorsal aspect. (D) Ventral aspect. For anatomical abbreviations, see text. Not to scale.

In DFMMh/FV 200, only the rostral and interorbital part as well as the caudalmost part of the frontal are preserved (Fig 4A). The frontal contributes both to the interorbital bone and the rostral part of the cranial table. From its rostral, wedge-like process, the bone widens moderately in caudal direction to reach twice its rostral width at the orbit. The frontal maintains its width between the orbits, being roughly as wide as the nasals, and widens continuously on the skull roof (Figs 4A, 4C and 7A). From the direct contact between postorbital and parietal here it is visible that the frontal is mostly excluded from the supratemporal foramen (Fig 4A), and the serrated frontoparietal suture lies at the rostral end of the supratemporal foramen. The frontal bone is complete in DFMMh/FV 605 (Fig 5A). The frontal contacts contacts the postorbital laterally at the skull roof. The rostral, midorbital part of the frontal is somehow inflated in comparison with DFMMh/FV 200. The medial frontal wedge is more acute and more narrow than in DFMMh/FV 200 (Fig 7). Caudally, the suture to the parietal is jagged and rostrally convex, so that the caudal margin of the frontal becomes deeply concave. The frontoparietal contact is positioned in the rostral fourth of the length of the supratemporal foramen. In DFMMh/FV 605, the frontal bears a median blunt ridge that denotes the median suture of the once unfused frontals. The caudal third of the frontal is vaulted medially and slightly depressed laterally. In ventral aspect (Fig 5B), the orbital margin of the frontal is recurved, so that the ventral surface of the frontal forms a shallow trough. Caudal to the orbit, the frontal forms a lateral projection, which is unsculptured and slightly depressed to enter the supratemporal fossa.

#### Parietal

The parietal is roughly rounded rectangular in outline, but its width slightly increases caudally (Fig 4A). The lateral parietal margin forms in its cranial half the medial margin of the supratemporal foramen, and in its caudal half is bounded by the squamosal so that it does not contribute to the caudal margin of the supratemporal foramen. The parietosquamosal suture is longitudinally straight. In DFMMh/ 200, the caudal margin of the parietal is medially slightly notched and forms the medial third of the caudal margin of the cranial table, excluding the supraoccipital from the latter (Fig 4A). In DFMMh/FV 605, the caudal margin bears a deep incision that exposes the supraoccipital dorsally (Fig 5A). The parietal is incompletely fused and bears in its caudal two thirds a thin median longitudinal suture, but its dorsal surface is even (Fig 5A).

#### Squamosal

The squamosal is approximately four times wider caudally than it is rostrally (Figs 4A and 5C). Rostrolaterally, the squamosal contacts the postorbital in the rostral third of the length of the supratemporal foramen, where it overlaps the postorbital laterally in its rostral extent. Its lateral margin is convex. In its medial third, the lateral squamosal margin is beveled to form an unsculptured lateral bordure set off from the cranial table by a marginal crest. The unsculptured beveled lateral area in DFMMh/FV 200 is small and extends from the caudolateral third of the dorsal squamosal area to the caudal squamosal corner (Fig 4A and 4B). The beveled portion is similarly long in DFMMh/FV 605 as in DFMMh/FV 200, but deeper, hanging into the infratemporal foramen (Fig 5C and 5D). The caudal margin of the squamosal is medially concave, but drawn out into a caudolaterally directed lobe at its lateral corner. This lobe is one fourth longer and more narrow in DFMMh/FV 605 (Fig 5A) than in DFMMh/FV 200 (Fig 4A). Similar to the beveled lateral margin, the caudolateral process of the squamosal is unsculptured and abated from the cranial table. The dorsal margin is straight in occipital aspect and the ventral contact of the squamosal to the exoccipital is visible in parts. Contact areas to the quadrate are in both specimens either destroyed or obscured.

#### Postorbital

Details of postorbital anatomy are visibly mostly in DFMMh/FV 605 (Fig 5A). The postorbital lies dorsal and medial to the jugal. In dorsal aspect, the postorbital divides into a medially directed part contacting the frontal, and a caudally directed part that contacts the squamosal, both having an orientation in a wide angle of approximately 130° to each other (Fig 5A). The caudally directed part of the postorbital is broader in DFMMh/FV 200 than in DFMMh/FV 605 and sculptured (Fig 4A and 4B), thus forming a broad dorsal surface between the orbit and the supratemporal fossa. In contrast, the postorbital bar between orbit and supratemporal fossa is very narrow in DFMMh/FV 605 (Fig 5A), being only half as wide as in DFMMh/FV 200. The junction between these two parts forms a gentle curve in the rostrolateral corner of the cranial table. Laterally on the corner is a slightly ventrally tilted and medially depressed area from which a constricted and smooth process descends and unites at mid-height of the postorbital pillar with an ascending process of the jugal (Figs 4B, 5A and 5C). Together, the processes of the jugal and the postorbital form the thin, pillarlike postorbital bar. The postorbital part of this bar is a laterally directed crest, and contains at its dorsal end a small vascular foramen (Fig 5C and 5D). In medial aspect, the postorbital medial margin, which forms the rostrolateral wall of the supratemporal fenestra, is recurved ventrally and does not contribute to the inner wall of the supratemporal fossa.

#### Jugal

The jugal is a rostrocaudally elongate bone that contributes both to the orbit and the infratemporal fenestra (Figs 4B, 5C and 5D). At mid-length, it forms a rod shaped caudodorsally ascending jugal process that constitutes the ventral half of the postorbital bar. This process is displaced medially from the lateral surface of the jugal and separated from the latter by a ridge. The dorsal margin of the jugal is convex at the rostral end of the postorbital bar, and rostrally and caudally to it weakly concave. The jugal body is rostrally to the postorbital bar only slightly dorsoventrally higher than caudally, where it forms a slender rodlike process (Fig 5A). The most rostral part of the jugal becomes very slender and wedges out between the underlying maxilla and the caudoventral process of the lacrimal (Fig 5C and 5D).

#### Quadratojugal

The quadratojugal is preserved only in DFMMh/FV 200 (Fig 4B). In lateral aspect, the quadratojugal is an elongated, rectangular bone that extends from rostromedially to caudolaterally. Medially, it contacts rostrally the squamosal with a narrow process, and caudally with a straight and tight suture the medially positioned quadrate. The quadratojugal forms the caudolateral corner of the infratemporal fenestra (Fig 7A). Lateroventrally, the quadratojugal attaches to the jugal, and forms together with the quadrate the caudolateral corner of the skull.

#### Quadrate

In DFMMh/FV 200, a strip of the left quadrate is visible laterally, underlying the squamosal and being laterally in contact with the quadratojugal (Fig 4A). In DFMMh/FV 605, the articular condyle of the left quadrate is visible on the specimen directly, but the lateralmost extent is obscured by sediment.

μCT data of DFMMh/FV 605 depict both quadrates, with the right quadrate being completely preserved and the left one being fragmentary and lacking its condyle (Figs 5B and 6E). In occipital aspect, the quadrate condyle, which is entirely formed by the quadrate itself, possesses a kidney-shaped outline from which a rounded prong ascends dorsally. There is no good separation into a medial and a lateral hemicondyle and no intercondylar groove is visible. The ventral margin of the condyle is concave, and the medial part of the condyle is enlarged in comparison with the lateral part (Figs 5B and 6B).

In dorsal aspect, the quadrate is elongate and rounded rectangular in shape. It is divided into a large dorsolateral part and a medially inclined medial part that is half the size of the lateral part, both separated from each other by a rounded longitudinal bulging crest (Fig 6E). The dorsolateral part ends laterally with a serrated margin that forms a suture to the quadratojugal. The dorsolateral surface is caudally flat, but medially depressed in its rostral half. In the center of the rostral half lies a large, rostrocaudally rounded oval shaped otic foramen. The open (i.e., unroofed) cranioquadrate canal is visible in dorsal view, extending from the dorsal margin of the medial quadrate condyle rostrolaterally (Fig 6E). Squamosal and exoccipital do not contribute to the cranioquadrate canal.

Medial to the cranioquadrate canal, the surface of the quadrate bends ventromedially. The medial margin curves from the quadrate condyle in medial direction and contacts the exoccipital with a serrated, straight medially directed suture. The caudal edge of the quadrate is narrow and strongly concave dorsal and lateral to the otoccipital contact. Dorsal to this curved margin, the quadrate is roofed by a crest that overhangs the foramen vagii (Fig 6A3 and 6B). The foramen leads into a canal in rostral direction. Rostrodorsally to the crest, the ventromedially directed part of the quadrate bears another large and rounded oval foramen (Fig 6A3 and 6B). This foramen opens into a larger bipartite passage for the carotid artery and the cranial nerves IX–XI. The quadrate contacts the pterygoid ventrally, and the prootic and the laterosphenoid of the braincase rostrodorsally. These bones, however, are not preserved in articulation so that the contact cannot be determined more precisely. The ventral quadrate surface bears a well developed and high crest B [52], that extends from the lateral part of the quadrate condyle rostromedially, and a more rounded crest A, positioned relatively central at the quadrate (Fig 6E).

#### Pterygoid

The pterygoid is well preserved in DFMMh/FV 605, where it is exposed in ventral aspect on the ventral face of the skull (Figs 5B, 6C and 6D). The pterygoid wings are well developed and straight laterally directed. The pterygoid wings make the bone twice as wide as it is long. The pterygoid wings possess in ventral view a slightly concave rostral and a slightly convex caudal margin. The rather straight lateral margin extends obliquely from craniomedially to caudolaterally. A distinct pterygoid flange is visible as a rugose and thickened area at the margin of the pterygoid wing (Fig 6C and 6D), rostral to which the pterygoid wing is laterally depressed. The pterygoid body is slightly depressed on its ventral face, but it bears a faint median ridge. The pterygoid forms a median process that tapers rostrally. Left and right to the median ridge, a small, distinct longitudinally oval depression is developed that continues rostrally on each side of the narrow rostral process like a furrow (choanal groove) (Fig 6C). No distinct contact to the rostrally adjacent palatines is visible. Caudally, the pterygoid is in contact with the basisphenoid, which itself is only preserved in small bone fragments. The dorsal part of the pterygoid extends dorsally to contact the laterosphenoid and form the ventrolateral edge of the trigeminal foramen (Fig 6F). The suture to the quadrate and the laterosphenoid is clearly visible (Fig 6F).

#### Ectopterygoid

The only preserved left ectopterygoid of DFMMh/FV 605 is visible in the μCT reconstruction (Fig 5F), but preserved slightly disarticulated. The ectopterygoid has a constricted and slightly twisted body with a nearly straight rostromedial margin that forms the boundary of the suborbital fenestra, and a strongly concave caudolateral margin. The rostrolateral margin is slightly concave and rough, and attaches to the maxilla and the jugal. Because of the isolated position, the length of this contact and the contact to the maxilla cannot be determined, but the size of the ectopterygoid in relation to the skull size suggests that the ectopterygoid reached caudally at least to the dorsal process of the jugal. Caudally, the ectopterygoid is drawn out into a tongue-shaped and slightly striated and rugose medial process (Fig 5F). This process is attached to the rostrolateral margin of the pterygoid wing (Fig 7D).

#### Supraoccipital

The supraoccipital is subtrapezoidal in outline. On the occiput, it is ventrally bounded by the exoccipital (Fig 6A). It is separated from the cranial table by the parietal in DFMMh/FV 200, however, in DFMMh/FV 605, the supraoccipital is dorsally expanded and forms the medial third of the caudal margin of the cranial table (Figs 5a, 6A and 7C). In occipital view, the supraoccipital bears a rounded median crest and laterally to that a circular depression.

#### Exoccipital

DFMMh/FV 200 bears only fragments of the exoccipital, but this element is better preserved in DFMMh/FV 605 and could be depicted in the 3D reconstruction (Figs 5B and 6A). Left and right exoccipital are separated from each other in DFMMh/FV 605, and the paroccipital process is preserved only partially. The exoccipital bears a bone texture of weak grooves and pits, which is either diagenetic or a sign of poor ossification due to the ontogenetically young age of DFMMh/FV 605. The lateral half of the dorsal margin of the exoccipital is bounded by the squamosal. Medially, the exoccipital contacts the supraoccipital. The exoccipital forms the dorsal, lateral and lateroventral margins of the foramen magnum and excludes the supraoccipital from the latter. The ventral contact to the basioccipital is a broad and straight suture (Fig 6A). Lateroventrally to the foramen magnum, the exoccipital bears three circular foramina. The largest foramen lies at the exoccipital level to the dorsal margin of the foramen magnum and is interpreted as foramen for cranial nerve X and XI (foramen vagi) (Fig 6A and 6B). The foramen vagi is enlarged and internally bipartite to create a separate passage of cranial nerve IX medial to cranial nerves X and XI. A small foramen dorsomedial to the foramen vagi probably represents the exit of cranial nerve XII, whereas another, circular foramen ventral to the foramen vagi is interpreted as foramen caroticum posterior (Fig 6B).

#### Basioccipital and basisphenoid

The basioccipital and basisphenoid are preserved in DFMMh/FV 605, where they have been segmented from the 3D data (Fig 6A and 6B). Both are preserved adjacent to each other, but not in original articulation. The basioccipital is preserved with the occipital condyle, which is rounded trapezoidal in caudal aspect and slightly wider than high. Ventrally to the condyle, the basioccipital expands and the occipital surface bears in its ventral half a median crest. The ventral margin of the basioccipital bears a deep median indentation that represents the dorsal half of the median Eustachian foramen (Fig 6B). The left and right margins of the basioccipital are thick and each bears in its ventral third a large, oval foramen that is interpreted here as the lateral Eustachian foramen (Fig 6B). In rostral aspect, the surface of the basioccipital is medially slightly depressed and bears a pair of rounded depressions ventrally (Fig 6E).

The basisphenoid is preserved isolated and segmented with its nearly complete body (Fig 6B), but with a very irregular and incomplete lateral surface. Its occipitoventral part, which lies ventrally adjacent to the basioccipital and is exposed on the occipital surface of the skull, forms left and right a thickened flange with a median incision that together form the ventral half of the median Eustachian foramen (Fig 6B). The ventral part of the basisphenoid is attached to the pterygoid and excluded from the ventral surface of the skull. Thus, the Eustachian tube is entirely enclosed between basioccipital and basisphenoid (Fig 6B). The basisphenoid rostrum is hatchet-shaped, i.e. dorsoventrally expanded and with a slightly convex rostral margin (Fig 6F). In rostral view, the hypophyseal fossa is exposed caudodorsally to the basisphenoid rostrum (Fig 6E). The caudodorsal wall of this fossa, the dorsum sellae (which floors the brain cavity rostrally) is preserved as well, and perforated by two medial, circular anterior carotic foramina (Fig 6E). Rostrolaterally to the anterior carotic foramina, the left and right foramen for the cranial nerve VI are visible (Fig 6E).

#### Laterosphenoid and prootic

Laterosphenoid and prootic are preserved in DFMMh/FV 605 and retrieved from the 3D data (Fig 6F and 6G). The original bones of the braincase are still embedded in matrix and not visible from externally. Both laterosphenoid and prootic are segmented in one piece and separate badly. On the laterosphenoid, the capitate process is visible as a small, laterally oriented knob-like process that contacts the dorsally adjacent postorbital (Fig 6F). Ventrally, the laterosphenoid margin forms a caudoventral indentation, which represents the passageway for the cranial nerve V (trigeminal nerve). The medial face of the laterosphenoid is deeply depressed and forms the cerebral fossa (Fig 6G). From the prootic, only fragments with bad resolution could be segmented (Fig 6F). In medial aspect, the prootic bears a distinct bulging “Y” shaped crest structure (Fig 6G).

### Mandible

#### Form and proportions

**Fig 8:**
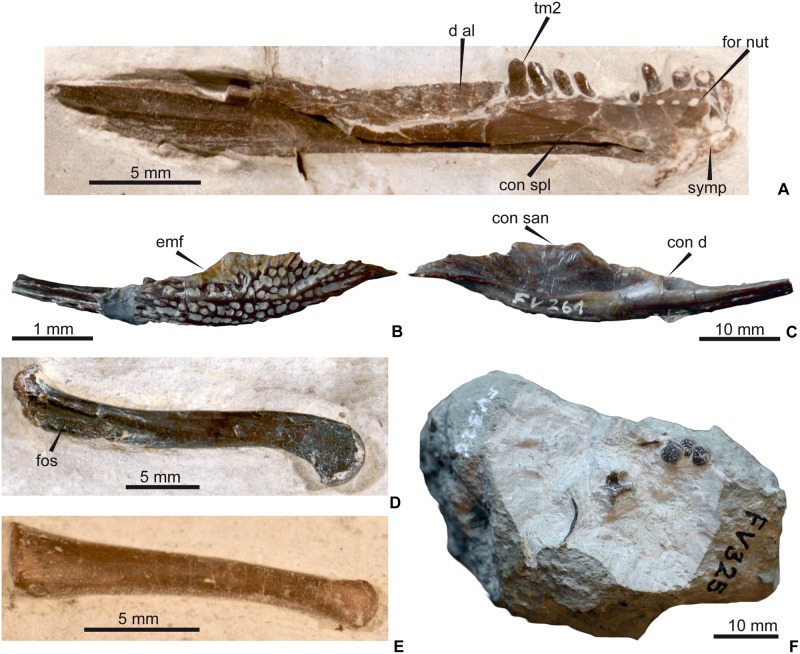
Isolated cranial and postcranial bones atrributed to *Knoetschkesuchus langenbergensis* gen. nov. sp. nov. (A) DFMMh/FV 790.12, left dentary, in medial aspect. (B) DFMMh/FV 261, left angular in lateral aspect. (C) DFMMh/FV 261, left angular in medial aspect. (D) DFMMh/FV 279, right femur, in dorsolateral aspect. (E) DFMMh/FV 790.11, metatarsal in lateral aspect. (F) DFMMh/FV 325, slab with thoracic rib, partial dorsal vertebra and four osteoderms.

In DFMMh/FV 200, the left mandibular ramus is preserved in connection with the skull and is, except for the retroarticular process, exposed from laterally (Fig 4B). In the caudal half of the mandible, the bones collapsed during diagenesis and slid into one another dorsoventrally. In DFMMh/FV 605, both mandibular rami are preserved with exception of the articular part, but are embedded in matrix and therefore exposed only from lateroventrally (Fig 5B). Segmentation of the mandibles made them visible from all sides (Fig 6H, 6I and 6J). DFMMh/FV 790.12 (Fig 8A) is an isolated dentary withouth its rostralmost part, exposed in medial aspect in the sediment. There is also a completely preserved isolated left angular, DFMMh/FV 261 (Fig 8C).

In lateral aspect, the rostralmost and caudalmost portion of the mandibular ramus is slightly bent dorsally (Fig 6H). The lateral margin of the mandibular ramus is slightly convex rostrally to the sixth dentary tooth and becomes caudally to this tooth weakly concave and then straight. From rostrally to caudally the mandibular ramus gradually increases in height to reach caudally twice of its rostral height. The complete mandibular symphysis is preserved only in DFMMh/FV 605, where it extends to the level of the eighth dentary tooth and reveals a participation of the splenial into the symphysis as well as a small caudal peg at the mandibular sympyhsis visible (Fig 6I and 6J). The external mandibular fenestra is collapsed in DFMMh/FV 200. However, a semi-circular indentation at the rostrodorsal margin of the angular and a concave incision of the rostroventral margin of the surangular indicate that an external mandibular fenestra was also present in this specimen (Fig 4B). A well preserved external mandibular fenestra is visible in the segmented μCT data of DFMMh/FV 605 (Fig 6H).

In DFMMh/FV 200, the lateral surface of the dentary is covered with dense pitting rostrally (Fig 4B). Caudally to the 12^th^ dentary tooth, the sculpturing changes into intermittently spaced longitudinally grooves. The sculpturing of the lateral face of the surangular is weak rostrally, comprising only a few, intermittently spaced pits, and increases caudally to longitudinally oval pits. No insertion area for m. pterygoideus posterior is visible from laterally. In DFMMh/FV 605, the rostral third of the mandibular ramus is sculptured with closely spaced circular and longitudinal oval-shaped pits (Fig 6B). The sculpturing becomes less distinct caudally.

#### Dentary

The dentary is very flat in its rostralmost part and increases gradually in height in caudal direction (Figs 4B, 5B and 6H). The dentary extends caudally until mid-length of the orbita. Its medial margin is straight between the rostral tip and the 8^th^ dentary tooth, marking the region of the mandibular symphysis. The rostrolateral margin is convex. In lateral aspect, the dorsal margin of the dentary is convex in its rostral part and becomes straight after the 5^th^ dentary tooth. The caudalmost part of the dentary forms a more narrow median process that wedges out between the dorsal surangular and the ventral angular. This process forms the rostroventral margin of the external mandibular fenestra. The dorsal part of the lateral dentary margin bears a crest in its caudal fourth, which parallels the raising dorsal dentary margin. In DFMMh/FV 200, the rostral part of the dorsal aspect of the left dentary is exposed, showing a part of the tooth row accompanied by a row of oval-shaped nutrient foramina (Fig 4B). Each nutrient foramen is situated between two adjacent alveoli. In DFMMh/FV 790.12 (Fig 8A), the rostral part of the dentary forms medial to the tooth row a shelf that covers the symphyseal region and tapers in caudal direction by its laterally directed margin to end at the 9^th^ dentary tooth. A row of nutrient foramina is placed on the shelf and medially adjacent to each dentary alveolus. In medial aspect, the opening of the Meckelian canal forms a groove, which is situated in the ventral third of the dentary (Figs 6J and 8A).

#### Splenial

The splenial is well exposed only in DFMMh/FV 605, where it could be segmented as a part of the mandible (Fig 6H and 6J). In this specimen, the splenial forms the medial wall of the mandibular rami and the medial wall of the dentary tooth row. The rostralmost part of the splenial bends medially and forms the caudalmost part of the mandibular symphysis. The length of the splenial contribution to the sympyhsis is approximately the length of two dentary alveoli. In ventral view, the splenial contribution to the mandibular symphysis is exposed only in its caudalmost extent and v-shaped in ventral view, with a small caudal peg (Fig 6H). Caudal to the symphysis, the splenial bears a large oval foramen medially. Caudally, the splenial ends at the contact with the angular ventrally and the surangular dorsally, but its caudal end is not completely preserved in DFMMh/FV 605.

#### Angular

The caudal third of the mandibular ramus is formed ventrally by the angular. The isolated angular DFMMh/FV 261 (Fig 8B and 8C) is completely preserved and corresponds in morphology to those of the skulls DFMMh/FV 200 and DFMMh/FV 605 (Figs 4B and 6H). The angular forms a ventral trough that represents the caudal part of the Meckelian canal, with a high lateral wall, which bears a dorsally directed tip slightly caudally to its mid-length. The rostral third of the angular underlaps the dentary with a thin process. Caudally to this process, the lateral angular margin rises slowly dorsally until the caudocentral tip and forms the ventral margin of the external mandibular fenestra. Caudally to that tip, the lateral wall slightly decreases in height and the angular margin is irregularly jagged to suturally contact the surangular. Rostrally to the tip, the dorsal margin is indented and smooth, representing most probably the ventral margin of the external mandibular fenestra. The ventral margin of the angular is convex, which contributes to the rostral and caudal height increase of the angular. The caudal end of the angular forms a tongue-shaped, caudoventrally directed process that constitutes the ventral part of the retroarticular process (Figs 4B and 8B). In medial aspect, the lateral wall of the angular bears parallel to its dorsal margin a fine, dorsoventrally oriented striation. The medial margin of the angular (i.e., medial to the angular trough) is low and retains its height along the bone.

#### Surangular

The surangular forms the caudodorsal part of the mandible laterally to the articular. Because the latter is not preserved, the contact between surangular and articular cannot be determined. The left surangular in DFMMh/FV 200 shows that the lateral surface of the caudal mandible was formed by angular and surangular exclusively (Figs 4B and 7B). The articular with the dentary glenoid must have been attached from medially to the caudodorsal corner of the surangular and does not contribute to the lateral or laterodorsal mandibular ramus. The dorsal margin of the surangular is straight and rostrodorsally oriented. The rostral half of the surangular forms a thin rostrally directed process, the ventral margin of which is smooth and indented and represents the dorsal margin of the external mandibular fenestra (Fig 4B). The rostral process of the surangular underlaps the caudal end of the dentary with its rostral-most portion, forming a through-like dorsal margin (Fig 5H). The caudal half of the surangular is higher and rounded rectangular. The caudal part of the surangular end forms the short retroarticular process, an obliquely caudoventrally directed margin with a very short caudoventral elongation (Fig 4B).

### Dentition

#### Tooth morphology

Two different tooth morphotypes are visible. The first morphotype comprises the premaxillary, first to fifth maxillary and the first to fifth dentary teeth. This morphotype is characterized by conical and slightly lingually curved teeth with a weak basal constriction (Fig 4A and 4B). The base of the tooth crown is circular in cross-section and the apex is pointed. The enamel on the labial surface of the crown bears straight and closely spaced faint basioapical striations. Similar, but stronger striae are developed on the lingual surface. The enlarged pseudocaniniform teeth of the maxilla and the dentary bear a more pointed apex, a stronger basal constriction, and smooth enamel on the labial surface of the tooth crown. Delicate mesial and distal carinae are present.

The second morphotype comprises all maxillary and dentary teeth caudal to the fifth tooth. These teeth have lanceolate tooth crowns, labiolingually compressed and oriented parallel to the longitudinal axis of the skull (Figs 4B and 8A). The tooth basis is constricted. From rostral to caudal, these teeth decrease to half of their initial height, and the apex changes from a tip to a more rounded shape. Weak mesial and distal carinae are present. The enamel on the labial side of the tooth crown is covered with closely spaced basioapical, straight striae, which are restricted to the apical half of the tooth crown. Unfortunately, most teeth of this morphotype are preserved from externally so that the lingual side is obscured mostly by matrix (but see Fig 8A). Lingually exposed are the sixth to eighth dentary teeth, which have a pattern of striation that consists of closely spaced striation lines fanning out from the base to the apical half of the tooth. μCT resolution in DFMMh/FV 605 is not sufficient to depict a pattern of striation on the teeth (Figs 5C, 5D and 6H).

#### Pattern of dentition

Five premaxillary teeth are visible in DFMMh/FV 605 (Fig 5A and 5D). The fourth premaxillary tooth is at least one third larger than the preceding ones, but does not form a distinct pseudocaninus.

The maxilla of DFMMh/FV 200 exposes at least 12 maxillary alveoli (Fig 4B), which are remarked by a festooning of the lateral maxillary margin, but the total amount of maxillary teeth is probably more and corresponds to DFMMh/FV 605. Eight maxillary teeth are preserved *in situ*. The enlarged fifth maxillary tooth forms a pseudocaninus. The sixth maxillary tooth is only half as high as the fifth, but caudal to the sixth, the height of the teeth decreases only moderately.

The maxilla of DFMMh/FV 605 contains ten teeth *in situ* (Fig 5C) with additional maxillary teeth being preserved near the maxilla in the matrix. The 3D data reveal that in this specimen, there are 12 distinct maxillary alveoli with teeth (Fig 5B). Caudal to the 12^th^ maxillary tooth, there are no distinct alveoli, but the teeth sit in a trough and are not separated from each other by interalveolar plates. Not all maxillary teeth are preserved in that trough, and the total amount of maxillary teeth can only be estimated to be 17 or 18. The fourth and fifth maxillary teeth are enlarged (Fig 5C), the other teeth are roughly of similar size and approximately half as high as the enlarged ones.

In the dentary of DFMMh/FV 200, at least 14 tooth positions can be reconstructed, with 10 teeth preserved *in situ* (Fig 4B), but the total amount of teeth was probably similar to DFMMh/FV 605. The two most rostral tooth positions are preserved with a broken base of the tooth crowns. The fourth, preserved, dentary tooth is enlarged, but the caudal dentary teeth are small and reach only three fifth of the height of the pseudocanini. Only the presumably ninth and tenth dentary teeth are considerably larger than rostrally and reach two-thirds of the height of the maxillary pseudocanini. A regular decrease in height occurs in the caudally following dentary teeth.

The dentary of DFMMh/FV 605 exposes the two most rostral dentary teeth, which are half as large as the maxillary pseudocaninus. All dentary teeth are visible in the 3D data (Fig 6I and 6J). The first to tenth dentary teeth sit in separate alveoli, of which the third and fourth dentary alveoli are contiguous to each other and enlarged. The fifth to 10^th^ dentary alveoli are significantly smaller than the fourth, but similar to each other in size. From the 11^th^ dentary tooth onwards, teeth sit in a united trough without interalveolar bone, like in the maxilla. The total number of dentary teeth is 21.

The isolated dentary DFMMh/FV 790.12 exposes 16 alveoli, but the most rostral part of the dentary and tooth row is broken off (Fig 8A). The preserved first to fourth and sixth to nineth dentary teeth are still in their alveoli. The two rostralmost preserved teeth lack their tooth crowns, but appear relatively large. The preserved third, sixth and seventh dentary teeth have the same size and are smaller than the caudally following teeth. The fifth to 10^th^ dentary alveoli ar smaller than the fourth alveolus and similar in size. The caudal part of the dentary shows a trough with slight indentations for the single teeth but no interalveolar bone (Fig 8A).

### Axial skeleton

#### Dorsal vertebrae

Two dorsal vertebrae are preserved in ventral aspect, with the neural arches embedded in the matrix (Fig 3A). The transverse processes are exposed from their ventral side, but seem to be incomplete laterally. The vertebral centra are 2.6 times as long as they are high. The cranial and the caudal articular surfaces of the vertebral centrum are circular in outline. The articular surface is platycoelous, and no depressions, concavities, or condyles are developed (Fig 3A). Both articular surfaces are surrounded by a weakly rugose rim. In ventral aspect, the vertebral centrum is slightly medially constricted. The vertebral centrum is half as high as it is long. The lateral surface of the vertebral centrum is slightly concave. The vertebral centrum and the neural arc are completely fused. The transverse processes are straight laterally directed and slightly taper in lateral direction. They are three-fourth as wide as the vertebral centrum is long.

The neural arch of another dorsal vertebra is preserved in rostral aspect, but nearly completely embedded in sediment. This neural arch exposes a large neural canal with a rounded square outline. The neural spine is visible only from rostrally and seems to be of similar height as the peduncle of the neural arch. The articular surfaces of the prezygapophyses are rather steep and have an angle of approximately 30° to the sagittal plane. Transverse processes are not preserved.

#### Caudal vertebrae

One basal caudal vertebra is preserved with its ventral surface exposed, whereas from another caudal vertebra only the neural arch is exposed in dorsal aspect (Fig 3B). The two caudal vertebrae possess slightly caudally directed transverse processes that are as wide as the vertebral body is long. The transverse processes are completely fused to the vertebral centrum and therefore cannot be mistaken for caudal ribs. In the ventrally exposed vertebra, a haemapophyseal articular surface is developed on the caudal margin of the ventral vertebral centrum. Together with the fused transverse process this suggests that the vertebra is the 4^th^ caudal vertebra, bearing the first caudal transverse process. The caudal neural arch derives, judging by the relative length of its transverse process, from a position close but caudal to the 4^th^ caudal vertebra. The ventral surface of the vertebral centrum is tapering to a rounded, longitudinal crest, which possesses a weak paired and rounded haemapophyseal articular surface caudally. The vertebral centrum is approximately two times longer than it is high. The cranial and caudal articular surfaces of the vertebral centrum are circular in outline and platycoelous. The lateral surface is weakly concave. The transverse process is positioned dorsolaterally on the vertebral centrum and is one third wider than the vertebral centrum is long. The transverse process is slightly caudolaterally directed and tapers laterally. Its cranial margin is very weakly convex, its caudal margin is weakly concave. The neural arch is smooth in dorsal aspect, and the neural spine is positioned in the caudal two-thirds of the neural arch. In lateral aspect, the neural spine is tapering in apical direction and slightly caudally inclined with an angle of 15° from the vertical. Its apex is not preserved.

#### Ribs

Several fragments of ribs are preserved on the slab, and according to their size and morphology they likely come from the prothoracic to the caudal thoracic vertebral column (Fig 3A). The rib bodies are rostrocaudally compressed and only weakly curved. Some of the dorsal ribs bear a cranially directed, triangular process in the proximal third of the rib body (Fig 3C). Capitulum and tuberculum of the preserved thoracic ribs are separated in most cases, but two or three of the caudalmost dorsal ribs bear a synapophysis. The capitulum is slighty shorter and has a smaller articular surface than the tuberculum. The capitulotubercular incision is v-shaped and the external surface of the rib body is weakly depressed between the capitulum and the tuberculum. There are two possible gastralia on the slab, which have a very thin and rather straight rib body with an irregular bone texture (Fig 3A).

### Appendicular skeleton

#### Scapula

The left scapula is exposed from its lateral surface in DFMMh/FV 200. The scapula bears a flat and broadened scapular blade with a neck constricted dorsally to the scapular body (Fig 3A and 3E). The cranial margin of the scapular blade is concave, the caudal margins are straight. The dorsal scapular margin is weakly convex. The glenoid for the humerus is not preserved.

#### Humerus, ulna and radius

On DFMMh/FV 200, the left humerus is partly preserved in craniolateral aspect and with its proximal extremity buried within the matrix (Fig 3A and 3E). In lateral aspect, only the nearly triangular and comparatively short deltopectoral crest is visible. The humerus possesses a straight shaft, of which the proximal and distal extremities are twisted against each other. On the slab, the distal extremity of the humerus is positioned in a gap between radius and ulna. The distal extremity bears two condyles that are separated from each other by a shallow intercondylar sulcus. The medial condyle is roughly one third wider than the lateral condyle, whereas the latter is distally slightly longer. The lateral condyle continues onto the cranial face of the distal extremity of the humerus with a rounded crest. The proximal half of radius and ulna is obscured by matrix. The shaft of the ulna is twice as broad as the shaft of the radius, and its distal extremity is broad (Fig 3A).

#### Metacarpals and manual phalanges

Four left metacarpals and four partially preserved manual phalanges are exposed from their dorsal side in DFMMh/FV 200 medial to the humerus (Fig 3A and 3E). The shaft of the metacarpal I is constricted. The proximal articular surface is broadened and spatulate, and bears a ridge on the middle of its surface. The distal articular surface is destroyed. Metacarpal II is one third shorter than the first and has a stout and broad shaft. Its proximal articular surface is broadened and spatulate, as in metacarpal I. Metacarpal III is comparable with metacarpal I, but metacarpal IV is more slender than metacarpal IV and has a distal articular surface with a single condyle. The manual phalanges vary slightly in size and width, and possess a slightly spatulate proximal articular surface, a weakly constricted shaft and a distal articular surface with two weakly divided hemicondyles (Fig 3E).

#### Ilium and ischium

The left ilium is preserved with a very small fragment of the acetabular region in DFMMh/FV 200 (Fig 3A). The acetabular concavity in this specimen is semicircular in outline and seems to be overhung by the iliac body. No further anatomical details can be retrieved.

The left and right ischium are preserved on DFMMh/FV 200 and exposed in medial view (Fig 3A and 3D). The ischium has a dorsally constricted shaft that bifurcates into the iliac and the pubic process. Both processes are separated from each other by a semi-circular incision that represents the ventral margin of the acetabulum (Fig 3D). The iliac process is flattened and directed dorsocaudally from the long axis of the ischiadic shaft. The pubic process is only half as broad as the iliac process, and directed from the ischiadic shaft at an obtuse angle of about 120° in craniodorsal direction. Its exposed pubic articular surface is circular in outline. The acetabular part of the ischium is 1.5 times as long as the shaft. The ischiadic shaft expands from its dorsal constriction in ventral direction to form a flat and broad ischiadic blade, which is half obscured in the sediment. The cranial and caudal margins of the ischiadic shaft are concave. The ischiadic blade possesses a straight ventral margin that is slightly caudally elongated. The presence of the separate pubic articular surface of at the cranial/ischiadic pubic process suggests that the pubis was completely excluded from the acetabulum.

#### Femur

A left femur is partially visible in lateral aspect in DFMMh/FV 200 (Fig 3A). The proximal extremity and most of the femoral shaft and distal extremity are obscured by matrix. The femoral shaft is straight with the distal extremity slightly bent cranially. The lateral articular condyle is exposed from laterally and shows an ectepicondylar rugosity.

An isolated right femur (DFMMh/FV 279) is visible in lateral aspect (Fig 8D). The bone is sigmoidally curved (Fig 8D). The proximal and the distal extremities are twisted at about 145° against each other. The femoral head is cranially inclined with an angle of 30° to the long axis. The femoral shaft is circular in cross-section and becomes more oval-shaped distally. The articular surface of the femoral head has a semi-circular proximal margin, which overhangs the medial surface of the femoral head. The distal extremity of the femur is divided into a lateral and medial condyle, separated from each other by an intercondylar fossa. The caudal part of the intercondylar fossa is covered with sediment. The lateral condyle is one third larger than the medial one (Fig 8D). A deep and rugose, proximodistally oriented fossa is developed proximal to the distal extremity on the cranial surface of the bone. The fossa extends for approximately one third of the femoral length and reaches its maximum width dorsal to the distal extremity, tapering proximally. The fourth trochanter is not visible.

#### Tibia and fibula

The left tibia obscures the underlying fibula except for a marginal part of its proximal half (Fig 3A and 3E). The tibia is broken medially and both parts are slightly displaced from each other. The tibia reaches two-thirds of the length of the humerus. The tibia is a slender bone that has a slightly expanded proximal and distal extremity (Fig 3E). The tibial shaft is slightly arched and in cross-section twice as broad as the fibula. The lateral face of the proximal extremity is irregularly rugose.

#### Metatarsals and phalanges

Four left metatarsals are preserved in DFMMh/FV 200 (Fig 3A). Metatarsal I and IV are preserved isolated and metatarsal II and III are overlying each other with the second one obscuring large parts of the third one. Metatarsal I is half as long as the other three. Metatarsal II to IV are roughly as long as the tibia. Metatarsal I has a strongly constricted, slender shaft with broadened distal and proximal extremities. The proximal extremity is flattened and dorsally rugose. Metatarsal II has a straight and flattened shaft that comprises two thirds of its total length. Laterally at the distal extremity, an elongate oval rugose depression is present. The shaft is transversely oval in cross-section. Metatarsal IV is more slender than the second one, and possesses a straight shaft with oval cross-section. Its proximal extremity is very weakly expanded and spatulate, and dorsally slightly rugose. The distal extremity shows a characteristic spool-shape with a square articular surface divided into two hemicondyles separated by a weak median sulcus, and lateral and medial circular depressions.

The isolated metatarsal (DFMMh/FV 790.11) has a straight shaft that comprises three quarters of the total length of the bone (Fig 8E). The proximal extremity of the metatarsal is approximately twice as wide as the shaft and spatulate (Fig 8E). The distal extremity of the metatarsal is slightly wider than the shaft and possesses a lateral and a medial condyle with an external, rounded depression. The palmar surface of the distal extremity bears a shallow circular depression between the condyles.

Only one pedal phalanx is preserved in DFMMh/FV 200 (Fig 3A). The phalanx has a shaft that forms half of the total length of the bone, with a base that is slightly wider than the shaft. The proximal metacarpal articular surface is oval in outline and centrally deeply concave. The distal metacarpal extremity is slightly wider than the shaft.

#### Osteoderms

The four preserved paravertebral osteoderms of DFMMh/FV 200 are rectangular in outline and 1.5 times as long as they are wide (Fig 3A, 3D and 3E). The medial margin of each osteoderm is straight, the other margins are very weakly convex. The external surface of the paravertebral osteoderms is strongly sculptured with circular pits and bears cranially a short smooth and “swollen” higher area of bone that might represent a zone of imbrication with the cranially adjacent osteoderm (Fig 3D). The internal surface is unsculptured, but bears a few faint striations, which could be the marks of the underlying and attaching Sharpey’s fibres of the dorsal musculature (Fig 3E). The osteoderms are slightly vaulted dorsally and have a barely visible shallow keel developed. A fifth osteoderm with a more rounded square shape lacks the cranial imbrication zone, and probably derives from the neck or tail base region of the specimen. On the isolated slab DFMMh/FV 325, the preserved osteoderms are rounded squarish in outline and lack an imbrication zone (Fig 8F), as described above for the fifth osteoderm.

## Phylogeny

84 crocodylomorph taxa were coded for 321 morphological characters. The matrix was taken from Turner [21], but several changes in the coding were made to adapt it for the analysis of this taxon (see supplementary data for detailed documentation of changes). The total number of taxa used here is smaller than in the original matrix of Turner [21], as the focus of this study lay on the Neosuchian relationships of *Knoetschkesuchus langenbergensis*. *Theriosuchus grandinaris* [53] and the new taxon were added to the matrix. *Theriosuchus ibericus* has been left out due to the pending re-description of this taxon (Tennant et al. pers comm). *Theriosuchus guimarotae* (*Knoetschkesuchus guimarotae* new combination in this work) was re-coded due to poor previous coding in the matrix of Turner [21] (S1 Text, S1 Matrix). The poor coding of this taxon by Turner [21] is simply the result of insufficient image quality in the original publication [21] (i.e., the pdf was set to a very low resolution and printing quality that obscured the fine anatomical details), lack of the description of some data that could be added now (DS pers obs) and finally some mistakes in the reconstructional drawing of the skull in Schwarz & Salisbury [21] (see Ontogeny for explanation).

TNT v. 1.5 [54, 55] was used for the phylogenetic analysis, following in parts the procedure described by Turner [21]. The tree was rooted on *Gracilisuchus stipanicicorum* as an outgroup taxon. All characters in the matrix were used unmodified, so no status like additive or inactive was chosen. During the analysis, the taxon *Elosuchus cherifiensis* turned out to severely influence the stability of the trees and therefore has been excluded within TNT directly. With the remaining 83 taxa, a heuristic search with equally weighted parsimony was carried out, calculating Wagner trees with 10000 replicates (with random addition sequences = RAS), and a following TBR branch swapping with holding 10 trees per replicate. The best trees obtained at the end of the replicates were subjected to TBR branch swapping again. Zero-length branches were collapsed if they lacked support under any of the most parsimonious reconstructions (“rule 1” of consense options in TNT [54, 55]).

The heuristic search included a total number of 18931860 rearrangements and produced 18 most parsimonious trees (MPT) with a tree length of 1418 steps each, a consistency index (CI) of 0.279, and a retention index (RI) of 0.634. The character support of the nodes in the most parsimonious reconstructions was calculated with the technique of Bremer support in TNT, and is visible as a consensus tree (S1 Fig).

**Fig 9:**
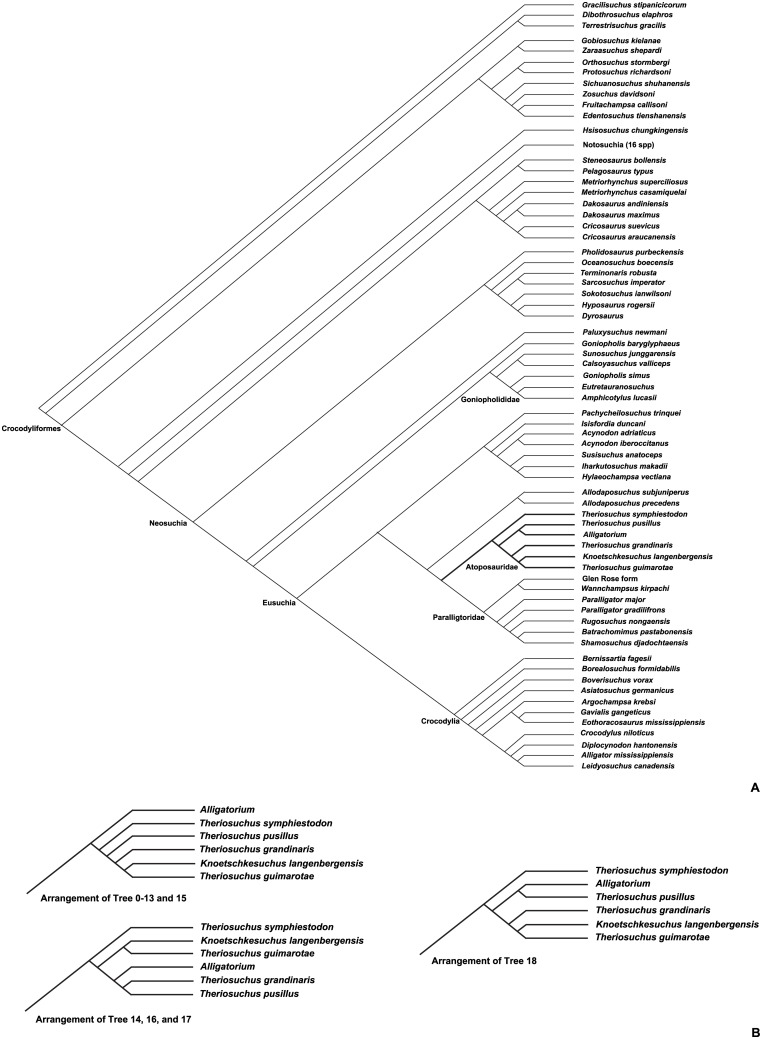
Results of the phylogenetic analysis with focus on *Knoetschkesuchus* gen. nov. (A) Single most parsimonious tree resulting from maximum parsimony analysis (MPA) of 82 ingroup taxa and 321 phenotypic characters (tree length is 1669 steps, CI = 0,874, RI = 0,976). Atoposauridae is marked bold in here. (B) The three alternative arrangements within Atoposauridae out of the 18 most parsimonious trees resulting from the heuristic search of the matrix.

A Maximum Parsimony Analysis (MPA) was done with the same matrix, resulting via 674652933 rearrangements in only one, very stable tree with a tree length of 1669 steps, a CI of 0,874, and aRI of 0,976 (Fig 9). Jackknife support analysis and bootstrap analysis were run in TNT on the character matrix. The jackknife support analysis was performed as in Turner [21], using 1000 replicates of RAS followed by TBR branch swapping (saving 10 trees per replicate). The bootstrap analysis was done with a simple addition replicate and 1000 bootstrap replicates. Tree topologies obtained both from jackknifing and bootstrapping are summarized using GC frequencies (S2 Fig).

In all of the obtained trees, *Knoetschkesuchus langenbergensis* clusters with *Theriosuchus* and *Alligatorium* in a clade that depicts Atoposauridae. The unit of Atoposauridae is supported by Jackknifing and Bootstrap analysis, but has very low Bremer support. Within the 18 trees of the heuristic search, three different topologies within Atoposauridae are possible (Fig 9). *Knoetschkesuchus langenbergensis* and *Knoetschkesuchus guimarotae* (new combination) form a very stable sister group relationship with high support values (S1 and S2 Figs). Most of the topologies do also indicate a close relationship between a clade consisting of *Knoetschkesuchus langenbergensis* and *Knoetschkesuchus guimarotae* new combination, and *Theriosuchus grandinaris*, which has also high support values in the Boostrap and Jackknife analysis (S1 Fig). *Theriosuchus pusillus*, *T*. *symphiestodon* and *Alligatorium* are swapping their positions and have lower support values.

Whereas most previous phylogenetic analyses have placed Atoposauridae in a position near the basis of all Neosuchia (e.g., [19, 24, 28, 56–58], more recent works have placed them as a sister group to Paralligatoridae, within Eusuchia and together with Paralligatoridae as the sister group to Crocodylia [21, 23]. In consistence with the analysis provided by Turner [21], the clade Atoposauridae in this work (Fig 9) is nested deeply within Eusuchia, and closely related to the clade Paralligatoridae. Although the obtained most parsimonious tree is in many aspects similar to the strict consensus tree represented by Turner [21: fig. 14], there are some differences in the arrangements of single clades that might be the result of a smaller amount of taxa used and corrections of the coding. The main groups Crocodyliformes, Neosuchia, and Eusuchia are similarly depicted. The main differences refer to the placement of Susisuchidae, which are in contrast to the placement within Neosuchia in Turner [21] included within Eusuchia and part of a larger clade comprising also Hylaeochampsidae in the work here (Fig 9). In contrast to Turner [21], also both coded species of *Allodaposuchus* cluster together as closely related to Atoposauridae. *Bernissartia* is positioned directly at the base of Crocodylia, but the support values are not good for this arrangement.

## Discussion

### Ontogeny

The material described from *Knoetschkesuchus langenbergensis* represent specimens of at least three different body sizes. The cranial material of specimens DFMMh/FV 200 and DFMMh/FV 605 represents different ontogenetic stages (see below) and sizes. The isolated angular DFMMh/FV 269 is larger than the articulated angular of specimen DFMMh/FV 200, whereas the isolated postcranial material is comparable in size to DFMMh/FV 200. The minimum number of indiviuals thus would be three, based on size differences. Ontogenetic traits are based on both proportions and morphology.

Proportional changes are described with biometric skull ratios (S1 Table). The proportional lengthening and widening of the rostral region of the skull is described 1.) by the ratio between rostral skull length and total skull length, which changes from 0.37 in the smaller skull (DFMMh/FV 605) to 0.47 in the larger skull (DFMMh/FV 200); and 2.) by the ratio between length and width of the rostrum of the skull, which is 1.37 in DFMMh/FV 605 and 1.50 in DFMMh/FV 200. The rostral region of the skull in *Knoetschkesuchus langenbergensis* lengthens proportionally in relation to the total skull length, as well as in relation to its width (Fig 7A and 7C). These changes correspond to extant crocodylians, which are initially extremely brevirostrine with a high length/width ratio of the rostrum, then grow in rostral length [52, 59, 60]. In older ontogenetic stages, the width of the rostral region in extant crocodylians increases again [52, 59, 60], which might explain why the difference between DFMMh/FV 605 and DFMMh/FV 200 is not too big.

The ratio between the width and the length of the cranial table is 0.64 in the smaller skull (DFMMh/FV 605) and 0.53 in the larger skull (DFMMh/FV 200), indicating that the cranial table gets wider during growth as in extant crocodylians [52, 59, 60]. The ratio between the rostral width of the cranial table and the caudal width of the cranial table is 0.95 in the smaller skull (DFMMh/FV 605) and 0.66 in the larger skull, which is due to the increasing converging of the lateral margins of the cranial table in rostral direction as in extant crocodylians [60].

Orbital size is proportially larger in early ontogenetic stages of extant crocodylians [59, 60] and described as ratio between orbital length and total skull length. The ratio between orbital length and total skull length changes from 0.28 in the small skull (DFMMh/FV 605) to 0.32 in the large skull (DFMMh/FV 200), which is not significant and demonstrates that the orbit remains rather large in relation to the total skull dimensions. A small size-reduction of the orbit during ontogeny is known from atoposaurids in general [15, 17, 61], and might represent paedomorphism.

**Fig 10:**
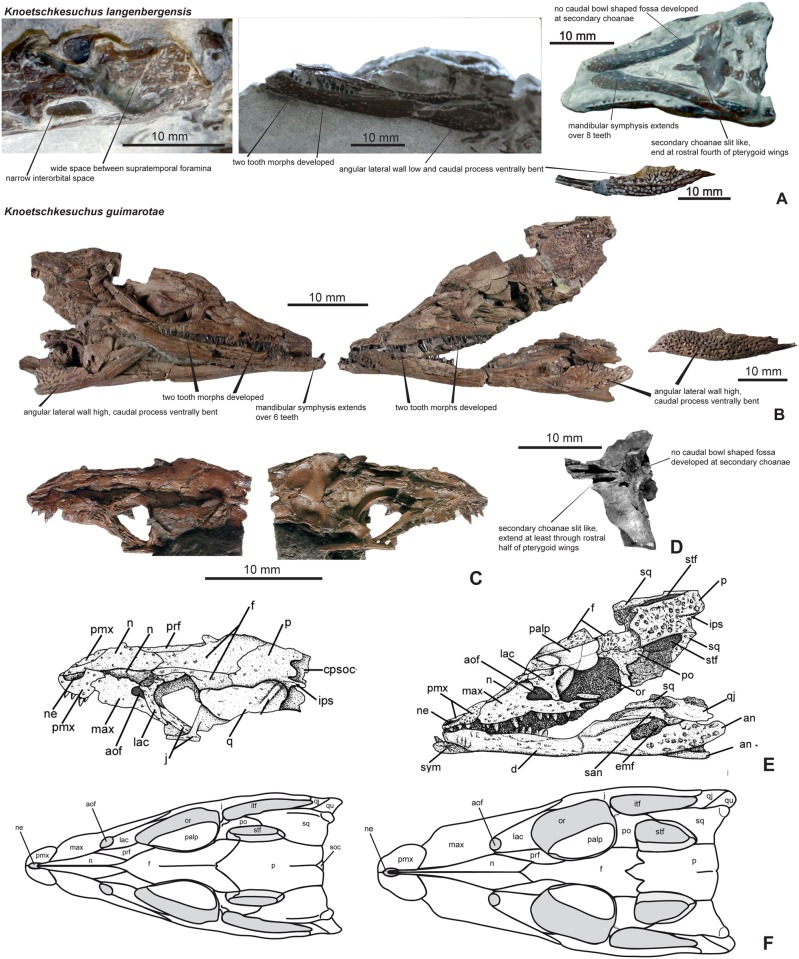
Anatomical comparisons between *Knoetschkesuchus langenbergensis* gen. nov. sp. nov. and *Knoetschkesuchus guimarotae* (new combination). (A) Some diagnostic cranial elements of *Knoetschkesuchus langenbergensis* gen. nov. sp. nov. with important characters denoted, from left to right: skull DFMMh/FV 200 in dorsal and left lateral aspect, skull DFMMh/FV 605 in ventral aspect, and left angular DFMMh/FV 261 in lateral aspect. (B) Holotype skull IFPUB Gui Croc 7308–1 and isolated angular IPFUB Gui Croc 8044 of *Knoetschkesuchus guimarotae* new combination, with important characters denoted. (C) Juvenile skull IFPUB Gui Croc 7309 of *Knoetschkesuchus guimarotae* new combination. (D) Isolated pterygoid IPFUB Gui Croc 7501 of *Knoetschkesuchus guimarotae* new combination, in ventral aspect and with important characters denoted. (E) Interpretative drawings of skulls IFPUB Gui Croc 7309 (left) and IFPUB Gui Croc 7308–1 (right) of *Knoetschkesuchus guimarotae* new combination, from Schwarz & Salisbury [IFPUB Gui Croc 7309, 61: Fig. 2]. (F). Reconstructions of skulls DFMMh/FV 605 (left) and DFMMh/FV 200 (right) of *Knoetschkesuchus langenbergensis* gen. nov. sp. nov. for comparison with similar ontogenetic stages of *Knoetschkesuchus guimarotae* new combination as visible in (E). Note in particular the similarities in shape changes of the lacrimal and postorbital, and the proportional changes in the cranial table. All photographs of *Knoetschkesuchus guimarotae* are courtesy of DS and have been published in Schwarz & Salisbury [IFPUB Gui Croc 7309, 61: Fig. 2]. For anatomical abbreviations, see text.

Shape change of the supratemporal foramen during ontogeny have been documented by a number of authors for extant crocodylians [52, 59, 60, 62], and are also visible in *Knoetschkesuchus*. The shape of the supratemporal foramen is narrow oval and 54% longer than wide in DFMMh/FV 605, but more rounded rectangular and 42% longer than wide in DFMMh/FV 200 (Fig 7A and 7C). A similar, ontogenetic change in the outline of the supratemporal foramen is visible in specimens of *K*. *guimarotae* (new combination) [61] (Fig 10C and 10F). Thus, coding the shape of the supratemporal foramen for a phylogenetic analysis (see Character 254 in S1 Text) might require using multiple stages for one character. The change in the outline of the supratemporal foramen does not influence the proportional contribution of the surrounding bones to the margin of the foramen. In particular the parietal retains its mostly medial contribution to the foramen’s margins and does not expand caudolaterally in the larger specimen DFMMh/FV 200. In contrast, the proportional changes of the supratemporal foramen influence the absolute and relative width of the intrafenestral bar formed by the parietal and rostrally by the frontal [52, 59]. Because of the expansion of the supratemporal foramen, the frontal becomes restricted to the rostromedial tip of the supratemporal foramen in DFMMh/FV 200 (Fig 7A and 7C). The medial part of the postorbital is broader in DFMMh/FV 200 than in DFMMh/FV 605 and sculptured, and it restricts the frontal contribution to the rostromedial corner of the supratemporal foramen (Fig 7A and 7C). The squamosal surface on the cranial table is reduced by the expansion of the supratemporal foramen in DFMMh/FV 200, as is the parietal surface (Fig 7A and 7C). Exactly similar modifications are visible in the cranial table of *Knoetschkesuchus guimarotae* (Fig 10F), whereas the former skull reconstruction of *Knoetschkesuchus guimarotae* [IFPUB Gui Croc 7309, 61: Fig. 2] does not correctly depict the relationships between parietal, frontal, and postorbital on the skull roof (DS pers obs). All in all, the observations made here contradict the statement of Schwarz & Salisbury [61] that the shape and the size of the supratemporal foramina would not change in *K*. *guimarotae* (new combination) during ontogeny.

Skull bone sculpturing is deeper in the larger skull of *Knoetschkesuchus langenbergensis* (DFMMh/FV 200) and the isolated angular DFMMh/FV 369, whereas the smaller specimen DFMMh/FV 605 possesses only faint sculpturing, which indicates an ontogenetically younger age [52, 59]. The frontals are fused in the smaller specimen DFMMh/FV 605. In comparison with DFMMh/FV 200, the frontals of DFMMh/FV 605 are wider between the orbits and on the cranial table. In both skulls, the parietals are fused, but the former median suture is still visible as a median fusion line. In comparison, in extant *Alligator*, frontals and parietals are unfused after hatching and fuse only until young adult stage [62, 63].

The probably most striking ontogenetic change of a skull element is visible in the lacrimal, which is caudolaterally reduced to a narrow process in DFMMh/FV 605, but wider in DFMMh/FV 200 (Figs 4C, 5C, 5D, 7A, 7B and 7C). The lacrimal of DFMMh/FV 605 is completely excluded from a nasal contact by the prefrontal. In DFMMh/FV 200, the lacrimal contacts the nasal in a very narrow tip (Fig 7A). A similar shape change of the lacrimal is visible in *Knoetschkesuchus guimarotae* (Fig 10E and 10F).

The supraoccipital is dorsally exposed on the cranial table in DFMMh/FV 605, as in the smallest specimen of *Knoetschkesuchus guimarotae* [IFPUB Gui Croc 7309, 61: Fig. 2]. In contrast to *Theriosuchus pusillus*, *K*. *langenbergensis* and K. guimarotae have a very reduced sharp ridge on the lateral surface of the angular [21: char. 219]. As the size of skull ridges increases in ontogenetically older extant crocodylians [59], this is most probably ontogenetic.

**Fig 11:**
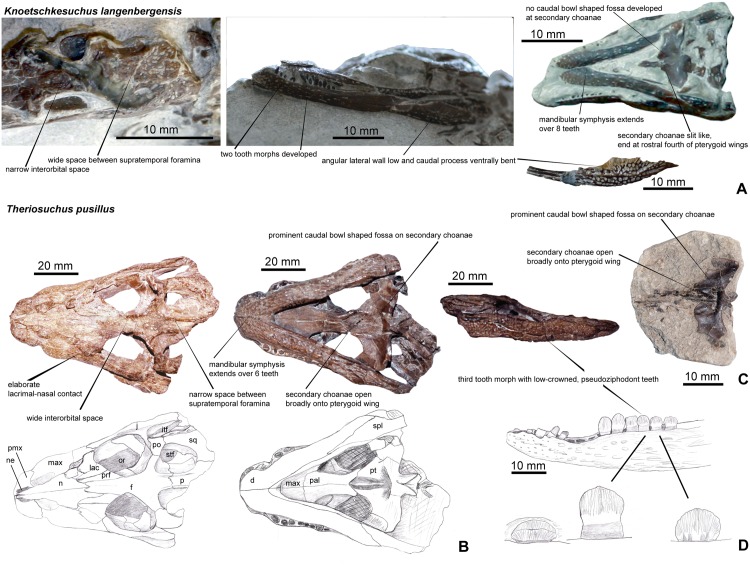
Anatomical comparisons between *Knoetschkesuchus langenbergensis* gen. nov. sp. nov. and *Theriosuchus pusillus* from the Purbeck Limestone Group of Dorset, England. (A) Corresponding to Fig 8A, cranial elements of *Knoetschkesuchus langenbergensis* gen. nov. sp. nov. with important characters denoted, from left to right: skull DFMMh/FV 200 in dorsal and left lateral aspect, skull DFMMh/FV 605 in ventral aspect, and left angular DFMMh/FV 261 in lateral aspect. (B) Paratype skull BMNH 48330 of *Theriosuchus pusillus*, photograph above and interpretative drawing with identified bones below, left in dorsal aspect and right in ventral aspect, with important characters denoted. (C) Isolated pterygoid BMNH 48224 of *Theriosuchus pusillus*. (D) Dental characters of *Theriosuchus pusillus*, on photograph of right lateral aspect of BMNH 48330, and interpretative drawing of left mandibular ramus BMNH 48262, with characteristic teeth enlarged. All photographs and drawings of *Theriosuchus pusillus* are courtesy of DS. For anatomical abbreviations, see text.

According to the observations discussed above, DFMMh/FV 605 represents a specimen of rather young ontogenetic age with partly unfused sutures, slight dermal ornamentation, and significantly juvenile skull proportions. In contrast, the dense sculpturing on the bone surface, more “adult” skull proportions and the fused sutures of the dorsal vertebrae identify DFMMh/FV 200 as a subadult or adult specimen [61, 64] close to maturity. The angular DFMMh/FV 269 belongs to another, still older and larger specimen. Traits not discussed here as ontogenetic variation are significant for taxonomy and phylogeny of *Knoetschkesuchus langenbergensis* and are discussed below by comparisons with *Theriosuchus* (Figs 10 and 11).

### Taxonomic relationships with *Theriosuchus*

#### Previous diagnoses of *Theriosuchus*

In a first short description of DFMMh/FV 200 and DFMMh/FV 605, Karl et al. [8] tentatively assigned the material to *Theriosuchus* based on a brevirostrine skull, divided external nares, orbits larger than supratemporal foramina and a laterally convex and beveled lateral squamosal margin. A tentative assignment to the type species of *Theriosuchus*, T. *pusillus* OWEN 1878, was presumably made based on the similar osteodermal armor and overall similarities in dentition, but the authors stated that both specimens differed from *T*. *pusillus* in the verticalization of the occiput and reduced types of tooth morphology, which they considered to be likely a result of ontogenetic changes [8]. Our analysis of the specimen with inclusion of μCT data shows that the material from Langenberg unambiguously differs from *T*. *pusillus* (see below). Moreover, because there are so many differences to the type species *T*. *pusillus*, the genus *Theriosuchus* is discussed first.

Two specimens of *Theriosuchus* are complete enough to serve as a basis for a genus diagnosis [61]: BMNH 48330, a nearly complete and three-dimensionally preserved skull and BMNH 48216, a partial skeleton, both coming from the Lower Cretaceous (Berriasian) Purbeck Limestone Group of Dorset, southern England. Both specimens were described by Owen [65] as syntypes [26]. Salisbury [26] considered BMNH 48216 as the lectotype and BMNH 48330 as the paratype. A third specimen, the left mandibular ramus BMNH 48328 originally described as *Brachydectes minor* by Owen [65] and renamed as *Oweniasuchus minor* by Woodward [66] for reasons of preoccupation, provides further anatomical information not preserved on BMNH 48216 and BMNH 48330. According to Clark [16], Salisbury [26] and Schwarz and Salisbury [61] specimen BMNH 48328 is considered as a junior synonym of *T*. *pusillus*. Other taxa that have been more recently described and have added some information to the generic diagnosis are *T*. *ibericus* [67], *T*. *grandinaris* [53] and *T*. *symphiestodon* [57, 68]. Bone material referred to *Theriosuchus* by Buscalioni and Sanz [69] and Wu et al. [70] is too fragmentary to give information on the taxonomic status of *Knoetschkesuchus* [61].

According to Owen [65, 71]; Joffe [64]; Buffetaut [72–74]; Clark [16]; Buscalioni and Sanz[18]; Norell and Clark[75]; Brinkmann [67]; Wu et al. [70]; Salisbury [26, 76]; Salisbury and Frey [77], Schwarz and Salisbury [61], and in particular Salisbury & Naish 2011 [27], the genus *Theriosuchus* is defined by a character combination of (1) a brevirostrine skull, with the maxillary rostrum forming between 40% and 45% of the total length of the skull; (2) small antorbital fenestra; (3) slit-like, horizontally oriented and rostrally positioned external nares divided caudally by a median nasal spine; (4) a shallow dorsal sulcus on maxilla positioned caudally to the junction between the maxilla, premaxilla and nasal; (5) a proportionally long jugal in comparison to other atoposaurids; (6) medial base of the postorbital process formed by the ectopterygoid; (7) median crest on the frontal and the parietal partially in later ontogenetic stages; (8) frontal and parietal partially unfused in early ontogenetic stages; (9) supratemporal foramen smaller than the orbita throughout ontogeny; (10) lateral margin of squamosal beveled ventrally; (11) proportionately narrow quadrate possessing a concave mandibular articular surface; (12) secondary choanae bounded by the palatines rostrally and separated by a median septum of the pterygoid; (13) mandibular symphysis restricted in length and not exceeding the rostral six dentary teeth; (14) short praeacetabular process and long postacetabular process of ilium; (15) biserial dorsal shield comprising parasagittal osteoderms. *Knoetschkesuchus langenbergensis* concurs with this list of characters except of 13) length of mandibular symphysis, and 14), which is not preserved. With respect to the definition of characters given above [27 and others], *Knoetschkesuchus* would therefore belong to the genus *Theriosuchus*. In contrast, Young et al. [78] note that a number of characters from this list is present also in other atoposaurids and therefore not useful as unambiguous definition for *Theriosuchus*. According to Young et al. [78], *Knoetschkesuchus* would a definite member of Atoposauridae, but not of *Theriosuchus*.

A central feature discussed for *Theriosuchus* is its dentition. *Theriosuchus* teeth are described to comprise three morphotypes, which are all developed at least in the type species *T*. *pusillus*. These tooth morphs comprise slender and conical teeth, followed by lanceolate and slightly compressed and striated teeth, and low-crowned and strongly compressed leaf-shaped teeth [27, 53, 57, 61, 64, 65, 67, 68, 74, 79, 80]. As the discussion below will show, *Knoetschkesuchus* deviates in the dentition from *Theriosuchus*.

Another character always discussed for *Theriosuchus* is the potential presence of procoelous vertebral centra. DFMMh/FV 200, the larger specimen of *Knoetschkesuchus langenbergensis* possesses platycoelous thoracic, lumbar and caudal vertebrae. According to Salisbury and Frey [76, 77],*T*. *pusillus* possesses gently amphicoelous thoracic and lumbar vertebrae and an opisthocoelous first caudal vertebra, which has often led to the placement of *Theriosuchus* at the base of Eusuchia. The description of procoelous caudal vertebrae for *T*. *ibericus* [67] is doubtful, because no definite assignment of the described vertebrae to the cranial remains of *T*. *ibericus* can be made—the material has been found within the same locality, but isolated. Although procoelous caudal vertebrae are present in the Uña crocodylian assemblage [67], the taxon or taxa to which these elements belong cannot be proven conclusively [61, 76, 77]. All preserved cervical, dorsal, sacral and caudal vertebrae of *K*. *guimarotae* are amphicoelous [61]. Concluding from the uncertain morphology of the intervertebral articular surfaces in different species of *Theriosuchus*, this character is not used here as a diagnostic feature.

#### The diagnosis for *Theriosuchus* given by Young et al. [78]

Recent work demonstrates that the monophyly of the genus *Theriosuchus* is hitherto not fully understood [78]. Because several of these characters are not exclusive for the genus *Theriosuchus* or imprecise, a new and revised diagnosis of *Theriosuchus* was provided by Young et al. [78], using a newly described specimen from the Middle Jurassic of the Isle of Skye in Scotland. This diagnosis is a list of nine autapomorphic characters restricted exclusively to *Theriosuchus* and occurring in particular in the type species *T*. *pusillus* [78]. Each of these characters is discussed in the following with respect to *Knoetschkesuchus*:

1. “Heterodont dentition, with pseudocaniniform, labiolingually compressed, and lanceolate tooth crown morphotypes (labiolingually compressed teeth are absent in *T*. *guimarotae*)” [78]. The specimens DFMMh/FV 200, DFMMh/FV 605, and DFMMh/FV 790.12 possess teeth that are identical to the pseudocaniniform and lanceolate tooth crowns present in *Theriosuchus*. Strongly labiolingually compressed, leaf shaped teeth that typically occur in *T*. *pusillus*, *T*. *grandinaris*, *T*. *symphiestodon*, and *T*. *ibericus* (Fig 11D) are absent both in *Knoetschkesuchus langenbergensis* and *K*. *guimarotae*. The absence of this tooth morphotype might on the one hand be an effect of ontogenetic variation and referable to size differences. However, on the other hand the absence of the third tooth morphotype in all available ontogenetic stages both in K. *guimarotae* and in *K*. *langenbergensis* makes this possibility unlikely. Additionally, among the large number of isolated crocodyliform teeth from the Guimarota mine, there is not a single labiolingually compressed tooth known [61, 81], and this holds also true for the Langenberg quarry [6, 7]. The presence of only two morphotypes of teeth with absence of labiolingually compressed teeth is therefore treated here as a taxonomic character uniting both *K*. *langenbergensis* and *K*. *guimarotae*, and separating the genus from all other species of *Theriosuchus*, in particular *T*. *pusillus*.
2. “‘Low-crowned’ teeth present (absent in *T*. *guimarotae* and *T*. *grandinaris;* Schwarz & Salisbury, 2005)” [78]. *Knoetschkesuchus langenbergensis* does not possess low-crowned teeth, in contrast to the type species *T*. *pusillus* [26, 27], *T*. *symphiestodon* [57, 68], and *T*. *ibericus* [67]. The absence of the character of low-crowned teeth unites *K*. *guimarotae* and *K*. *langenbergensis*, and stands against an affiliation of *Knoetschkesuchus* with the genus *Theriosuchus* (Fig 10D).
3. “Progressive reduction in alveolus size from the fourth to sixth dentary alveoli” [78]. In DFMMh/FV 605 and the isolated dentary DFMMh/FV 790.12 of *Knoetschkesuchus langenbergensis*, the dentary teeth decrease in height starting from the fourth (largest) dentary alveolus (Figs 6J and 8A). Alveolus sizes are large between the first and fourth dentary alveolus, but significantly smaller and of similar size between the 5^th^ to 10^th^ dentary alveoli. *Knoetschkesuchus langenbergensis* and *K*. *guimarotae* differ in this character from the genus *Theriosuchus*.
4. “Presence of false denticles (serrations created by the superficial enamel ornamentation: pseudoziphodonty, *sensu* Prasad & Lapparent de Broin [82],) on the posterior teeth” [78]. None of the described specimens of *Knoetschkesuchus langenbergensis* possesses false denticles and a serration of the carinae. Although not excluded from the diagnosis by Young et al. [78], *K*. *guimarotae* has only unserrated, non-pseudoziphodont teeth with smooth carinae [61] (Fig 10B and 10C).
5. “Some of the dentary alveoli form a confluent chain (not visible in the Skye specimen) from dental alveolus D4-D8” [78]. This character is present in *Knoetschkesuchus langenbergensis*, in which the fifth to tenth dentary alveoli are positioned close to each other and separated only by a thin interalveolar septum (Fig 8A).
6. “Enlarged fifth maxillary tooth, typically with a corresponding notch on the dentary (not enlarged in *T*. *grandinaris*; only moderately enlarged in *T*. *guimarotae* and *T*. *pusillus*; unclear in the Skyle specimen)” [78: p. 4]. The fifth maxillary tooth of *K*. *langenbergensis* is enlarged in comparison to the other teeth, although the corresponding notch at the dentary is not well developed (Figs 4B, 5C and 5D). The exceptions from this character make it possible that this condition is only well developed in *Theriosuchus pusillus* and *T*. *ibericus* and cannot be used for a taxon definition of *Theriosuchus*.
7. “Maxillary and dentary alveolar size is strongly heterogeneous” [78]. Heterogeneity of alveolus size is present in the maxilla and dentary of *Knoetschkesuchus langenbergensis* and *K*. *guimarotae* (Figs 6J and 8A).
8. “Dentary external surface is ornamented with heterogeneously spaced pits, and ventrolaterally rugose” [78]. This is a character visible also in the dentaries of *Knoetschkesuchus langenbergensis* and *K*. *guimarotae* (Figs 4B and 5B).
9. “Supratemporal fenestrae and fossae proportionally large with respect to primary orbit (supratemporal fossae length can exceed two-thirds of orbit length in dorsal view)” [78]. Although the size of the orbit in relation to the size of the supratemporal fenestra is slightly smaller in the large specimen of *Knoetschkesuchus langenbergensis*, this character is present and well developed in *K*. *langenbergensis*.

The diagnosis given by Young et al. [78] for *Theriosuchus* relies mainly on dental characters, and *Knoetschkesuchus langenbergensis* differs from it in four of nine characters given, making an assignment to this taxon impossible. Additionally, if *Knoetschkesuchus* cannot be attributed to *Theriosuchus*, then the characters coinciding with the diagnosis of Young et al. [78] would not be autapomorphic for *Theriosuchus* and need to be removed from the diagnosis of this taxon.

#### Comparisons with species of *Theriosuchus*

*Knoetschkesuchus langenbergensis* differs significantly from the type species *T*. *pusillus* and other species of *Theriosuchus* by the absence of labiolingually compressed, “leaf shaped”, low-crowned, pseudoziphodont teeth, and differences in dentary alveolus size. *Knoetschkesuchus langenbergensis* differs from all known species of *Theriosuchus* by the more rostral position of the secondary choanae, which lie more in the palatines and the rostral process of the pterygoid, but only to a small extent between the pterygoid wings (Figs 5B, 6C, 10D, 11B and 11C). *Knoetschkesuchus langenbergensis* can be furthermore distinguished from all species of *Theriosuchus* by the presence of a distinct lateral crest on the descending postorbital process, a squamosal that laterally overlaps the postorbital in its rostral extent, and a rectangular parietal that is excluded from the caudal margin of the supratemporal foramen by the squamosal. The mandibular symphysis of *K*. *langenbergensis* extends over the eight most rostral dentary alveoli and is therefore longer than in all other species of *Theriosuchus*. Finally, the overall shape of the angular and surangular in *K*. *langenbergensis* is similar to other taxa of *Theriosuchus*, but the relative height of the angular is only half of that of all species of *Theriosuchus*. Differences that occur between *Knoetschkesuchus langenbergensis* and individual species of *Theriosuchus* are described below.

Comparison with *Theriosuchus pusillus* [16, 26, 27, 64, 65, 71] (Fig 11): *Knoetschkesuchus langenbergensis* is separated from *T*. *pusillus* by the absence of an extensive lacrimonasal contact, by a frontal margin not elevated at the orbits, by dorsally and ventrally transversely expanded prefrontal pillars, by a medial margin of the supratemporal foramina that is not elevated from the cranial table, by the absence of a crest at the squamosal-parietal suture, by a shorter and more caudally positioned laterally beveled portion at the squamosal, by its more laterally elongated pterygoid wings with a caudolaterally directed and straight lateral margin (Fig 11B and 11C), by the lack of the distinct caudal rim on the pterygoid bordering the choanae caudally, by a capitate process of the laterosphenoid that is laterally oriented, by the presence of a median crest on the basioccipital ventral to the basioccipital condyle, by the absence of low-crowned and serrated teeth, and by the presence of an external mandibular fenestra. *T*. *pusillus* has a comparably smaller interorbital width of only one fifth of the total skull width [78], whereas *K*. *langenbergensis* has a frontal that is at least one third of the total skull width.

Comparison with *Theriosuchus symphiestodon [57, 68]: Knoetschkesuchus langenbergensis* differs from *T*. *symphiestodon* by a relatively longer maxillary body with a sinusoidal ventral maxillary edge in lateral view, and by the presence of an antorbital foramen. The skull reconstruction of *T*. *symphiestodon* shows a more vertically oriented maxillary rostrum that makes the rostral part of the skull higher [57, 68], which is different to *K*. *langenbergensis* that possesses a slightly dorsomedially curved maxilla contributing to a platyrostral condition of the skull. Although only parts of the cranial table are preserved in *T*. *symphiestodon*, the intertemporal width of the parietal in this taxon [68] seems to be larger than in *K*. *langenbergensis*. The frontal of *T*. *symphiestodon* is laterally more expanded and therefore wider than *K*. *langenbergensis* [68], but due to the incomplete rostrum, a comparison of its width to the total skull width cannot be made.

Comparison with *Theriosuchus grandinaris* [53]: *Knoetschkesuchus langenbergensis* can be separated from *T*. *grandinaris* by a lateromedially more expanded maxilla, the presence of an antorbital fenestra, the absence of a lacrimonasal contact, a more lateromedially expanded lacrimal and a longer mandibular symphysis.

Comparison with *Theriosuchus ibericus* [67, 80]: *Theriosuchus ibericus* is described in detail in another work (Tennant et al. pers comm) and therefore will be treated here only very shortly. Obvious differences that allow a distinction between *Knoetschkesuchus langenbergensis* and *T*. *ibericus* are the lack both of a swelling over the enlarged fifth maxillary tooth and a depression on the lateral face of the maxilla caudal to the fifth maxillary tooth in *K*. *langenbergensis*, and a longer caudal process of the jugal of *K*. *langenbergensis*.

Comparison with *Theriosuchus guimarotae* [61] (in this work *Knoetschkesuchus guimarotae* new combination) (Fig 10): *Knoetschkesuchus langenbergensis* differs from *K*. *guimarotae* by its rostromedially directed premaxillomaxillary suture, an essentially rounded triangular prefrontal, a roughly squarish parietal, and, if assignment of osteoderms in both taxa is correct, potentially only weakly keeled paravertebral osteoderms. In contrast to the difference that allow a specific distinction between *K*. *langenbergensis* and *K*. *guimarotae*, there exist several diagnostic features that unite both taxa but separate the unit of these two from other species of *Theriosuchus*:

1. The dentition in both taxa comprises only two tooth morphotypes, including both conical and pseudocaniniform, as well as rounded and more blunt “lanceolate” teeth with striations. In contrast to *T*. *pusillus*, *T*. *symphiestodon*, *T*. *grandinaris*, and *T*. *ibericus*, labiolingually strongly compressed, lower-crowned teeth with serrations are absent in both taxa, independent of the ontogenetic stage.
2. The secondary choanae both in *Knoetschkesuchus guimarotae* and *K*. *langenbergensis* are slit-like foramina on the surface of the pterygoid with only a small groove around it, but are not embedded in a bowl-shaped depression. In contrast, in *T*. *pusillus*, *T*. *symphiestodon*, and in *T*. *ibericus*, the secondary choanae are set in a bowl-like depression caudally bordered by a rounded rim (Figs 10D, 11B and 11C).
3. The laterally beveled area of the squamosal both in *Knoetschkesuchus langenbergensis* and K. *guimarotae* has the same length and is restricted to the caudal third of the squamosal. Additionally, the caudolateral corner of the squamosal of specimen DFMMh/FV 200 and DFMMh/FV 605 forms a rounded, caudally projecting process. The faint sculpturing on the dorsal surface of this process on specimen DFMMh/FV 605 is comparable to that seen on the cranial table. This feature is also present in *K*. *guimarotae*. In contrast, the caudolateral corner of the squamosal forms an unsculptured, caudolaterally directed, knob-like process that overhangs the occiput slightly in *T*. *pusillus* and in *T*. *ibericus* [61], as well as in *T*. *symphiestodon* [57, 68].
4. Proportional similarities in the cranial table of *Knoetschkesuchus langenbergensis* and *K*. *guimarotae* are apparent. The minimum space between the supratemporal foramina comprises one third of the total width of the cranial table both in *K*. *guimarotae* [61] and specimen DFMMh/FV 200 of *K*. *langenbergensis*, but only one sixth of the total width of the cranial table in *T*. *pusillus*. These proportional differences are only partly the result of different ontogenetic stages, as they are apparent in all of these stages (see also above, “Ontogeny”, and see remarks about reconstruction of *Knoetschkesuchus guimarotae*).
5. A proportional similarity in the orbital region of DFMMh/FV 200 of *Knoetschkesuchus langenbergensis* and *K*. *guimarotae* is that the minimum width of the frontal between the orbits comprises one third of the total skull width in both taxa. Ontogenetic proportional changes are demonstrated here in DFMMh/FV 605, which has an interorbital width of half of the total skull width (see above, “Ontogeny”).
6. Both *Knoetschkesuchus langenbergensis* and *K*. *guimarotae* possess an antorbital fenestra, and an external mandibular fenestra, which is developed in all different ontogenetic stages.
7. In *Knoetschkesuchus langenbergensis* and in *K*. *guimarotae*, the lacrimal is excluded from nasal contact by the prefrontal (in smaller specimens), or the lacrimonasal contact is restricted to a small rostromedial tip of the lacrimal (in larger specimens) (Fig 10E and 10F).

These seven diagnostic characters uniting *Knoetschkesuchus guimarotae* and K. *langenbergensis* set both taxa apart from all other species of *Theriosuchus*. The unit of *K*. *langenbergensis* and *K*. *guimarotae* demonstrate the generic differences between *Theriosuchus* and *Knoetschkesuchus*. It has already been mentioned in Young et al. [78] that monophyly of the genus *Theriosuchus* is uncertain and the generic diagnosis is lined with a number of exceptions. If *Theriosuchus pusillus* as the type species of *Theriosuchus* is taken to represent the most characteristic features of the genus, then there are so many differences between *Theriosuchus* and *Knoetschkesuchus* that a generic separation is apparent. In particular basing on the choanal configuration, tooth morphology, proportional differences and the absence of an antorbital foramen, *T*. *pusillus* is closer to *T*. *symphiestodon*, *T*. *grandinaris* and possibly also *T*. *ibericus* (but see Tennant et al. in review), whereas *K*. *langenbergensis* is closer to *K*. *guimarotae* in this respect. These osteological findings strongly indicate generic separation between a group consisting of *K*. *langenbergensis* and *K*. *guimarotae* and another group containing *Theriosuchus* spp. with particular reference to the type species *T*. *pusillus*. We also postulate that *Theriosuchus guimarotae* should be transferred into *Knoetschkesuchus guimarotae* instead. Our finding is supported by the phylogenetic analysis (see above, Fig 9) and can be set in context with biogeographic and ecological implications (see below).

Because *Knoetschkesuchus* cannot be attributed to *Theriosuchus*, similar characters in the diagnosis of Young et al. [78] are possibly not autapomorphic for *Theriosuchus* and need to be removed from the diagnosis of this taxon. In perspective, only a thorough re-description of the lecto- and paratype of *Theriosuchus pusillus* would help to identify truly autapomorphic characters for *Theriosuchus*, and it might well be that the number of taxa referred to this genus is reduced furthermore. The Mid-Jurassic dentary remains of *Theriosuchus* sp. from Scotland [78] does not preserve teeth or the choanae and is therefore difficult to diagnose. However, if the potential autapomorphic characters for *Theriosuchus* given by Young et al. [78] are more widespread among atoposaurids, attribution of this fossil to *Theriosuchus* might be warranted.

### Relationships to other atoposaurids

*Knoetschkesuchus* shows more similarities to *Theriosuchus* than to the other European atoposaurids *Alligatorellus*, *Alligatorium*, *Atoposaurus* [15, 83], and *Montsecosuchus* [18]. Except *Montsecosuchus* from the Early Cretaceous of Spain, all of these taxa derive from Upper Jurassic deposits of Southwestern Germany and France [9, 15, 83], and therefore existed contemporaneously with *Knoetschkesuchus*. A number of ontogenetically influenced features in the skull of atoposaurids make it difficult to identify reliable taxonomic characters in Atoposauridae, but the group is generally characterized by small body size of not exceeding one meter in length, a brevirostrine skull, and orbits at least twice as large as the supratemporal fenestrae [15, 17, 18, 28, 61, 73, 78, 83, 84]. In the past, it has been suggested that the specimens of *Atoposaurus* might represent juvenile individuals of the sympatric atoposaurids *Alligatorium* and *Alligatorellus* [17], or that all three taxa belong to the ontogenetic growth series of a single taxon [16, 28]. In contrast, a recent Principal Component Analysis (PCA) of skull measurements and additional anatomical evidence have demonstrated that *Atoposaurus*, *Alligatorium*, and *Alligatorellus* can be treated as separate taxa [83]. Some new, important osteoderm-based characters [83] cannot be used for taxonomic discussions of atoposaurids here, because they are not preserved in the osteoderms of *Knoetschkesuchus*.

According to the revised diagnosis of *Alligatorellus* [83], *Knoetschkesuchus* differs from *Alligatorellus* in its sculptured rostral part of the skull and sculptured external surface of the dentary. In contrast to *Alligatorellus*, *Knoetschkesuchus* possesses an enlarged fifth maxillary tooth, two tooth-morphs in the upper and lower jaws, a narrow wedge-like rostral frontal process, raised orbital rims, perforated supratemporal foramina, and a rostral process of the squamosal restricted to the skull roof (i.e., not reaching the orbital margin).

*Knoetschkesuchus* differs from *Atoposaurus* by possessing a sculptured skull (smooth/unsculptured in *Atoposaurus*) and palpebral bones (absent in *Atoposaurus*) [83]. Additionally, *Knoetschkesuchus* possesses osteoderms, the absence of which in *Atoposaurus* is considered to be diagnostic and not due to an ontogenetically young developmental stage of the specimens [83]. According to Wellnhofer [15], *Knoetschkesuchus* differs from *Atoposaurus* also in the presence of well-developed supratemporal foramina.

*Alligatorium* is distinguished from *Alligatorellus* and *Atoposaurus* based on osteoderm morphology by Tennant and Mannion [83], which is not preserved in this detail in *Knoetschkesuchus*. *Knoetschkesuchus* differs from *Montsecosuchus* in its caudoventrally directed retroarticular process of the mandible, and absence of a domed or “dolichocephalous” occiput, and in the length-to-width-proportions of the skull [15, 18].

### Distribution of *Knoetschkesuchus* in the Jurassic and ecological implications

During the last years, fossil finds and localities yielding bone material and/or isolated teeth attributed to *Theriosuchus* have increased [see overviews in 61, 68, 78, 83]. The type species *Theriosuchus pusillus* comes from the Lower Cretaceous (Berriasian) Purbeck Formation [26, 27, 64, 65, 71]. Most finds are concentrated to the Upper Jurassic and Lower Cretaceous of West-Central Europe, where *Theriosuchus* represents a typical member of mid- to late Mesozoic crocodyliform communities [79], but remains of *Theriosuchus* are also known from the Upper Cretaceous of West and East Europe [68, 85]. Rare, and partially ambiguous reports of *Theriosuchus* are known from the Upper Jurassic and Lower Cretaceous of Eastern Asia [53, 70, 86], the Mid-Jurassic of Madagascar [87],and the Lower Cretaceous of North America [88]. A recent find of a dentary of *Theriosuchus* sp. from the Middle Jurassic (Bajocian-Bathonian) of Scotland [78] represents the geologically oldest fossil evidence of *Theriosuchus* (but see remark above about the putative problems with the determination of this specimen). *Knoetschkesuchus langenbergensis* from the Langenberg Quarry in German and *K*. *guimarotae* from the Guimarota coalmine in Portugal [61, 81] are contemporaneous taxa from the late Jurassic. Their generic separation from *Theriosuchus* reduces the biogeographic diversity of *Theriosuchus*, and adds to the overall diversity of Late Jurassic crocodyliform paleocommunities.

The Guimarota locality in Leiria, Portugal lies at the eastern margin of the Lusitanian Basin and is an abandoned coal mine with sediments belonging to the Alcobaça-Formation [89]. A diverse crocodyliform community has been described, comprising partial skeletons and isolated dental and skeletal elements of *Machimosaurus hugii* [81, 90, 91], *Bernissartia* sp.[80], *Lisboasaurus estesi* [92], *Goniopholis baryglyphaeus* [93], *Lusitanisuchus mitracostatus* [94] and *Knoetschkesuchus guimarotae* (new combination) [61]. The Guimarota locality represents a lagoonal environment with occasional freshwater influx and periodical flooding by salt water. The area can be imagined as a swamp like region, comparable superficially with recent Mangrove forests [89]. The Guimarota crocodyliforms had a large variety of different prey: *Machimosaurus hugii* was a marine crocodyliform that probably cam only to the coast for egg laying and fed on fish and marine reptiles such as marine turtles [81]. *Goniopholis baryglyphaeus* was a large, amphibious and generalistic taxon that preyed on shelly and also soft prey items feasible for its size, comparable to extant *Alligator mississippiensis* [93]. The small (about 0.5 m in length) crocodyliforms *Lisboasaurus estesi* [92] and *Lusitanisuchus mitracostatus* [94] had a diet of overally small prey items, most probably different kinds of insects. *K*. *guimarotae* is interpreted to feed on insects and small vertebrates, with a similar spectrum to juvenile extant crocodylians [61, 80].

The Langenberg Quarry belongs to the southern part of the Lower Saxony Basin, which represents shallow marine conditions and was interspersed with small islands. The sediments in the Langenberg Quarry seem to have occasional freshwater influx, and salinity levels changed. The crocodyliform assemblage from the Langenberg Quarry comprises besides *Knoetschkesuchus langenbergensis* also partial skeletons of *Goniopholis*, *Machimosaurus*, and *Steneosaurus* [6–8], it is therefore less diverse than that of Guimarota and contains allochthonous and autochtonous elements. Whereas the marine crocodyliforms *Machimosaurus* and *Steneosaurus* would have fed mainly on fish and marine turtles, the feeding preferences of *Goniopholis* and *Knoetschkesuchus* are comparable with the same taxa from Guimarota. It should be noted that the Langenberg Quarry is probably an allochthonous deposit, into which animals and plants from the nearby islands were transported (see section “Locality”), so that the quarry itself does not represent the genuine habitat of *T*. *langenbergensis*.

During the Late Jurassic and also the earliest Cretaceous, Europe was covered largely by shallow epicontinental seas, with interspersed continental islands, such as the Iberian Meseta and the Amorican Massif [95]. North America and Eurasia were separating by ongoing crustal extension that started already in the Late Triassic and initiated subsidence of the Lusitanian Basin [96]. The high Jurassic and Cretaceous diversity of neosuchian crocodyliforms is attributed to paleogeography with a system of island archipelagos and periodic sea level changes [39, 97, 98], which, for example, could have facilitated allopatric speciation of different atoposaurids [83]. *Knoetschkesuchus guimarotae* from the Lusitanian basin inhabited an area at the margin of the Iberian Meseta, whereas *K*. *langenbergensis* is associated with a landmass comprising the Rhenisch Massif, Bohemian Massiv, and London-Brabant Massif [39]. Biogeographically, dispersal between the two localities in the Late Jurassic would have been possible for *Knoetschkesuchus*, chosing routes along larger landmasses in the marine basins such as the Amorican Massif. This mechanism would be similar to a postulated faunal interchange between the Early Cretaceous terrestrial fauna in the Lower Saxon Basin and the Wesses-Weald Basin [99]. It is equally plausible that the epicontinental sea provided an insurmoutable geographic barrier for such small sized taxa, separating species from each other.

Progressive uplift of the basin margins in the latest Jurassic caused a change from lagoonal environments like Guimarota and the later carbonate dominated “Purbeck” facies to fluvial clastics as found in the “Wealden” facies [96, 97]. This could have triggered the evolution of differences in the dentition between the closely related taxa *Knoetschkesuchus*, and *Theriosuchus*. For example, the tripartite tooth morphology of *Theriosuchus* has been compared to the dentition of extant iguanids [67, 100], comprising a mainly entomophagous diet. By comparison with *Iguana iguana*, Brinkmann [67] assumed that *Theriosuchus* might have equally likely had a herbivorous component in its diet, which was corroborated by the study of Ösi [101]. It has also been suggested by Young et al. 2015 [78] that the different tooth morphologies in atoposaurids might be indicative for dietary specializations. It is possible that in concert with the changing paleoenvironment, the development of a specialized dentition in *Theriosuchus* would have allowed the animals to respond to the environmental changes and to realize particular niches different to those realized by *Knoetschkesuchus*. In particular the type species *Theriosuchus pusillus* derives from an extremely diverse crocodyliform paleocommunity [26, 27], where specialization to a certain type of well available food such as insects, or the addition of plant material into the diet might have been an ecological advantage. The potential niche partitioning in the Early Cretaceous crocodyliform community of Western Europe suggested by Tennant & Mannion [83] is supported by our finds. The morphological diversity of dentition was described to be a potential driver of the evolution of atoposaurids in the Middle to Late Jurassic by Young et al. [78] and would be consistent with the ecological, biogeographical and taxonomical variation of taxa hitherto associated with *Theriosuchus* and *Knoetschkesuchus* as described herein.

## Supporting information

S1 Table Measurements of *Knoetschkesuchus langenbergensis* gen. nov. sp. nov. (DOCX)

S1 Text Character list, taxon list, and documented changes in the matrix. This file includes character list, taxon list, and documented changes in the matrix used for the phylogenetic analysis.
(DOCX)

S1 Matrix Matrix used for the phylogenetic analysis. (TXT)

S1 Fig Consensus tree with Bremer support values. The consensus tree is recovered from 18 most parsimonious trees of a matrix of 82 ingroup taxa and 321 phenotypic characters.
(TIF)

S2 Fig Standard bootstrap and jackknife support analysis. Trees obtained from the character matrix (excluding *Elosuchus cherifiensis*). Tree topologiesüöä_obtained both from jackknifing and bootstrapping are summarized using GC frequencies.
(TIF)
