# Supplementary material for: Knoetschkesuchus langenbergensis gen. nov. sp. nov., a new atoposaurid crocodyliform from the Upper Jurassic Langenberg Quarry (Lower Saxony, northwestern Germany), and its relationships to Theriosuchus
Source: PLoS One. 2017 Feb 15;12(2):e0160617. doi: 10.1371/journal.pone.0160617 (PMC5310792; doi:10.1371/journal.pone.0160617)
Supplement: S1 Table — (DOCX) [file pone.0160617.s001.docx]

**S1 Table. Measurements of *Knoetschkesuchus langenbergensis*, all specimens, all given in mm.**

|  | **DFMMh/FV 200; skull** | **DFMMh/FV 605; skull** | **DFMMh/ FV 790.12; dentary** | **DFMMh/FV 261; angular** | **DFMMh/FV 325; slab with bones** | **DFMMh/FV 279; femur** | **DFMMh/FV 790.11; metatarsal** |
| --- | --- | --- | --- | --- | --- | --- | --- |
| **Skull** |  |  |  |  |  |  |  |
| **Total length** | 450 | 300 |  |  |  |  |  |
| **Caudal-most cranium width** | 144 | 191 |  |  |  |  |  |
| **Width at caudal orbital margin** | 113 | 101 |  |  |  |  |  |
| **Width at rostral orbital margin** | 95 | 48 |  |  |  |  |  |
| **Width caudal to external naris** | 48 | 43 |  |  |  |  |  |
| **Length of cranial table** | 90 |  |  |  |  |  |  |
| **Minimum width of cranial table** | 86 |  |  |  |  |  |  |
| **Maximum width of cranial table** | 130 |  |  |  |  |  |  |
| **Orbital length** | 147 | 84 |  |  |  |  |  |
| **Orbital width** | 62 | 52 |  |  |  |  |  |
| **Length of supratemporal foramen** | 54 | 46 |  |  |  |  |  |
| **Width of supratemporal foramen** | 23 | 25 |  |  |  |  |  |
| **Length of infratemporal fenestra** | 63 |  |  |  |  |  |  |
| **Width of infratemporal fenestra** | 37 |  |  |  |  |  |  |
| **Length of frontal** | 154 |  |  |  |  |  |  |
| **Minimum width of frontal between orbits** | 32 | 35 |  |  |  |  |  |
| **Length of jugal** | 175 | 133 |  |  |  |  |  |
| **Minimum height of jugal caudal to dorsally ascending process** | 8 | 6 |  |  |  |  |  |
| **Maximum height of jugal rostral to dorsally ascending process** | 18 | 9 |  |  |  |  |  |
| **Length of quadratojugal** | 34 |  |  |  |  |  |  |
| **Length of parietal** | 94 | 67 |  |  |  |  |  |
| **Minimum width of parietal (rostral margin)** | 48 | 45 |  |  |  |  |  |
| **Maximum width of parietal (caudal margin)** | 57 | 51 |  |  |  |  |  |
| **Maximum length of squamosal** | 93 | 42 |  |  |  |  |  |
| **Width of squamosal (rostral portion)** | 9 | 11 |  |  |  |  |  |
| **Width of squamosal (caudal margin)** | 35 | 31 |  |  |  |  |  |
| **Length of nasal** | 138 | 60 |  |  |  |  |  |
| **Minimum width of nasal** | 11 | 22 |  |  |  |  |  |
| **Maximum width of nasal** | 55 | 42 |  |  |  |  |  |
| **Length of premaxilla** | 62 | 27 |  |  |  |  |  |
| **Width of premaxilla** | 29 | 16 |  |  |  |  |  |
| **Length of maxilla** | 152 | 93 |  |  |  |  |  |
| **Minimum height of maxilla** | 13 | 4 |  |  |  |  |  |
| **Maximum height of maxilla** | 46 | 30 |  |  |  |  |  |
| **Length of palpebral** | 67 | 56 |  |  |  |  |  |
| **Width of palpebral** | 22 | 18 |  |  |  |  |  |
| **Length of prefrontal** | 66 | 25 |  |  |  |  |  |
| **Height of prefrontal** | 14 |  |  |  |  |  |  |
| **Width of lacrimal** | 18 |  |  |  |  |  |  |
| **Length of antorbital foramen** |  | 8 |  |  |  |  |  |
| **Length of frontal** |  | 89 |  |  |  |  |  |
| **Width of frontal (interorbital width)** |  | 37 |  |  |  |  |  |
| **Width of pterygoid** |  | 100 |  |  |  |  |  |
| **Length of pterygoid** |  | 38 |  |  |  |  |  |
| **Width of exoccipital** |  | 44 |  |  |  |  |  |
| **Height of supraoccipital** |  | 13 |  |  |  |  |  |
| **Width of supraoccipital** |  | 24 |  |  |  |  |  |
| **Height of exoccipital** |  | 58 |  |  |  |  |  |
| **Width of exoccipital** |  | 44 |  |  |  |  |  |
|  |  |  |  |  |  |  |  |
| **Mandible** |  |  |  |  |  |  |  |
| **Length of dentary** | 298 | 203 | 270 |  |  |  |  |
| **Height of dentary** | 25 | 13 | 24 |  |  |  |  |
| **Height of dentary** | 31 | 26 | 32 |  |  |  |  |
| **Length of splenial** |  | 98 |  |  |  |  |  |
| **Length of angular** | 173 | 61 |  | 486 |  |  |  |
| **Height of angular (rostral-most tip)** | 6 | 9 |  | 31 |  |  |  |
| **Height of angular** | 37 | 20 |  | 95 |  |  |  |
| **Length of surangular** | 174 | 174 |  |  |  |  |  |
|  |  |  |  |  |  |  |  |
| **Postcranial bones** |  |  |  |  |  |  |  |
| **Vertebra: Length of body** | 40 |  |  |  |  |  |  |
| **Vertebra: Width of vertebral body** | 32 |  |  |  |  |  |  |
| **Scapula length** | 173 |  |  |  |  |  |  |
| **Humerus length** | 253 |  |  |  |  |  |  |
| **Metacarpal length** | 50 |  |  |  |  |  |  |
| **Metatarsal length** | 63 |  |  |  |  |  | 95 |
| **Thoracic rib 1 length** | 106 |  |  |  | 74 |  |  |
| **Thoracic rib 2 length** | 96 |  |  |  |  |  |  |
| **Thoracic rib 3 length** | 57 |  |  |  |  |  |  |
| **Ischium length** | 97 |  |  |  |  |  |  |
| **Femur length** |  |  |  |  |  | 210 |  |
| **Tibia length** | 195 |  |  |  |  |  |  |
| **Fibula length** | 177 |  |  |  |  |  |  |
| **Length of osteoderm 1** | 40 |  |  |  | 40 |  |  |
| **Width of osteoderm 1** | 30 |  |  |  | 30 |  |  |
| **Length of osteoderm 2** | 40 |  |  |  | 30 |  |  |
| **Width of osteoderm 2** | 30 |  |  |  | 20 |  |  |
| **Length of osteoderm 3** | 50 |  |  |  | 30 |  |  |
| **Width of osteoderm 3** | 40 |  |  |  | 20 |  |  |
| **Length of osteoderm 4** | 40 |  |  |  |  |  |  |
| **Width of osteoderm 4** | 30 |  |  |  |  |  |  |
| **Length of osteoderm 5** | 30 |  |  |  |  |  |  |
| **Width of osteoderm 5** | 20 |  |  |  |  |  |  |
