# Supplementary material for: Knoetschkesuchus langenbergensis gen. nov. sp. nov., a new atoposaurid crocodyliform from the Upper Jurassic Langenberg Quarry (Lower Saxony, northwestern Germany), and its relationships to Theriosuchus
Source: PLoS One. 2017 Feb 15;12(2):e0160617. doi: 10.1371/journal.pone.0160617 (PMC5310792; doi:10.1371/journal.pone.0160617)
Supplement: S1 Text — This file includes character list, taxon list, and documented changes in the matrix used for the phylogenetic analysis. (DOCX) [file pone.0160617.s002.docx]

**Phylogenetic analysis – Character list**

The character matrix of Turner (2015) was used for the phylogenetic analysis, because this author provides the most recent comprehensive and detailed coding for different species of *Theriosuchus*. Several modifications both in the matrix as in the coding became necessary due to the purpose of this paper and are outlined below.

The character list from Turner (2015) comprises 321 phenotypical characters and allows a maximum of characters to be coded for the specimen of *Knoetschkesuchus langenbergensis* gen. nov. sp. nov. Changes in the wording and remarks on the characters are documented with footnotes in the following list. The character list of Turner (2015) is adapted from Turner & Sertich (2010), which itself is based on Turner and Buckley (2008), Pol et al. (2009), Pol and Norell (2004a, 2004b) and Pol and Apesteguía (2005). The list includes also characters from Turner (2006), Brochu (1997), Pritchard et al. (2013) and Adams (2013). Character definitions 1–101 were taken and modified from Clark (1994) with the same numeration as in the original publication.

Characters 1, 3, 6, 10, 23, 37, 43-45, 49, 65, 67, 69, 73, 77, 79, 86, 90, 91, 96, 97, 104-106, 108, 126, 140, 142, 143, 149, 167, 182, 197, 227 were set as additive by Turner (2015), representing potentially nested sets of homologies and/or entail presence and absence information. Turner (2015) omitted character 5 (due to its interdependence with character 6), character 277 (due to doubts of the homology coded for by this character) and character 281 (due to its redundancy with character 25). Deviating from this procedure, in our analysis, we used the full character set and did not set characters as additive or exclude them.

**Character 1** (Turner 2015, modified from Clark, 1994: char. 1): External surface of dorsal cranial bones: smooth (0), slightly grooved (1), and heavily ornamented with deep pits and grooves (2).

**Character 2** (Turner 2015, modified from Clark, 1994: char. 2): Skull expansion at orbits: gradual (0), or abrupt (1).

**Character 3** (Turner 2015, modified from Clark, 1994: char. 3): Rostrum proportions: narrow oreinirostral (0), broad oreinirostral(1), nearly tubular (2), or platyrostral (3).

**Character 4** (Clark, 1994: char. 4): Premaxilla participation in internarial bar: forming at least ventral half (0), or with little participation (1).

**Character 5** (Clark, 1994: char. 5): Premaxilla anterior to nares: narrow (0), or broad (1).

**Character 6** (Turner 2015, modified from Clark, 1994: char. 6): External nares facing anterolaterally or anteriorly (0); dorsally, not separated by premaxillary bar from anterior edge of rostrum (1); or dorsally, separated by premaxillary bar (2).

**Character 7** (Clark, 1994: char. 7): Palatal parts of premaxillae: do not meet posterior to incisive foramen (0), or meet posteriorly along contact with maxillae (1).

**Character 8** (Clark, 1994: char. 8): Premaxilla-maxilla contact: premaxilla loosely overlies maxilla (i.e., posterodorsal process of premaxilla overlaps anterodorsal surface of maxilla)(0), or sutured together along butt joint (1).

**Character 9** (Turner 2015, modified from Clark, 1994: char. 9): Ventrally opened notch on ventral edge of rostrum at premaxilla-maxilla contact: absent (0), present as notch (1), or present as large fenestra (2).

**Character 10** (Turner 2015, modified from Clark, 1994: char. 10 by Pol et al., 2009): Posterior palatal branches of maxillae anterior to palatines: do not meet (0), meet extensively but posteriormost parts fail to meet (1), or meet entirely (2).

**Character 11** (Clark, 1994: char. 11): Nasal contacts lacrimal (0), or does not contact lacrimal (1).

**Character 12** (Clark, 1994: char. 12): Lacrimal contacts nasal along medial edge only (0), or along medial and anterior edges (1).^[[1]](#footnote-1)^

**Character 13** (Clark, 1994: char. 13): Nasal contribution to narial border: present (0), or absent (1).

**Character 14** (Clark, 1994: char. 14): Nasal-premaxilla contact: present (0), or absent (1).

**Character 15** (Turner 2015, modified from Clark, 1994: char. 15): Descending process of prefrontal: does not contact palate (0), or contacts palate (1).

**Character 16** (Clark, 1994: char. 16): Postorbital-jugal contact: postorbital anterior to jugal (0), postorbital medial to jugal (1), or postorbital lateral to jugal (2).

**Character 17** (Clark, 1994: char. 17): Anterior part of jugal with respect to posterior part: as broad (0), or twice as broad (1).

**Character 18** (Clark, 1994: char. 18): Jugal bar beneath infratemporal fenestra: dorsolaterally flattened (0), or rod-shaped (1).

**Character 19** (Clark, 1994: char. 19): Quadratojugal dorsal process: narrow, contacting only small part of postorbital (0); or broad, extensively contacting postorbital (1).

**Character 20** (Clark, 1994: char. 20): Frontal width between orbits: narrow, as broad as nasals (0); or broad, twice as broad as nasals (1).

**Character 21** (Clark, 1994: char. 21): Frontals in mature specimens: paired (0), or unpaired (1).^[[2]](#footnote-2)^

**Character 22** (Clark, 1994: char. 22): Dorsal surface of frontal and parietal: flat (0), or with midline ridge (1).

**Character 23** (Turner 2015, modified from Clark, 1994: char. 23 by Buckley and Brochu, 1999: char. 81): Parieto-postorbital suture: absent from dorsal surface of skull roof and supratemporal fossa (0), absent from dorsal surface of skull roof but broadly present within supratemporal fossa (1), or present within supratemporal fossa and on dorsal surface of skull roof (2).

**Character 24** (Clark, 1994: char. 24): Dorsal surface of supratemporal roof: complex (0); or dorsally flat ‘skull table’ developed, with postorbital and squamosal bearing flat shelves extending laterally beyond quadrate contact (1).

**Character 25** (Turner 2015, modified from Clark, 1994: char. 25): Postorbital bar: sculpted (if skull sculpted) (0), or unsculpted (1).

**Character 26** (Turner 2015, modified from Clark, 1994: char. 26): Postorbital bar: transversely flattened (0), or cylindrical (1).

**Character 27** (Clark, 1994: char. 27): Vascular opening in dorsal surface of postorbital bar: absent (0), or present (1).

**Character 28** (Turner 2015, modified from Clark, 1994: char. 28): Postorbital anterolateral process: absent or poorly developed (0); or well developed, long, and acute (1).

**Character 29** (Clark, 1994: char. 29): Dorsal part of postorbital: with anterior and lateral edges only (0), or with anterolaterally facing edge (1).

**Character 30** (Clark, 1994: char. 30): Dorsal end of postorbital bar broadens dorsally, continuous with dorsal part of postorbital (0); or dorsal part of postorbital bar constricted, distinct from dorsal part of postorbital (1).

**Character 31** (Clark, 1994: char. 31): Bar between orbit and supratemporal fossa in mature specimens: broad and solid, with broadly sculpted dorsal surface if sculpture present (0); or bar narrow, sculpting restricted to anterior surface (1).^[[3]](#footnote-3)^

**Character 32** (modified from Clark, 1994: char. 32): Parietal: with broad occipital portion (0), or without broad occipital portion (1).

**Character 33** (Clark, 1994: char. 33) Parietal: with broad sculpted region separating supratemporal fossae (0), or with sagittal crest between supratemporal fossae (1).

**Character 34** (Clark, 1994: char. 34): Postparietal (dermosupraoccipital): a distinct element (0), or not distinct (fused with parietal?) (1).

**Character 35** (Clark, 1994: char. 35): Posterodorsal corner of squamosal: squared off, lacking extra ‘lobe’ (0); or with unsculptured ‘lobe’ (1).

**Character 36** (Turner 2015, modified from Clark, 1994: char. 36): Posterolateral process of squamosal: poorly developed and projecting horizontally at same level of skull (0); elongate, thin, and posteriorly directed, not ventrally deflected (1); or elongate, posterolaterally directed, and ventrally deflected (2).

**Character 37** (Clark, 1994: char. 37): Palatines: do not meet on palate below narial passage (0); form palatal shelves that do not meet (1); or meet ventral to narial passage, forming part of secondary palate (2).

**Character 38** (Clark, 1994: char. 38): Pterygoid: restricted to palate and suspensorium, joints with quadrate and basisphenoid overlapping (0); or extends dorsally to contact laterosphenoid and form ventrolateral edge of trigeminal foramen, strongly sutured to quadrate and laterosphenoid (1).

**Character 39** (Turner 2015, modified from Clark, 1994: char. 39): Choanal opening: continuous with pterygoid ventral surface except for anterior and anterolateral borders (0), or opens into palate through deep midline depression (choanal groove) (1).

**Character 40** (Clark, 1994: char. 40): Palatal surface of pterygoids: smooth (0), or sculpted (1).

**Character 41** (Clark, 1994: char. 41): Pterygoids posterior to choanae: separated (0), or fused (1).

**Character 42** (Turner 2015, modified from Clark, 1994: char. 42, Ortega et al., 2000: char. 139): Depression on primary pterygoidean palate posterior to choana: absent or moderate in size, being narrower than palatine bar (0), or wider than palatine bar (1).

**Character 43** (Turner 2015, modified from Clark, 1994: char. 43): Primary pterygoidean palate, role in forming choanal opening: does not enclose choana (0), completely encloses choana (1).

**Character 44** (Turner 2015, modified from Pol and Norell, 2004a, by Pol et al., 2009; modified from Clark, 1994: char. 44): Anterior edge of choanae situated between suborbital fenestra (or anteriorly) (0), situated near the posterior edge of suborbital fenestra (1), or near posterior edge of pterygoid flange (2).

**Character 45** (Clark, 1994: char. 45): Quadrate: without fenestrae (0), with single fenestra (1), or with three or more fenestrae on dorsal and posteromedial surfaces (2).

**Character 46** (Clark, 1994: char. 46): Posterior edge of quadrate: broad medial to tympanum, gently concave (0); or posterior edge narrow dorsal to otoccipital contact, strongly concave (1).

**Character 47** (Clark, 1994: char. 47): Dorsal, primary head of quadrate articulates with: squamosal, otoccipital, and prootic (0); or prootic and laterosphenoid (1).

**Character 48** (Clark, 1994: char. 48): Ventrolateral contact of otoccipital with quadrate: very narrow (0), or broad (1).

**Character 49** (Clark, 1994: char. 49): Quadrate, squamosal, and otoccipital: do not meet to enclose cranioquadrate passage (0), enclose passage near lateral edge of skull (1), or meet broadly lateral to passage (2).

**Character 50** (Clark, 1994: char. 50): Pterygoid ramus of quadrate: with flat ventral edge (0), or with deep groove along ventral edge (1).

**Character 51** (Clark, 1994: char. 51): Ventromedial part of quadrate: does not contact otoccipital (0), or contacts otoccipital to enclose carotid artery and form passage for cranial nerves IX–XI (1).

**Character 52** (Clark, 1994: char. 52): Eustachian tubes: not enclosed between basioccipital and basisphenoid (0), or entirely enclosed (1).

**Character 53** (Clark, 1994: char. 53): Basisphenoid rostrum (cultriform process): slender (0), or dorsoventrally expanded (1).

**Character 54** (Clark, 1994: char. 54): Basipterygoid process: prominent, forming movable joint with pterygoid (0); or small or absent, with basisphenoid joint suturally closed (1).

**Character 55** (Turner 2015, modified from Clark, 1994: char. 55 by Ortega et al., 2000: char. 68): Basisphenoid ventral surface: shorter than basioccipital (0); or wide and similar to, or longer, in length than basioccipital (1).

**Character 56** (Clark, 1994: char. 56): Basisphenoid: exposed on ventral surface of braincase (0), or virtually excluded from ventral surface by pterygoid and basioccipital (1).

**Character 57** (Clark, 1994: char. 57): Basioccipital: without well-developed bilateral tuberosities (0), or with large pendulous tubera (1).

**Character 58** (Clark, 1994: char. 58): Otoccipital: without laterally concave descending flange ventral to subcapsular process (0), or with flange (1).

**Character 59** (Clark, 1994: char. 59): Cranial nerves IX–XI: all pass through common large foramen vagi in otoccipital (0), or cranial nerve IX passes medial to nerves X and XI in separate passage (1).

**Character 60** (Clark, 1994: char. 60): Otoccipital: without large ventrolateral part ventral to paroccipital process (0), or with large ventrolateral part (1).

**Character 61** (Clark, 1994: char. 61): Crista interfenestralis between fenestrae pseudorotunda and ovalis: nearly vertical (0), or horizontal (1).

**Character 62** (Clark, 1994: char. 62): Supraoccipital: forms dorsal edge of foramen magnum (0); or otoccipitals broadly meet dorsal to foramen magnum, separating supraoccipital from foramen magnum (1).

**Character 63** (Clark, 1994: char. 63):Mastoid antrum: does not extend into supraoccipital (0), or extends through transverse canal in supraoccipital to connect middle ear regions (1).

**Character 64** (Clark, 1994: char. 64): Posterior surface of supraoccipital: nearly flat (0), or with bilateral posterior prominences (1).

**Character 65** (Turner 2015, modified from Clark, 1994: char. 65): Palpebrals: absent (0), or one small palpebral present in orbit (1), or one large palpebral (2), or two large palpebrals (3).

**Character 66** (Clark, 1994: char. 66): External nares: divided by septum (0), or confluent (1).

**Character 67** (Clark, 1994: char. 67): Antorbital fenestra: as large as orbit (0), about half the diameter of orbit (1), much smaller than the orbit (2), or absent (3).

**Character 68** (Turner 2015, modified from Clark, 1994: char. 68 by Ortega et al., 2000: char. 41): Supratemporal fenestrae extension: relatively large, covering most of surface of skull roof (0); or relatively short, fenestrae surrounded by flat and extended skull roof (1).

**Character 69** (Turner 2015, modified from Clark, 1994: char. 69): Choanal groove: undivided (0), partially septated (1), or completely septated (2).

**Character 70** (Clark, 1994: char. 70): Dentary: extends posteriorly beneath mandibular fenestra (0), or does not extend beneath fenestra (1).

**Character 71** (Turner 2015, modified from Clark, 1994: char. 71): Retroarticular process: absent or extremely reduced (0); very short, broad, and robust (1); with extensive, rounded, wide, and flat (or slightly concave) surface projecting posteroventrally and facing dorsomedially (2); posteriorly elongate, triangular, and facing dorsally (3); or posteroventrally projecting and paddle-shaped (4).

**Character 72** (Clark, 1994: char. 72): Prearticular: present (0), or absent (1).

**Character 73** (Turner 2015, modified from Clark, 1994: char. 73): Articular: without medial process (0), with short process not contacting braincase (1), or with process articulating with otoccipital and basisphenoid (2).

**Character 74** (Clark, 1994: char. 74): Dorsal edge of surangular: flat (0), or arched dorsally (1).

**Character 75** (Clark, 1994: char. 75): Mandibular fenestra: present (0), or absent (1).

**Character 76** (Clark, 1994: char. 76): Insertion area for M. pterygoideous posterior: does not extend onto lateral surface of angular (0), or extends onto lateral surface of angular (1).

**Character 77** (Turner 2015, modified from Clark, 1994: char. 77): Splenial involvement in mandibular symphysis in ventral view: not involved (0), involved slightly in symphysis (1), or extensively involved (2).

**Character 78** (Turner 2015, modified from Clark, 1994: char. 78): Posterior premaxillary teeth: similar in size to anterior teeth (0), or longer but does not form an enlarged caniniform tooth (1), or much longer forming one large premaxillary caniniform tooth (2), or much longer forming two large premaxillary caniniform teeth (3).

**Character 79** (Turner 2015, modified from Clark, 1994: char. 79): Maxillary tooth waves: absent, no tooth size variation (0); one wave of teeth enlarged (1); or enlarged maxillary teeth occur in two waves (festooned) (2).

**Character 80** (Clark, 1994: char. 80): Anterior dentary teeth opposite premaxilla-maxilla contact: no more than twice the length of other dentary teeth (0), or more than twice the length (1).

**Character 81** (Turner 2015, modified from Clark, 1994: char. 81): Dentary teeth posterior to tooth opposite premaxilla-maxilla contact: equal in size (0), or enlarged dentary teeth opposite to smaller teeth in maxillary toothrow (1).

**Character 82** (Turner 2015, modified from Clark, 1994: char. 82 by Ortega et al., 2000: char. 120): Anterior and posterior scapular edges: symmetrical in lateral view (0), anterior edge more strongly concave than posterior edge (1), or dorsally narrow with straight edges (2).

**Character 83** (Turner 2015, modified from Clark, 1994: char. 83 by Ortega et al., 2000: char. 121): Coracoid length: up to two-thirds of scapular length (0), or subequal in length to scapula (1).

**Character 84** (Clark, 1994: char. 84): Anterior process of ilium: similar in length to posterior process (0), or one-quarter or less length of posterior process (1).

**Character 85** (Clark, 1994: char. 85): Pubis: rod-like without expanded distal end (0), or with expanded distal end (1).

**Character 86** (Clark, 1994: char. 86): Pubis: forms anterior half of ventral edge of acetabulum (0), contacting ilium but partially excluded from acetabulum by anterior process of ischium (1), or completely excluded from acetabulum by anterior process of ischium (2).

**Character 87** (Clark, 1994: char. 87): Distal end of femur: with large lateral facet for fibula (0), or with very small facet (1).^[[4]](#footnote-4)^

**Character 88** (Clark, 1994: char. 88): Fifth pedal digit: with phalanges (0), or without phalanges (1).

**Character 89** (Clark, 1994: char. 89): Atlas intercentrum: broader than long (0), or as long as broad (1).

**Character 90** (Turner 2015, modified from Clark, 1994: char. 90): Cervical neural spines: all anteroposteriorly large (0), only posterior ones rod-like (1), or all spines rod-like (2).

**Character 91** (Turner 2015, modified from Clark, 1994: char. 91; Buscalioni and Sanz, 1988: char. 37; Brochu, 1997a: char. 7): Hypapophyses in cervicodorsal vertebrae: absent (0), present only in cervical vertebrae (1), present in cervical and first two dorsal vertebrae (2), present up to the third dorsal vertebra (3), or up to the fourth dorsal vertebrae (4).

**Character 92** (Clark, 1994: char. 92): Cervical vertebrae: amphicoelous or amphyplatyan (0), or procoelous (1).

**Character 93** (Clark, 1994: char. 93): Trunk vertebrae: amphicoelous or amphyplatyan (0), or procoelous (1).

**Character 94** (Clark, 1994: char. 94): All caudal vertebrae: amphicoelous or amphyplatyan (0), first caudal vertebra biconvex with other procoelous (1), or procoelous (2).

**Character 95** (Turner 2015, modified from Clark, 1994: char. 95 by Turner and Sertich, 2010): Dorsal osteoderms, shape: rounded or ovate (0); rectangular, broader than long (1); square (2); rectangular, longer than broad (3).

**Character 96** (Turner 2015, modified from Clark, 1994: char. 96, and Brochu, 1997a: char. 40): Dorsal osteoderms: without articular anterior process (0), with a discrete convexity on anterior margin (1), or with a well-developed process located anterolaterally in dorsal parasagittal osteoderms (2).

**Character 97** (Turner 2015, modified from Clark, 1994: char. 97 by Ortega et al., 2000: chars. 107 and 108 and this paper): Rows of dorsal primary osteoderms (sensu Frey, 1988): two parallel rows (0), more than two (1), or more than four (2).

**Character 98** (Clark, 1994: char. 98): Osteoderms: some or all imbricated (0), or sutured to one another (1).

**Character 99** (Clark, 1994: char. 99): Tail osteoderms: dorsal only (0), or completely surrounding tail (1). ^[[5]](#footnote-5)^

**Character 100** (Clark, 1994: char. 100): Trunk osteoderms: absent from ventral part of the trunk (0), or present (1).

**Character 101** (Clark, 1994: char. 101): Osteoderms: with longitudinal keels on dorsal surfaces (0), or without longitudinal keels (1).

**Character 102** (Wu and Sues, 1996: char. 14): Jugal: participating in margin of antorbital fossa (0), or separated from it (1).^[[6]](#footnote-6)^

**Character 103** (Turner 2015, modified from Wu and Sues, 1996: char. 17): Mandibular symphysis in lateral view: shallow and tapering anteriorly (0), deep and tapering anteriorly (1), deep and anteriorly convex (2), or shallow and anteriorly convex (3).

**Character 104** (Turner 2015, modified from Wu and Sues, 1996: char. 23): Articular facet for quadrate condyle: equal in length to quadrate condyles (0), slightly longer (1), or close to three times length of quadrate condyles (2).

**Character 105** (Turner 2015, modified from Wu and Sues, 1996: char. 24 and Wu et al., 1997: char. 124): Jaw joint: placed level with occipital condyle (0), below occipital condyle but above level of lower toothrow (1), or below level of toothrow (2).

**Character 106** (modified fromWu and Sues, 1996: char. 27 and Ortega et al., 2000: char. 133): Premaxillary tooth number: six (0), five (1), four (2), three (3), two (4)^[[7]](#footnote-7)^

**Character 107** (Turner 2015, modified from Wu and Sues, 1996: char. 29): Unsculptured region along alveolar margin on lateral surface of maxilla: absent (0), or present (1).

**Character 108** (Wu and Sues, 1996: char. 30): Maxillary tooth number: eight or more (0), seven (1), six (2), five (3), or four (4).

**Character 109** (Wu and Sues, 1996: char. 33): Coracoid, posteromedial or ventromedial process absent (0), elongate posteromedial process present (1), distally expanded ventromedial process present (2).

**Character 110** (Wu and Sues, 1996: char. 40): Radiale and ulnare, size: short and massive (0), elongate (1).

**Character 111** (Turner 2015, modified from Gomani, 1997: char. 4): Prefrontals anterior to orbits: elongate, oriented parallel to anteroposterior axis of skull (0); short and broad, oriented posteromedially-anterolaterally (1).

**Character 112** (Turner 2015, modified from Gomani, 1997: char. 32): Basioccipital and ventral part of otoccipital, orientation: facing posteriorly (0), posteroventrally (1).

**Character 113** (Buscalioni and Sanz, 1988: char. 35): Vertebral centra, shape: cylindrical (0), spool-shaped (1).

**Character 114** (Turner 2015, modified from Buscalioni and Sanz, 1988: char. 39): Transverse process of posterior dorsal vertebrae, shape: dorsoventrally low and laminar (0), dorsoventrally high (1).

**Character 115** (Buscalioni and Sanz, 1988: char. 44): Number of sacral vertebrae: two (0), more than two (1).

**Character 116** (Buscalioni and Sanz, 1988: char. 49): Supraacetabular crest: present (0), absent (1).

**Character 117** (Buscalioni and Sanz, 1988: char. 54): Proximal end of radiale, shape: expanded symmetrically, similarly to distal end (0); more expanded proximolaterally than proximomedially (1).

**Character 118** (Ortega et al., 1996: char. 5): Lateral surface of anterior region of surangular and posterior region of dentary: without longitudinal depression (0), with longitudinal depression (1).

**Character 119** (Ortega et al., 1996: char. 9): Ventral exposure of splenials: absent (0), or present (1).

**Character 120** (Turner 2015, modified from Andrade and Bertini, 2008a: char. 120; Ortega et al., 1996: char. 11; Ortega et al., 2000: char. 100): Tooth margin carinae: without carinae or with smooth or crenulated carinae (0), with homogeneous denticulate carinae (denticles are small and symmetrical in form as in ziphodont teeth) (1), with heterogeneous carinae possessing rounded tubercle-like denticles, developed preferentially along posterior margin (2).

**Character 121** (Turner 2015, modified from Pol, 1999a: char. 133 and Ortega et al., 2000: char. 145): Lateral surface of anterior process of jugal: flat or convex (0), with broad shelf below the orbit with triangular depression underneath it (1).

**Character 122** (Pol, 1999a: char. 134): Jugal, extension below the orbit: does not exceed the anterior margin of orbit (0), exceeds margin of orbit (1).

**Character 123** (Pol, 1999a: char. 135): Notch in premaxilla on lateral edge of external nares: absent (0), or present on the dorsal half of the external nares lateral margin (1).

**Character 124** (Pol, 1999a: char. 136): Dorsal border of external nares: formed mostly by the nasals (0), formed by both the nasals and premaxilla (1).

**Character 125** (Pol, 1999a: char. 138): Posterodorsal process of premaxilla: absent (0), present extending posteriorly and wedging between maxilla and nasal (1).

**Character 126** (Pol, 1999a: char. 139 and Ortega et al., 2000: char. 9): Premaxilla-maxilla suture in palatal view, medial to alveolar region, orientation of suture: anteromedially directed (0); sinusoidal, posteromedially directed on its lateral half and anteromedially directed along its medial region (1); posteromedially directed (2), straight (3), posteromedially curved (U-shaped) (4).

**Character 127** (Pol, 1999a: char. 140): Nasal lateral border posterior to external nares: laterally concave (0), straight (1).

**Character 128** (Pol, 1999a: char. 141): Nasal lateral edges: nearly parallel (0); oblique to each other, converging anteriorly (1); or oblique to each other, diverging anteriorly (2).

**Character 129** (Pol, 1999a: char. 143): Palatine anteromedial margin: exceeding anterior margin of suborbital fenestrae: extending anteriorly between maxillae (0), not exceeding anterior margin of suborbital fenestrae (1).

**Character 130** (Pol, 1999a: char. 144): Dorsoventral height of jugal antorbital region with respect to infraorbital region equal or lower (0), antorbital region more expanded than infraorbital region (1).

**Character 131** (Pol, 1999a: char. 145): Maxilla-lacrimal contact in antorbital fossa: partially included (0), or completely included (1).^[[8]](#footnote-8)^

**Character 132** (Pol, 1999a: char. 146): Lateral eustachian tube openings, location: located posteriorly to medial opening (0), aligned anteroposteriorly and dorsoventrally (1).

**Character 133** (Pol, 1999a: char. 147): Anterior process of ectopterygoid: developed (0), reduced or absent (1).

**Character 134** (Pol, 1999a: char. 148): Posterior process of ectopterygoid: developed (0), reduced or absent (1).

**Character 135** (Pol, 1999a: char. 149 and Ortega et al., 2000: char. 13): Small foramen located in the lateral surface of premaxilla-maxilla suture in lateral surface (not for big mandibular teeth): absent (0), or present (1).

**Character 136** (Pol, 1999a: char. 150): Jugal posterior process, extent of process: exceeding posteriorly the infratemporal fenestrae (0), does not exceed infratemporal fenestrae (1).

**Character 137** (Pol, 1999a: char. 151): Compressed crown of maxillary teeth, orientation: oriented parallel to the longitudinal axis of skull (0), obliquely disposed (1).

**Character 138** (Pol, 1999a: char. 152): Large and aligned neurovascular foramina on lateral maxillary surface: absent (0), or present (1).

**Character 139** (Turner 2015, modified from Pol, 1999a: char. 153): External surface of maxilla and premaxilla, general shape: with single plane facing laterally (0), or with ventral region facing laterally and dorsal region facing dorsolaterally (1).

**Character 140** (Turner 2015, modified from Pol, 1999a: char. 154; Ortega et al., 2000: char. 104; Andrade and Bertini, 2008a: char. 135): Maxillary teeth, lateral compression: absent (0), present, compression asymmetrically occurring only along distal margin giving teeth a teardrop shape (1), present, lateral compression symmetrically developed (2).

**Character 141** (Pol, 1999a: char. 155): Posteroventral corner of quadratojugal: reaching quadrate condyles (0), does not reach quadrate condyles (1).

**Character 142** (Turner 2015, modified from Pol, 1999a: char. 156): Base of postorbital process of jugal, orientation: directed posterodorsally (0), directed dorsally (1), directed anterodorsally (2).

**Character 143** (Pol, 1999a: char. 157): Postorbital process of jugal, location on jugal: anteriorly placed (0), in middle (1), posteriorly positioned (2).

**Character 144** (Pol, 1999a: char. 158 and Ortega et al., 2000: char. 36): Postorbital-ectopterygoid contact: present (0), or absent (1).

**Character 145** (Pol, 1999a: char. 161): Quadratojugal, ornamentation: absent (0), ornamented at the base (1).

**Character 146** (Pol, 1999a: char. 162): Prefrontal-maxilla contact in the inner anteromedial region of orbit absent (0), present (1).

**Character 147** (Pol, 1999a: char. 163): Basisphenoid, exposure on braincase: without lateral exposure(0), with lateral exposure (1).

**Character 148** (Pol, 1999a: char. 165): Quadrate process of pterygoids: well developed (0), poorly developed (1).

**Character 149** (Turner 2015, modified from Pol, 1999a: char. 166 and Ortega et al., 2000: char. 44): Quadrate major axis, direction of orientation: posteroventrally (0), ventrally (1), anteroventrally (2).

**Character 150** (Pol, 1999a: char. 167): Quadrate distal end: with only one plane facing posteriorly (0); with two distinct faces in posterior view, a posterior one and a medial one bearing foramen aereum (1).

**Character 151** (Pol, 1999a: char. 168): Anteroposterior development of neural spine in axis: well developed, covering all of neural arch length (0); or poorly developed, located over posterior half of neural arch (1).

**Character 152** (Pol, 1999a: char. 169): Prezygapophyses of axis, development relative to neural arch: not exceeding edge of neural arch (0), exceeding the anterior margin of neural arch (1).

**Character 153** (Pol, 1999a: char. 170): Postzygapophyses of axis: well developed, curved laterally (0), poorly developed (1).

**Character 154** (Turner 2015, modified from Pol, 1999b: char. 212): Shape of dentary symphysis in ventral view: tapering anteriorly forming an angle (0); U-shaped, smoothly curving anteriorly (1); or lateral edges longitudinally oriented, convex anterolateral corner and extensive transversally oriented anterior edge (2).

**Character 155** (Pol, 1999b: char. 213): Unsculpted region in the dentary below the tooth row absent (0), present (1).

**Character 156** (Buckley and Brochu, 1999: char. 102): Surangular, contribution to the glenoid fossa: forms only the lateral wall of glenoid (0) forms approximately one-third of the glenoid (1).

**Character 157** (Turner 2015, modified from Buckley and Brochu, 1999: char. 102): Femur, anterior margin: linear (0), or bears flange for PIFI 1 musculature^[[9]](#footnote-9)^ (1).

**Character 158** (Turner 2015, modified from Buckley and Brochu, 1999: char. 105): Dentary, lateral surface: smooth lateral to seventh alveolus (0), with lateral concavity for the reception of the enlarged maxillary tooth (1).

**Character 159** (Turner 2015, modified from Ortega et al., 1996: char. 1 and Buckley and Brochu, 1999: char. 107): Dorsal edge of dentary: slightly concave or straight and subparallel to the longitudinal axis of skull (0), straight with an abrupt dorsal expansion, being straight posteriorly (1), with a single dorsal expansion and concave posterior to this (2); sinusoidal, with two concave waves (3).

**Character 160** (Turner 2015, modified from Ortega et al., 1996: char. 2 and Buckley and Brochu, 1999: char. 108): Dentary compression and ventrolateral surface anterior to mandibular fenestra: compressed and vertical (0), not compressed and convex (1).

**Character 161** (Ortega et al., 1996: char. 7 and Buckley and Brochu, 1999: char. 110): Splenial posterior to symphysis: thin (0), robust dorsally (1).

**Character 162** (Ortega et al., 1996: char. 13 and Buckley et al., 2000: char. 117): Cheek teeth: not constricted at base of crown (0), constricted (1).

**Character 163** (Ortega et al., 2000: char. 10): Ventral edge of premaxilla, location relative to maxilla: at the same height as the ventral edge of maxilla (0), located deeper, with the dorsal contour of anterior part of dentary strongly concave (1).

**Character 164** (Turner 2015, modified from Ortega et al., 2000: char. 19): Maxillary dental implantation: teeth in isolated alveoli (0), or located in dental groove (1). ^[[10]](#footnote-10)^

**Character 165** (Ortega et al., 2000: char. 24): Caudal tip of nasals: converge at sagittal plane (0), or separated by anterior sagittal projection of frontals (1).

**Character 166** (Ortega et al., 2000: char. 33): Relative length between squamosal and postorbital: squamosal is longer (0), or postorbital longer (1).

**Character 167** (Turner 2015, modified from Ortega et al., 2000: character 34): Jugal portion of postorbital bar, relative to lateral surface of jugal: flush with lateral surface (0), anteriorly continuous but posteriorly inset (1), medially displaced and a ridge separate postorbital bar from lateral surface of jugal (2).

**Character 168** (Ortega et al., 2000: char. 42): Outer surface of squamosal laterodorsally oriented: extensive (0), reduced and sculpted (1), reduced and unsculpted (2).

**Character 169** (Ortega et al., 2000: char. 47): Quadratojugal spine at posterior margin of infratemporal fenestra: absent (0), present (1).

**Character 170** (Turner 2015, modified from Ortega et al., 2000: char. 53): Quadrate condyles: poorly developed intercondylar groove (0); medial condyle expands ventrally, being separated from lateral condyle by a deep intercondylar groove (1).

**Character 171** (Ortega et al., 2000: char. 62): Exposure of supraoccipital in skull roof: absent (0), present (1).^[[11]](#footnote-11)^

**Character 172** (Ortega et al., 2000: char. 70): Nasal participation in antorbital fenestra: present (0), absent (1).^[[12]](#footnote-12)^

**Character 173** (Ortega et al., 2000: char. 75): Anterior opening of temporo-orbital canal in dorsal view: exposed (0); hidden and overlapped by squamosal rim of supratemporal fossa (1).^[[13]](#footnote-13)^

**Character 174** (Ortega et al., 2000: char. 90): Foramen intramandibularis oralis: small or absent (0), big and slotlike (1).

**Character 175** (Turner 2015, modified from Ortega et al., 2000: char 98): Coronoid size: short and located below the dorsal edge of the mandibular ramus (0); or anteriorly extended with posterior region elevated at dorsal margin of mandibular ramus (1).

**Character 176** (Ortega et al., 2000: char. 101): Width of root of teeth with respect to crown: narrower or equal (0), wider (1).

**Character 177** (Ortega et al., 2000: char. 109): Gap in cervico-thoracic dorsal armor: absent (0), or present (1).

**Character 178** (Ortega et al., 2000: char. 130): Lateral contour of snout in dorsal view: straight (0), or sinusoidal (1).

**Character 179** (Ortega et al., 2000: char. 138): Pterygoidean flanges: laminar and expanded (0), bar-like and elongate (1), or bar-like and poorly developed (2).

**Character 180** (Ortega et al., 2000: char. 146): Ectopterygoid medial process, shape: single (0), forked (1).

**Character 181** (Turner 2015, modified from Ortega et al., 2000: char. 157): Skull roof, shape in dorsal view: rectangular (0), trapezoidal (1).

**Character 182** (Ortega et al., 2000: char. 30): Prefrontal pillars when integrated in palate: pillars transversely expanded (0), transversely expanded in their dorsal part and columnar ventrally (1), longitudinally expanded in their dorsal part and columnar ventrally (2).

**Character 183** (Ortega et al., 2000: char. 21): Ventral edge of maxilla in lateral view: straight or convex (0), sinusoidal (1).

**Character 184** (Turner 2015, modified from Ortega et al., 2000: char. 156): Position of first enlarged maxillary teeth: second or third alveolus (0), fourth or fifth (1).

**Character 185** (Pol and Apesteguía, 2005: char. 180): Splenial-dentary suture at symphysis on ventral surface: v-shaped (0), transversal (1).

**Character 186** (Pol and Apesteguía, 2005: char. 181): Posterior peg at symphysis: absent (0), present (1).

**Character 187** (Pol and Apesteguía, 2005: char. 182): Posterior ridge on glenoid fossa of articular: present (0), absent (1).

**Character 188** (Turner 2015, modified from Pol et al., 2009; previously from Gomani, 1997: char. 46; Buckley et al., 2000: char. 113; Andrade and Bertini, 2008a: char 149): Cusps of teeth, number and conformation: one unique cusp (0); one main cusp with smaller cusps arranged in one row (1); one main cusp with smaller cusps arranged in more than one row (2); several cusps of equal size arranged in more than one row (3)^[[14]](#footnote-14)^

**Character 189** (Pol and Apesteguía, 2005: char. 184): Dorsal surface of mandibular symphysis: flat or slightly concave (0); strongly concave and narrow, trough-shaped (1).

**Character 190** (Pol and Apesteguía, 2005: char. 185): Medial surface of splenials posterior to symphysis: flat or slightly convex (0), or markedly concave (1).^[[15]](#footnote-15)^

**Character 191** (Pol and Apesteguía, 2005: char. 186): Choanal septum shape: narrow vertical bony sheet (0), T-shaped bar expanded ventrally (1), massive and blocky (2).

**Character 192** (Pol and Norell, 2004a: char. 164): Cross-section of distal end of quadrate: mediolaterally wide and anteroposteriorly thin (0), subquadrangular (1).

**Character 193** (Pol and Apesteguía, 2005: char. 188): Lateral surface of dentaries below alveolar margin, at mid to posterior region of tooth row: vertically oriented, continuous with rest of lateral surface of dentaries (0); flat surface exposed dorsolaterally, divided by ridge from rest of lateral surface of dentaries (1).

**Character 194** (Pol and Norell, 2004a: char. 165): Palatine-pterygoid contact on palate: palatines overlie pterygoids (0), palatines firmly sutured to pterygoids (1).

**Character 195** (Pol et al., 2004: char. 164): Ectopterygoid main axis orientation: laterally or slightly anterolaterally (0); or anteriorly, subparallel to the skull longitudinal axis (1).

**Character 196** (Wu et al., 1997: char. 103): Squamosal descending process: absent (0), present (1).

**Character 197** (Turner 2015, modified from Wu et al., 1997: char. 105): Development of distal quadrate body ventral to otoccipital-quadrate contact: distinct (0), incipiently distinct (1), indistinct (2).

**Character 198** (Wu et al., 1997: char. 106): Pterygoid flanges: thin and laminar (0); dorsoventrally thick, with pneumatic spaces (1).

**Character 199** (Wu et al., 1997: char. 108): Postorbital participation in infratemporal fenestra: almost or entirely excluded (0), bordering infratemporal fenestra (1).

**Character 200** (Wu et al., 1997: char. 109): Palatines, contribution to suborbital fenestra: form margin of suborbital fenestra (0), excluded from margin of suborbital fenestra (1).

**Character 201** (Wu et al., 1997: char. 110): Angular posterior to mandibular fenestra, location on mandible: widely exposed on lateral surface of mandible (0), shifted to ventral surface of mandible (1).

**Character 202** (Wu et al., 1997: char. 112): Posteroventral edge of mandibular ramus, shape: straight or convex (0), markedly deflected (1).

**Character 203** (Turner 2015, modified from Wu et al., 1997: char. 119): Quadrate ramus of pterygoid, width in ventral view: narrow (0), broad (1).

**Character 204** (Wu et al., 1997: char. 121): Pterygoids, contact on palate: not in contact anterior to basisphenoid on palate (0), pterygoids in contact (1).

**Character 205** (Turner 2015, modified from Wu et al., 1997: char. 122): Olecranon: well developed (0), absent (1).

**Character 206** (Wu et al., 1997: char. 123): Cranial table width with respect to ventral portion of skull: as wide as ventral portion (0), narrower than the ventral portion of the skull (1).

**Character 207** (Wu et al., 1997: char. 127): Depression on posterolateral surface of maxilla: absent (0), present (1).

**Character 208** (Turner 2015, modified from Wu et al., 1997: char. 128 by Pol et al., 2009): Paired anterior palatal fenestrae: absent (0), present (1).

**Character 209** (Pol and Norell, 2004a: char. 179): Paired ridges located medially on ventral surface of basisphenoid: absent (0), present (1).

**Character 210** (Pol et al., 2004a: char. 179): Ventral margin of infratemporal bar of jugal: straight (0), dorsally arched (1).

**Character 211** (Pol and Norell, 2004a: char. 180): Posterolateral end of quadratojugal, shape and relationship with quadrate: acute or rounded, tightly overlapping the quadrate (0); with sinusoidal ventral edge and wide and rounded posterior edge slightly overhanging the lateral surface of the quadrate (1).

**Character 212** (Pol and Norell, 2004a: char. 181): Quadrate body distal to otoccipital-quadrate, orientation of contact in posterior view: ventrally oriented (0), ventrolaterally oriented (1).

**Character 213** (Gasparini et al., 1993: char. 3): Wedge-like process of maxilla in lateral surface of premaxilla-maxilla suture: absent (0), present (1).

**Character 214** (Pol and Norell, 2004b: char. 181): Palpebrals: separated from (or weakly sutured to) lateral edge of frontals (0), extensively sutured to each other and to lateral margin of frontals (1).

**Character 215** (Pol and Norell, 2004b: char. 182): External surface of ascending process of jugal: exposed laterally (0), exposed posterolaterally (1).

**Character 216** (Turner 2015, modified from Pol and Norell, 2004b: char. 183 by Pol et al., 2009, and this paper): Longitudinal ridge on lateral surface of jugal below infratemporal fenestra: absent (0); present, running entire length of posterior process of jugal (1); present, running entire length of jugal (2).

**Character 217** (Pol and Norell, 2004b: char. 184): Dorsal surface of posterolateral region of squamosal: without ridges (0), with three curved ridges oriented longitudinally (1).

**Character 218** (Pol and Norell, 2004b: char. 185): Ridge along dorsal section of quadrate-quadratojugal contact: absent (0), present (1).

**Character 219** (Turner 2015, modified from Pol and Norell, 2004b: char. 186 by Pol et al., 2009): Sharp ridge on the surface of the angular: absent (0), present on the ventral-most margin (1), present along the lateral surface (2).

**Character 220** (Pol and Norell, 2004b: char. 187): Longitudinal ridge along the dorsolateral surface of surangular: absent (0), present (1).

**Character 221** (Pol and Norell, 2004b: char. 188): Dorsal surface of osteoderms ornamented with anterolaterally and anteromedially directed ridges (fleur de lys pattern of Osmólska et al., 1997): absent (0), or present (1).

**Character 222** (Pol and Norell, 2004b: char. 189): Cervical region surrounded by lateral and ventral osteoderms sutured to dorsal elements: absent (0), present (1).

**Character 223** (Pol and Norell, 2004b: char. 190): Appendicular osteoderms: absent (0), present (1).^[[16]](#footnote-16)^

**Character 224** (Ortega et al., 2000: character 72): Supratemporal fenestra: present (0), absent (1).

**Character 225** (Turner 2015, modified from Pol and Apesteguía, 2005: char. 220 and Pol et al., 2009): Flat ventral surface of internal nares septum: parallel sided (0), tapering anteriorly (1), expanding anteriorly (2).

**Character 226** (Turner 2015, modified from Pol and Apesteguía, 2005: char. 221): Perinarial fossa: restricted extension (0); extensive, with distinctly concave surface facing anteriorly (1); large concave surface facing anteriorly, projecting anteroventrally from external nares and opening toward alveolar margin (2); extremely large and well-developed, occupying nearly entire surface of premaxilla ventral to external naris (3).

**Character 227** (Turner 2015, modified from Sereno et al., 2001: char. 67): Premaxillary palate, circular paramedian depressions: absent (0); present, located anteriorly on premaxilla (1); present, located at premaxilla-maxilla suture (2).

**Character 228** (Pol and Apesteguía, 2005: char. 223): Nasals, shape of posterolateral region: flat surface facing dorsally (0); lateral region deflected ventrally, forming part of the lateral surface of snout (1).

**Character 229** (Zaher et al., 2006: char. 193): Lacrimal, posterior extent and relationship with jugal: lacrimal: extending posteroventrally, widely contacting the jugal (0); tapers ventroposteriorly, does not contact or contacts jugal only slightly (1).

**Character 230** (Zaher et al., 2006: char. 194): Jugal, large foramen on the lateral surface, near its anterior margin: absent (0), present (1).

**Character 231** (Turner 2015, modified from Zaher et al., 2006: char. 195): Procumbent premaxillary alveoli: absent (0), present (1).

**Character 232** (Turner 2015, modified from Martinelli, 2003: char. 36 by Zaher et al., 2006: char. 196 and Turner, 2004: char. 119): Palatines, orientation: run parasagittally along midline (0); diverge laterally becoming rod-like caudally forming palatine bars (1).

**Character 233** (Zaher et al., 2006: char. 197): Ectopterygoid, participation in the palatine bar: absent (0), present (1).

**Character 234** (Pol and Norell, 2004a: char. 183): Choanal opening: opened posteriorly and continuous with pterygoid surface (0), closed posteriorly by an elevated wall formed by the pterygoids (1).

**Character 235** (Zaher et al., 2006: char. 198): Ectopterygoid, extent of medial projection on the ventral surface of pterygoid flanges: barely extended (0); widely extended, covering approximately the lateral half of the ventral surface of the pterygoid flanges (1).

**Character 236** (Gasparini et al., 2006: char. 236): Evaginated maxillary alveolar edges: absent (0), present as continuous sheet (1), present as discrete evaginations at each alveolus (2).

**Character 237** (Gasparini et al., 2006: char. 237): Premaxilla, foramen in perinarial depression: absent (0), present (1).

**Character 238** (Sereno et al., 2001: char. 27): Frontal, anterior ramus with respect to anterior tip of the prefrontal: ending posteriorly (0), ending anteriorly (1).

**Character 239** (Turner 2015, modified from Sereno et al., 2001: char. 68): Premaxilla, anterior alveolar margin orientation: vertical (0), inturned (1).

**Character 240** (Turner 2015, modified from Sereno et al., 2001: char. 69): Premaxillary tooth row orientation: arched posteriorly from midline (0); angled posterolaterally, at 120◦ angle (1); transverse (2).

**Character 241** (Sereno et al., 2001: char. 70): Last premaxillary tooth position relative to tooth row: anterior (0), anterolateral (1).

**Character 242** (Gasparini et al., 2006: char. 242): Posterior teeth with rings of undulated enamel: absent (0), present (1).

**Character 243** (modified from Brochu, 1999: char. 108 by Gasparini et al., 2006: char. 243): Maxilla-palatine suture, shape of palatines: palatine anteriorly rounded (0), palatine anteriorly pointed (1), palatine invaginated (2).

**Character 244** (Gasparini et al., 2006: char. 244): Postorbital, lateral surface formed by: postorbital and jugal (0), only by postorbital (1).

**Character 245** (Gasparini et al., 2006: char. 245): Surangular groove, enlarged foramen at anterior end: absent (0), present (1).

**Character 246** (Gasparini et al., 2006: char. 246): Shape of antorbital fossa: subcircular or subtriangular (0); elongate, low, and oriented obliquely (1).^[[17]](#footnote-17)^

**Character 247** (Gasparini et al., 2006: char. 247): Prefrontal lateral development: reduced (0); enlarged, extending laterally over the orbit (1).

**Character 248** (Gasparini et al., 2006: char. 248): Foramen for the internal carotid artery: reduced, similar in size to openin gs for cranial nerves IX–XI (0); extremely enlarged (1).

**Character 249** (Gasparini et al., 2006: char. 249): Squamosal posterolateral region, lateral to paroccipital process: narrow (0), bearing subrounded flat surface (1).

**Character 250** (Gasparini et al., 2006: char. 250): Posteromedial branch of squamosal, orientation: transversely oriented (0), or posterolaterally oriended (1).

**Character 251** (Gasparini et al., 2006: char. 251): Squamosal, dorsal margin of occipital flange: straight (0), dorsally concave (1).

**Character 252** (Gasparini et al., 2006: char. 252): Sculpture in external surface of rostrum: absent (0), present (1).

**Character 253** (Gasparini et al., 2006: char. 253): Longitudinal depressions on palatal surface of maxillae and palatines: absent (0), present (1).

**Character 254** (Gasparini et al., 2006: char. 254): Angle between medial and anterior margins of supratemporal fossa: approximately 90 degrees (0), approximately 45 degrees (1).^[[18]](#footnote-18)^

**Character 255** (Gasparini et al., 2006: char. 255): Sacral vertebrate, direction of transverse process: laterally (0), markedly deflected ventrally (1).

**Character 256** (Gasparini et al., 2006: char. 256): Prefrontal and lacrimal around orbits: forming flat rims (0); evaginated, forming elevated rims (1).

**Character 257** (Gasparini et al., 2006: char. 257): Nasal bone s: paired (0), partially or completely fused (1).

**Character 258** (Brochu, 1997a: char. 3): Axial neural spines, width of posterior half: wide (0), narrow (1).

**Character 259** (Brochu, 1997a: char. 19): Axis hypapophysis, deep fork: present (0), absent (1).

**Character 260** (Brochu, 1997a: char. 27): Ulna, width of olecranon process: narrow and subangular (0), wide and rounded (1).

**Character 261** (Brochu, 1997a: char. 29): M. teres major and M. dorsalis scapulae: insert separately on humerus, scars can be distinguished dorsal to deltopectoral crest (0); insert with common tendon, single insertion scar (1).

**Character 262** (Turner 2015, modified from Brochu, 1997a: char. 53): Dentary, projection of anterior alveoli: project anterodorsally (0), weakly procumbent (1), strongly procumbent (2).

**Character 263** (Brochu, 1997a: char. 84): Squamosal, dorsal and ventral rims of squamosal groove for external ear valve musculature: parallel (0), squamosal groove flares anteriorly (1).

**Character 264** (Brochu, 1997a: char. 91): Ectopterygoid, contat with maxilla near toothrow: ectopterygoid abuts maxillary toothrow (0), maxilla broadly separates ectopterygoid from maxillary toothrow (1).

**Character 265** (Brochu, 1997a: char. 92): Shallow fossa at anteromedial corner of supratemporal fenestra: present (0); absent, anteromedial corner of supratemporal fenestra smooth (1).

**Character 266** (Turner 2015, modified from Brochu, 1997a: char. 103 by Pol et al., 2009): Lateral margins of frontal, relative to the skull surface: flush with skull surface (0); elevated, forming ridged orbital margins (1).

**Character 267** (Brochu, 1997a: char. 130): Laterosphenoid, orientation of capitate process: laterally oriented (0), anteroposteriorly oriented toward midline (1).

**Character 268** (Turner 2015, modified from Brochu, 1997a: char. 141 by Pol et al., 2009): Exoccipital, development of boss and paroccipital process: process lateral to cranioquadrate opening short (0), boss small or absent on paroccipital process, process lateral to cranioquadrate opening long (1).

**Character 269** (Turner 2015, modified from Norell, 1988: char. 32 by Brochu, 1997a: char. 149): Ectopterygoid, extent along lateral pterygoid flange, at maturity: extends to posterior tip of lateral pterygoid flange (0), or does not extend to posterior tip of lateral pterygoid (1).

**Character 270** (Turner 2015, modified from Brochu, 1997a: char. 153): Incisive foramen, location relative to premaxillary toothrow: foramen situated far from premaxillary toothrow, at the level of the second or third alveolus (0), abuts premaxillary toothrow (1), projects between first premaxillary teeth (2).

**Character 271** (Turner 2015, modified from Turner, 2006: character 126 by Pol et al., 2009, Pritchard et al. 2013): Ventral surface of choanal septum: smooth to slightly depressed (0), marked by an acute groove (1), vomeral septum divided into bilateral laminae (2).

**Character 272** (Turner 2015, modified from Turner, 2006 char. 128 by Pol et al., 2009): Proximal-most portion of fibular head: straight sided to weakly developed posteriorly (0); very sharply projecting posteriorly, forming distinct extension (1).

**Character 273** (Turner 2015, modified from Turner, 2006: char. 129): Cervical rib shaft, posterior process, posterodorsally projecting spine at the junction with tubercular process: absent (0), present (1). ^[[19]](#footnote-19)^

**Character 274** (Turner 2015, modified from Pol et al., 2009: char. 274): Longitudinal keels on dorsal surfaces of osteoderms: restricted to the posterior edge of osteoderm (0), not restricted to the posterior edge (1).

**Character 275** (Pol et al., 2009: char. 275): Jugal, anteriorly on lateral surface below orbits: lacks a depression (0), possesses a depression (1).

**Character 276** (Turner 2015, modified from Pol et al., 2009: char. 276, Pritchard et al, 2013): Transverse ridge crossing the frontal anteromedial to the orbits: absent (0), present as a ridge (1), resent as prominent anteriorly-curved shelf (transverse interorbital crest sensu Andrade and Hornung 2011) (2) ^[[20]](#footnote-20)^

**Character 277** (Pol et al., 2009: char. 277): Shallow hemispherical depression on the lacrimal and/or prefrontal anterior to orbital margin (not articulation facet for palpebral): absent (0), present (1). ^[[21]](#footnote-21)^

**Character 278** (Pol et al., 2009: char. 278): Anterior half of interfenestral bar between suborbital fenestrae: lateral margins are parallel to subparallel (0), flared anteriorly (1).

**Character 279** (Pol et al., 2009: char. 279): Posterior half of interfenestral bar between suborbital fenestrae: lateral margins are parallel to subparallel (0), flared posteriorly (1).

**Character 280** (Pol et al., 2009: char. 280): Angular, shape of posteroventral margin: straight or gently arched dorsally (0), strongly arched dorsally (1).

**Character 281** (Pol et al., 2009: char. 281): Squamosal, lateral margin of dorsal surface: squared off with continuous ear valve groove (0), bears a prominent depressed area just anterior to posterior lobe of squamosal, groove for ear valve discontinuous (1).

**Character 282** (Pol et al., 2009: char. 282): Fibula, shaft distal to iliofibularis trochanter: straight (0), bowed posteriorly (1).

**Character 283** (Buckley and Brochu, 1999: char. 106): Scapular blade width: no more than twice the length of the scapulocoracoid articulation (0); scapular blade very broad and greater than twice the length of scapulocoracoid articulation (1).

**Character 284** (Turner 2015, modified from Buckley et al., 2000: char. 115, Prichard et al. 2013): Vomer, exposure on palate: vomer contributes flattened plate to secondary palate (0), vomer forms no part of secondary palate (1).

**Character 285** (Turner and Buckley, 2008: char. 285): Supraoccipital, when present on dorsal skull roof: with narrow exposure, parietal forms portion of occipital surface (0); with broad exposure, parietal does not form portion of occipital surface (1).^[[22]](#footnote-22)^

**Character 286** (Turner and Buckley, 2008: char. 286): Jugal, anterior and posterior processes: inline dorsoventrally (0); anterior and posterior processes at a sharp angle to one another, both processes slope ventrally to form a strongly arched jugal (1).

**Character 287** (Turner and Buckley, 2008: char. 287): Lateral expansion of posterodorsal edge of surangular anterior to glenoid fossa: absent (0), present (1).

**Character 288** (Turner and Buckley, 2008: char. 288): In lateral view, anterior process of the squamosal extending to the orbital margin, overlapping the postorbital: absent (0), present (1).

**Character 289** (Turner and Buckley, 2008: char. 289): In lateral view, surangular and dentary suture: simple, with little or no interdigitation (0); suture complex, with interlocking prongs from both surangular and dentary, three posterior prongs from dentary and two from surangular (1).

**Character 290** (Turner and Buckley, 2008: char. 290): Prominent depression on palate near alveolar margin at level of sixth or seventh alveolus: absent (0), present (1).

**Character 291** (Andrade and Bertini, 2008a: char. 103): Pterygoid, ventral surface of pterygoid flanges, parachoanal fossae: absent (0), present (1).

**Character 292** (Turner and Sertich, 2010: char. 292): Pterygoid, in ventral view, participation in suborbital fenestra: pterygoid forms margin of suborbital fenestra (0), excluded from suborbital fenestra by ectopterygoid-palatine contact (1).

**Character 293** (Turner and Sertich, 2010: char. 293): Maxilla, lateral surface along alveolar margin, conformation of the neurovascular foramina: foramina absent or form a single continuous row (0), gap in foramina between an anterior series and a posterior series (1).

**Character 294** (Turner and Sertich, 2010: char. 294): Surface of tooth enamel: smooth or slightly crenulated (0), with ridges at base of crown (often extending apically) (1).

**Character 295** (Turner and Sertich, 2010: char. 295): Posterior (molariform) teeth, wear facets: absent (0), or present (1).

**Character 296** (Turner and Sertich, 2010: char. 296): Tooth (with transitional morphology) present at premaxilla-maxilla contact: absent (0), or present (1).

**Character 297** (Turner and Sertich, 2010: char. 297): Basioccipital, midline crest on basioccipital plate below occipital condyle: absent (0), or present (1).

**Character 298** (Turner and Sertich, 2010: char. 298): Dorsal osteoderms, accessory ranges of osteoderms (sensu Frey, 1988): absent (0), present (1).

**Character 299** (Andrade and Bertini, 2008a: char. 131): Maxillary tooth, size relative to maxillary palatal surface in palatal view: proportionally small teeth, occupying only marginal portion of ventral surface of maxilla (0); proportionally well developed teeth, occupying large area of maxillary palatal surface (1).

**Character 300** (Jouve, 2004: character 68; Jouve, 2009: character 75): Ventral lamina of jugal: extends far anterior to ectopterygoid (0), ends at the level of the ectopterygoid (1).

**Character 301** (Sereno and Larsson, 2009: character 199; adapted from Norell, 1988: character 42 and Brochu, 1997: character 51): Surangular extension toward posterior end of retroarticular process: along entire length (0), or pinched off anterior to posterior tip (1).

**Character 302** (Turner 2015): Muscle attachment scars on ventral surface of quadrate ramus: form modest crests (0); prominent knobs (1).

**Character 303** (Turner 2015): Pterygoid flange shape: mediolaterally broad, reaching laterally beyond medial margin of quadrate condyles (0); relatively narrow, does not reach laterally to medial margin of quadrate condyles (1).

**Character 304** (Turner 2015): In ventral view, posterior process of maxilla relative to ITF excluded from ITF (0); forms part of ITF (1).

**Character 305** (Turner 2015): Highly modified ectopterygoid, mediolaterally broad and flattened with greatly expanded: absent (0); present, robust anterior process larger than posterior process (1); present, anterior and posterior process roughly equal in size (2).

**Character 306** (Turner 2015): In ventral view, palate medial to toothrow: forms a single continuous surface (0); ridge running on the palate medial to toothrow formed by maxilla and ectopterygoid (1).

**Character 307** (Turner 2015): Maxillary tooth row, penultimate and ultimate maxillary teeth enlarged and highly modified crushing tooth: absent (0); present (1).

**Character 308** (Turner 2015): Prefrontals: do not meet at midline (0); meet at midline (1).

**Character 309** (Turner 2015): Pear shaped external naris: absent (0); present (1).

**Character 310** (Turner 2015): Skull, dorsal surface at parietal-squamosal contact surface: continuous across suture (0); suture marked by groove or sulcus (1).

**Character 311** (Turner 2015): Maxilla, lateral surface, continuous groove or sulcus extending from orbital margin to near orbital margin towards narial opening: absent (0); present (1).

**Character 312** (Turner 2015): Maxilla, posteromedial process curving posteriorly onto palatine formed nasopharyngeal passage: absent (0); present (1).

**Character 313** (Turner 2015): Squamosal, posterior half, dorsal and ventral rims of groove for external ear valve musculature: thin or parallel sided (0); flared posteriorly (1).

**Character 314** (Turner 2015): Lacrimal, in dorsal view, anterior extent on rostrum relative to prefrontal: prefrontal extends farther anteriorly (0); lacrimal extends farther anteriorly (1); lacrimal and prefrontal subequal in anterior extent (2).

**Character 315** (Turner 2015): Lacrimal, in dorsal view, mediolateral width relative to prefrontal: equal to or less than width of prefrontal (0); wider than prefrontal (1).

**Character 316** (Turner 2015): Premaxillae, degree of contact posterior to the incisive foramen: extensive contact (0); narrow contact (1).

**Character 317** (Turner 2015): Posterior margin of the palatines where they form the floor of the nasopharyngeal passage, shape: V- or U-shaped (0); straight (1).

**Character 318** (Turner 2015): Posterior margin of the choanal groove, location: anteriorly on the pterygoids (0); posteriorly on the pterygoids near the posterior margin of pterygoids (1).

**Character 319** (Turner 2015): Pterygoid-palatine contact, ventral aspect of palate, shape of the suture: transverse, or nearly so (0); prong of pterygoid projects anteriorly (1).

**Character 320** (Turner 2015): On palate, foramen located on premaxilla/maxilla suture near the alveolar border: absent (0); present (1).

**Character 321** (Turner 2015): Ectopterygoid/pterygoid contact in ventral view: complex, anterior part of ectopterygoid forming suture whereas posterior part of ectopterygoid overlaps the pterygoid (0); sutured along entire contact, no part of ectopterygoid overlapping pterygoid (1).

**Taxon list for phylogeny**

84 crocodylomorph taxa were used for the phylogenetic analysis, comprising all major taxonomic groups of fossil crocodylomorphs. The total number of taxa used here is smaller than in the original matrix of Turner (2015): because the focus of this study lay on the Neosuchian relationships of *Knoetschkesuchus langenbergensis* gen. nov. sp. nov., we left some of the more basal and more distally related taxa out, in particular if such taxa in larger clades are treated sufficiently. *Theriosuchus grandinaris* (Lauprasert et al. 2005) and *Knoetschkesuchus langenbergensis* gen. nov. sp. nov. were added to the matrix. Several modifications in the coding of *Theriosuchus guimarotae* (Schwarz & Salisbury 2002), here combined newly into *Knoetschkesuchus guimarotae*, became necessary, as the coding by Turner (2015) was insufficient and could be supplemented substantially by first-hand information by DS.

**Changes from the coding of Turner (2015)**

*Gracilisuchus stipanicicorum*

Character 285: **? to _**

*Terrestrisuchus gracilis*

Character 285: **? to _**

*Dibothrosuchus elaphros*

Character 285: **? to _**

*Orthosuchus stormbergi*

Character 12: **? to _**

Character 285: **? to _**

*Protosuchus richardsoni*

Character 11: ? to 0

Character 285: **? to _**

*Edentosuchus tienshanensis*

Character 102: **? to _**

Character 131: **? to _**

Character 172: **? to _**

Character 246: **? to _**

*Hsisosuchus chungkingensis*

Character 285: **? to _**

*Chimaerasuchus paradoxus*

Character 12: **? to _**

*Fruitachampsa callisoni*

Character 102: **? to _**

Character 131: **? to _**

Character 172: **? to _**

Character 246: **? to _**

*Peirosaurus torminni*

Character 12: **? to _**

Character 285: **? to _**

*Anatosuchus* minor

Character 12: **? to _**

Character 285: **? to _**

*Mahajangasuchus* insignis

Character 12: **? to _**

*Marillasuchus amarali*

Character 102: **? to _**

Character 131: **? to _**

Character 172: **? to _**

Character 246: **? to _**

*Yacarerani boliviensis*

Character 102: **? to _**

Character 131: **? to _**

Character 172: **? to _**

Character 246: **? to _**

Character 285: **? to _**

*Sphagesaurus huenei*

Character 102: **? to _**

Character 131: **? to _**

Character 172: **? to _**

Character 246: **? to _**

*Araripesuchus wegeneri*

Character 12: **? to _**

*Araripesuchus tsangastanga*

Character 12: **? to _**

*Pelagosaurus typus*

Character 285: **? to _**

Character 223: **? to 0**

*Steneosaurus bollensis*

Character 285: **? to _**

*Cricosaurus araucanensis*

Character 285: **? to _**

*Eutretauranosuchus delfsi*

Character 102: **? to _**

Character 285: **? to _**

*Goniopholis simus*

Character 102: **? to _**

Character 131: **? to _**

Character 172: **? to _**

Character 246: **? to _**

Character 285: **? to _**

*Goniopholis baryglyphaeus*

Character 102: **? to _**

Character 131: **? to _**

Character 172: **? to _**

Character 246: **? to _**

*Amphicotylus lucasii*

Character 102: **? to _**

Character 131: **? to _**

Character 172: **? to _**

Character 246: **? to _**

Character 285: **? to _**

*Sunosuchus junggarensis*

Character 12: **? to _**

Character 102: **? to _**

Character 131: **? to _**

Character 172: **? to _**

Character 246: **? to _**

*Calsoyasuchus valliceps*

Character 12: **? to 1**

Character 285: **? to _**

*Rugosuchus nongaensis*

Character 12: **? to 0**

Character 102: **? to _**

Character 131: **? to _**

Character 172: **? to _**

Character 246: **? to _**

Character 285: **? to _**

*Pholidosaurus decipiens*

Character 102: **? to _**

Character 131: **? to _**

Character 172: **? to _**

Character 246: **? to _**

Character 285: **? to _**

*Terminonaris robusta*

Character 102: **? to _**

Character 131: **? to _**

Character 172: **? to _**

Character 246: **? to _**

*Sarcosuchus imperator*

Character 102: **? to _**

Character 131: **? to _**

Character 172: **? to _**

Character 246: **? to _**

Character 285: **? to _**

*Dyrosaurus*

Character 11: **? to 0**

Character 102: **? to _**

Character 131: **? to _**

Character 172: **? to _**

Character 246: **? to _**

*Hyposaurus rogersii*

Character 102: **? to _**

Character 131: **? to _**

Character 172: **? to _**

Character 246: **? to _**

*Wannchampsus kirpachi*

Character 12: **? to _**

Character 102: **? to _**

Character 131: **? to _**

Character 172: **? to _**

Character 246: **? to _**

*Shamosuchus gjadochtaensis*

Character 12: **? to _**

Character 102: **? to _**

Character 131: **? to _**

Character 172: **? to _**

Character 246: **? to _**

Character 285: **? to _**

*Paralligator major*

Character 102: **? to _**

*Theriosuchus guimarotae* (see Schwarz & Salisbury 2003 for detailed description):

Character 12: **? to _**

Characters 19, 23, 26, 28, 29, 31, 42, 58, 60, 73, 76, 94, 98, 101, 133, 134, 138, 148, 156, 158, 161, 178, 181, 185, 189, 190, 193, 196, 203, 211, 215, 220, 221, 222, 226, 228, 230, 231, 234, 238, 242, 244, 249, 251, 253, 255, 263, 265, 266, 283, 286, 287, 288, 289, 291, 292, 293, 294, 295, 296, 300, 302, 305, 310, 312, 314, 315: **? to 0**

Characters 22, 91, 120: **1 to 0**

Characters 27, 30, 34, 48, 52, 62 72, 77, 87, 90, 119, 127, 128, 154, 206, 223: **? to 1**

Characters 37, 109, 126, 159: **? to 2**

Character 71: **? to 4**

Character 103: **? to 3**

Characters 143, 258, 297, 301: **0 to 1**

Characters 164, 171: **1 to 0/1**

*Theriosuchus symphiestodon* (Martin et al. 2010, Martin et al. 2014)

Character 22: **1 to 0**

Character 229: **? to 0**

Character 246: ? to _

*Theriosuchus pusillus*

Charracter 205: **? to 1**

Characters 221, 222: **? to 0**

Character 223: **? to 1**

Character 229: **0 to 1**

*Batrachomimus pastosboensis*

Character 12: **? to _**

Glen rose form

Character 102: **? to _**

Character 131: **? to _**

Character 172: **? to _**

Character 246: **? to _**

*Paluxysuchus newmanni*

Character 102: **? to _**

Character 131: **? to _**

Character 172: **? to _**

Character 246: **? to _**

Character 285: **? to _**

*Susisuchus anatoceps*

Character 12: **? to _**

Character 102: **? to _**

Character 131: **? to _**

Character 172: **? to _**

Character 246: **? to _**

*Hylaeochampsa vectiana*

Character 12: **? to _**

Character 102: **? to _**

Character 131: **? to _**

Character 172: **? to _**

Character 246: **? to _**

*Iharkutosuchus makadii*

Character 12: **? to _**

Character 102: **? to _**

Character 131: **? to _**

Character 172: **? to _**

Character 246: **? to _**

*Isisfordia duncani*

Character 223: **? to 1**

*Acynodon adriaticus*

Character 131: **? to _**

*Allodaposuchus precedens*

Character 102: **? to _**

Character 131: **? to _**

Character 172: **? to _**

Character 246: **? to _**

*Borealosuchus formidabilis*

Character 102: **? to _**

Character 131: **? to _**

Character 172: **? to _**

Character 246: **? to _**

*Boverisuchus vorax*

Character 102: **? to _**

Character 131: **? to _**

Character 172: **? to _**

Character 246: **? to _**

Character 223: **? to 1**

*Leidyosuchus canadensis*

Character 102: **? to _**

Character 131: **? to _**

Character 172: **? to _**

Character 246: **? to _**

Character 285: **? to _**

*Asiatosuchus germanicus*

Character 102: **? to _**

Character 131: **? to _**

Character 172: **? to _**

Character 246: **? to _**

*Crocodylus niloticus*

Character 102: **? to _**

Character 131: **? to _**

Character 172: **? to _**

Character 246: **? to _**

Character 223: **0 to 1**

*Alligator mississippiensis* (pers. obs.DS on MfN specimen)

Character 11: ? to 1

Character 12: 0 to _

Character 285: **? to _**

Character 223: **0 to 1**

*Diplocynodon hantonensis*

Character 102: **? to _**

Character 131: **? to _**

Character 172: **? to _**

Character 246: **? to _**

Character 223: **? to 1**

*Argochampsa krebsi*

Character 102: **? to _**

Character 131: **? to _**

Character 172: **? to _**

Character 246: **? to _**

*Eothoracosaurus mississippiensis*

Character 102: **? to _**

Character 131: **? to _**

Character 172: **? to _**

Character 246: **? to _**

*Gavialis gangeticus*

Character 102: **? to _**

Character 131: **? to _**

Character 172: **? to _**

Character 246: **? to _**

1. Character 11 and 12 are directly related to each other. If the nasal-lacrimal-contact is coded to be absent (1) in character 11, the nature of the nasal-lacrimal-contact is coded as not applicable in character 12 in our character list. [↑](#footnote-ref-1)
2. Fusion of the frontal is an ontogenetic feature (Mook 1961), so that we slightly modified this character and added „in mature specimens“. [↑](#footnote-ref-2)
3. Width of bar between orbit and supratemporal fossa is ontogenetically influenced (Mook 1961), so that we slightly modified this character and added „in mature specimens“. [↑](#footnote-ref-3)
4. This character is represented only with the coding „1“ or „?“ in all taxa. We therefore exclude it from the analysis. [↑](#footnote-ref-4)
5. (This character is misleadingly coded in Turner 2015 as: “Tail osteoderms: dorsal only (0), or completely surrounded by osteoderms (1)”) [↑](#footnote-ref-5)
6. This character refers to the presence of an antorbital fossa, which is only the case if an antorbital fenestra is present. Therefore, in taxa that are coded in character 67 (presence of antorbital fenestra) as character state 3 (absent), this character 102 must consequently be coded as – (inapplicable). [↑](#footnote-ref-6)
7. The last characters state „one (5)“ from Turner 2015 was omitted here, because there was no taxon that fullfilled it. [↑](#footnote-ref-7)
8. This character refers to the presence of an antorbital fossa, which is only the case if an antorbital fenestra is present. Therefore, in taxa that are coded in character 67 (presence of antorbital fenestra) as character state 3 (absent), this character 131 must consequently be coded as – (inapplicable). [↑](#footnote-ref-8)
9. In Turner 2015 this is described to be fort he „coccygeofemoralis musculature“, whereas originally it is assigned to PiFi I. Because of the direct correlation of this area with the m. puboischiofemoralis internus I (e.g., Romer 1923, Hutchinson 2000) we retain the specific name of this muscle here. [↑](#footnote-ref-9)
10. Both states can be developed in a crocyliform with the rostral maxillary teeth set in alveoli and the caudal ones sitting in a tooth groove. [↑](#footnote-ref-10)
11. Ontogenetically influenced character (Mook 1961): for example, the supraoccipital in *Theriosuchus guimarotae* and in *T. langenbergensis* is exposed in dorsal view in the juvenile specimen. [↑](#footnote-ref-11)
12. This character refers to the presence of an antorbital fossa, which is only the case if an antorbital fenestra is present. Therefore, in taxa that are coded in character 67 (presence of antorbital fenestra) as character state 3 (absent), character 172 must consequently be coded as – (inapplicable). [↑](#footnote-ref-12)
13. This character had some missing or wrongly placed words in Turner (2015) and was corrected here. [↑](#footnote-ref-13)
14. Characters state „multiple small cusps along edges of occlusal surface (4)” by Pol & Apesteguía 2005 is not coded for any taxon and therefore omitted from here. [↑](#footnote-ref-14)
15. This character will be omitted from the Analysis, because no taxon has characters state 1 [↑](#footnote-ref-15)
16. Appendicular osteoderms are defined as those not in connection with the axial musculature (Salisbury & Frey 2001), i.e., they lie laterally to the bi- or tetraserial paravertebral shield and coded in accordance to this definition here. [↑](#footnote-ref-16)
17. This character refers to the presence of an antorbital fossa, which is only the case if an antorbital fenestra is present. Therefore, in taxa that are coded in character 67 (presence of antorbital fenestra) as character state 3 (absent), this character 246 must consequently be coded as – (inapplicable). [↑](#footnote-ref-17)
18. The angle of the medial and the anterior margin of the supratemporal fossa is ontogenetically influenced in *Theriosuchus*: only larger specimens have the rounded rectangular shape, whereas small skulls have a much more slit-like supratemporal fossa. [↑](#footnote-ref-18)
19. Turner 2015 lists a „State 2“ for this character that is neither specified nor coded and therefore omitted here. [↑](#footnote-ref-19)
20. Character state 3 „present as anteroposteriorly-oriented crest on frontal“ was removed, as it was not present in any of the coded taxa in the origimal matrix of Turner (2015) [↑](#footnote-ref-20)
21. Remark in Turner 2015: „Reinterpretation of the morphology in *Shamosuchus djadochtaensis* suggests that the depression coded for in this character is very similar to the depression present for articulation of a palpebral. Additional work is needed to clarify this morphology. Until then, we have chosen to exclude this character during tree searches.” [↑](#footnote-ref-21)
22. This character relates to Character 171: if the coding in character 171 is 0 (for no exposure of supraoccipital on skull roof), then coding in 285 is _ (inapplicable). [↑](#footnote-ref-22)
